# Veterinary nursing in the United Kingdom: Identifying the factors that influence retention within the profession

**DOI:** 10.3389/fvets.2022.927499

**Published:** 2022-11-23

**Authors:** Andrea Jeffery, Eleanor Taylor

**Affiliations:** ^1^Bristol Veterinary School, University of Bristol, Bristol, United Kingdom; ^2^Linnaeus Veterinary Limited, Bristol, United Kingdom; ^3^Royal College of Veterinary Surgeons, London, United Kingdom

**Keywords:** veterinary nursing, retention, job satisfaction, career progression, employer support

## Abstract

The UK regulatory body for registered veterinary nurses (RVNs), the Royal College of Veterinary Surgeons (RCVS), maintains the professional register of RVNs. Every year, a proportion fail to re-join the register. This research aimed to identify the factors that predict retention and to make recommendations to inform the Royal College of Veterinary Surgeons as the regulator as well as both the veterinary nursing and veterinary professions. An analysis of the raw quantitative data generated by the 2014 and 2019 RCVS surveys of the veterinary nurse profession was undertaken using multivariable logistic regression analysis. First, bivariable associations were estimated using unadjusted odds ratios to explore whether there are any (unconditional) associations between each predictor and the outcome. Predictors that were significant unconditional predictors of intention to leave were then entered into a multivariable logistic regression, yielding adjusted odds ratios. Quantitative analysis found significant relationships between intention to leave and the following factors in both the 2014 and 2019 survey data: job satisfaction; believing that veterinary nursing offers good opportunities for career progression; satisfaction with employer support; and having a second job. The following factors were significant in 2014, but not in 2019: undertaking nurse clinics, feeling valued, and being respected by veterinary surgeons. The factors that influence the retention of registered veterinary nurses (RVNs) within the profession are multifactorial and individual. Nurses are responsible for ensuring that those they work with are aware of their skill set and that they themselves are accountable for utilisation of those skills. When veterinary nurses are supported by their employers, they are more likely to stay in the profession. Having a clearly defined career structure and mapped routes for progression will be helpful with retention. A sense of job satisfaction was another important factor in retention. A pay structure linked to a career pathway framework, such as in human-centred nursing, is an area for further work.

## Introduction

There is evidence that the number of RVNs in the United Kingdom has declined in recent years (1–3). It is important that the number of RVNs grows in line with the number of veterinary surgeons to ensure that there are sufficient registered professionals to deliver the nursing care needed for veterinary patients. Therefore, the overarching aim of this research was to determine the factors that predict retention rates within the veterinary nursing profession and to set out a series of recommendations that could improve retention. The objective of the research was to critically analyse the views of the veterinary nursing profession (through the analysis of the 2014 and 2019 large-scale surveys of the veterinary nursing profession in the UK) to determine the factors that influence retention within the profession.

In the United Kingdom (UK), the regulatory body for both veterinary nurses and veterinary surgeons is the Royal College of Veterinary Surgeons (RCVS). The RCVS maintains the professional register for veterinary nurses. In 2021, 19,078 registered veterinary nurses (RVN) were listed. To remain on the register each year, a veterinary nurse must adhere to the registration requirements which include payment of the retention fee. If this is not paid, they are removed from the register. Since 2008, there has been no change in the pattern of removals from and restorations to the register due to non-payment of retention fees, with the number of removals being consistently higher than the number of restorations (Supplementary Figure S1). There is, therefore, clear evidence of a trend up to and including 2021 of veterinary nurses choosing not to remain on the professional register rather than indicating an intention to leave the profession but maintaining their registration. This negative trend in addition to the lack of robust data and limited previously published literature on the subject was a catalyst for undertaking this research aimed at determining what specific factors impact veterinary nurses and cause this net loss from the profession which is detrimental to the entire profession.

The overarching aim of this research was to identify the factors that influence retention and disseminate this information to the RCVS as the regulator, to veterinary nursing and veterinary professionals in their capacity as members of the veterinary team, and to employers and educators. This was to raise awareness and make recommendations for positive change based on the research findings in anticipation that changes made will positively impact veterinary nurse retention rates in future. The level of retention within both the veterinary nursing and veterinary professions is vital to ensure that patient care and therefore animal welfare are not compromised and the findings of this and other research in this area (4, 5) are important in highlighting the issues concerning this important subject.

## Materials and methods

A literature search of Ovid, PubMed, and CINHAL databases was conducted. Searches included combinations of the following keywords: “nurses,” “veterinary nurses,” “dental technicians,” “dental nurses,” “veterinary,” “veterinary surgeons,” “retention,” “job satisfaction,” “career,” “career theory,” and “career development.” The time frame for the publications was between the years 1990 and 2021. This period relates to a time of significant change within the veterinary nursing profession and therefore captures the evidence from other key professional areas with areas of commonality concerning staff retention rates.

The search results were examined, and only papers concerning the research area were selected using manual screening by the researcher. It was important that the review reflected any factors identified both internationally and nationally that the researcher deemed relevant to veterinary nursing within the UK in order to position the review within the context of what is already known about the subject globally. Therefore, articles regarding human-centred nursing internationally were included. The international perspective is important to avoid assumptions that the issues relating to retention may just be a national issue or that there are different problems affecting the national workforce than the international workforce.

Published work regarding dental technicians and dental nurses within the UK was included, the rationale being that there is similar professional development of both dental nurses and dental technicians in the UK to that of veterinary nurses. A high proportion of all these groups works within private small businesses, which again is similar to veterinary nurses. The results of the Royal College of Veterinary Surgeons manpower surveys of 2008, 2010, 2014, and 2019 of the veterinary and veterinary nursing professions were also reviewed.

The literature revealed that there are multiple factors that influence retention rates within veterinary nursing (1, 2, 4, 6), human-centred nursing (7–24), and dental nursing (25–27). Some of these factors were also reflected in the literature on retention of veterinary surgeons within the veterinary profession (2, 28–30). The different factors are summarised in Table 1 for each professional group discussed in the preceding review.

**Table 1:** Summary of factors influencing retention rates within veterinary, human-centred, and dental nursing.

**Professional group**	**Factors influencing retention**
Veterinary nurses	•Not feeling valued, respected, or recognised in the role. •Lack of career opportunities, frustration with poor pay, having a second job, and work patterns not being family friendly. •Those in non-clinical roles had higher salaries. •**Positive factor** related to the work environment and the satisfaction gained in working with veterinary patients and clients.
Human-centred nurses	•Job satisfaction, level of education, age, and gender of the professional. •Workplace environment including lack of support and mentoring. •Workload, staff–patient ratios, and burnout plus a desire for part-time working patterns. •Lack of autonomy and empowerment. •Those who were highly skilled were leaving to move into other commercial areas such as the pharmaceutical industry to use their skills more effectively. •**Positive factors**: a higher salary, a feeling that there were good organisational and career structures •New graduates felt that the 12-month post-registration preceptorship programmes were positive in terms of early careers support.
Dental nurses	•A lack of responsibility, respect, and understanding of the role by dentists. •Poor work conditions. •**Positive factors**: Continued professional development and career opportunities.
Veterinary surgeons	•Lack of career opportunities, not being valued and respected within the workplace, dissatisfaction with veterinary work. •Being “*fed up”* with the way the profession is going. •**Positive factor**: the work itself provides job satisfaction.

The factors influencing retention cross not only continents, but also the different professions, and are expressed in a variety of ways. However, the descriptions of the experiences, regardless of the different terminology used within the literature, are similar across all areas of nursing and veterinary medicine. Negative factors that influence retention include working conditions and pay, a lack of respect and accountability, a lack of empowerment, and feeling undervalued. For veterinary nurses and veterinary surgeons, alike the work environment including working with animals was a positive factor that brought satisfaction.

The literature review relating to veterinary nursing is based mainly on the RCVS surveys of the profession (1, 2, 4). Another key aspect important to note, also apparent in the work by Halter et al. (31), is that the literature including the RCVS surveys, focuses on the intention to leave and does not collect or report feedback from those who have actually left the profession.

The target population for the quantitative data were RCVS registered veterinary nurses who responded to the 2014 and 2019 RCVS surveys of the Veterinary Nurse Profession. Copies of both surveys are available in Supplementary material.

The 2014 survey was circulated electronically between April and May 2014 by the Institute for Employment Studies (IES) on behalf of the RCVS and was sent to all veterinary nurses and student veterinary nurses registered with the RCVS (*n* = 17,729) (28). Of the 5,496 veterinary nurses who completed this survey, 4,668 (84.9%) who were currently working within the profession answered the question regarding whether they intended to stay or leave the profession (Table 2). Of these, 82 were eliminated as they were intending to retire. The full number used for the quantitative aspect of the research was therefore 4,586. The dependent variable (intention to stay in the profession for the foreseeable future) used in the analysis was coded 0 for responses (*n* = 3,862, 84.2% of the full sample) and coded 1 for responses of intention to leave as soon as possible, in the next year or in the next 5 years (*n* = 724, 15.8%).

**Table 2:** Intention to stay or leave nursing.

	**2014**		**2019**	
**Response**	**N**	**Percent**	**N**	**Percent**
Stay in the profession for more than 5 years (including partial retirement)	-	-	4737	72.0%
Stay in profession for the foreseeable future	3862	82.7%	-	-
Fully retire within the next year	12	0.3%	15	0.2%
Fully retire within the next 1 to 2 years	-	-	38	0.6%
Fully retire within the next 3 to 5 years	-	-	157	2.4%
Fully retire in next five years	70	1.5%	-	-
Leave profession within the next year (non-retirement)	-	-	221	3.4%
Leave profession as soon as possible (non-retirement)	89	1.9%	-	-
Leave profession in the next year (non-retirement)	124	2.7%	-	-
Leave profession within the next 1 to 2 years (non-retirement)	-	-	488	7.4%
Leave profession within the next 3 to 5 years (non-retirement)	-	-	923	14.0%
Leave profession in the next 5 years (non-retirement)	511	10.9%	-	-
**Total “stay in profession”**	**3862**	**82.7%**	**4737**	**72.0%**
**Total leave (except for retirement)**	**724**	**15.5%**	**1632**	**24.8%**
**Total**	**4668**		**6579**	

The 2019 survey was circulated electronically between June and July 2019. There were no follow-up reminders for either the 2014 survey or the 2019 survey. The total sample size was 7,686, which represented a response rate of 29% (*n* = 26,503). Of the 7,686 veterinary nurses who completed this survey, 6,579 (84.9%) who were currently working within the profession answered the question regarding whether they intended to stay or leave the profession (Table 2). Of these, 210 were eliminated as they were intending to retire. The full number used for the quantitative aspect of the research was therefore 6,369. The dependent variable (of intention to stay in the profession for the foreseeable future) used in the analysis was coded 0 for responses (*n* = 4,737, 72% of the full sample) and coded 1 for responses of intention to leave as soon as possible, in the next year or in the next 5 years (*n* = 1,632, 24.8%).

Thirteen questionnaire responses associated with the likelihood of leaving the profession were used [based on the findings of the literature review (Table 1)]. Univariable logistic regression was carried out on each of these predictor variables. The outcome variable “planning to leave profession” was defined as respondents who answered that they were planning to leave veterinary nursing within the next 5 years for reasons other than retirement. Respondents who answered that they planned to retire were removed from the analysis.

### Data analyses

The data sets selected for analysis from the 2014 and 2019 RCVS surveys related to key factors, which have impacted the retention of nurses in the veterinary, human-centred, and dental professions nationally and internationally, as well as veterinary surgeons. These were age, level of education, job satisfaction, support from employers, respect, and recognition, being valued, salary level, and career progression (2, 3, 9, 11–13, 15, 18, 22, 25).

Supplementary Table S1 lists the survey questions and associated subject area in the 2014 and 2019 RCVS surveys, which were used for the secondary data set and evidenced through the literature review. Binary coding was chosen within the analysis as within a five-point Likert scale with a range of options from strongly agree, agree, neither agree nor disagree, disagree, and strongly disagree. Strongly agree vs. agree and strongly disagree vs. disagree can be interpreted differently by respondents who have, for example, different levels of experience or backgrounds; therefore, collapsing this scale into binary agree/disagree is the rationale for the decision to do this. This resulted in strongly disagree and disagree = 0 with agree and strongly agree = 1 (29). The option in the Likert scale of “neither agree nor disagree” was neutral and coded as NA. The relative risk ratio (RR) for intention to leave was calculated for each variable in both surveys, although this could not be calculated for age as it was not coded into a categorical variable.

The quantitative data were exported from the survey completed online, analysis was carried out using the Statistical Package for the Social Sciences (SPSS), and multivariable logistic regression analysis was used to explore the outcome of intention to leave the profession. First, bivariable associations were estimated using unadjusted odds ratios to explore whether there were any (unconditional) associations between each predictor and the outcome. Predictors that were significant unconditional predictors of intention to leave (Supplementary Tables S2, S3) were then entered in a multivariable logistic regression, yielding adjusted odds ratios. The adjusted odds ratios then show the conditional association when other predictors are held constant. This helps to separate out the independent effect of each predictor on the outcome, how it affects the outcome when people are effectively “equalised” in terms of the other covariates (e.g., same age and same qualifications). Supplementary Table S1 summarises how each of the variables was converted from a five-point Likert scale into a dichotomous scale (0 and 1) and the risk ratio for each variable. Supplementary Table S4 shows the odds ratio (OR) for each variable.

As intention to leave the profession was categorised as a dichotomous variable, with being more-or-less likely to leave the profession determining this dichotomy, binary logistic regression was conducted for the multivariable analysis. Predictor variables with *p*-values < 0.05 were then included in a multivariable logistic regression model to obtain the adjusted odds ratios for each predictor. There is some contention in the literature about the correct *p*-value to use when choosing predictor variables for a multivariable logistic regression; however, we chose to use *p* < 0.05 to obtain greater accuracy of the multivariable logistic analysis which can be achieved with a reduced set of variables (30, 32). The age of respondents was included in this model. The unadjusted odds ratios for the full and complete samples are given in Supplementary Table S4. These show that the point estimates of the odds ratios (OR) are similar in the two columns and conclusions about statistical significance do not differ greatly.

An important issue in the quantitative analysis is that of missing item responses on the predictor variables. As given in Table 3, among the *n* = 4,586 full sample, missing data varied from 0.4% of the variable indicating a second job to 42% of the variable on continued professional development (CPD). Overall, just 1,058 cases (23% of the full sample) had full information on all the predictor variables.

**Table 3:** 2014 Unadjusted and adjusted odds ratios.

**Variable**	**Unadjusted odds ratios**				**Adjusted odds ratios**			
	**(no missing data**, ***n*** = **1058)**				**(no missing data**, ***n*** = **1058)**			
	**OR**	**Lower 95% CI**	**Upper 95% CI**	***p*-value**	**OR**	**Lower 95% CI**	**Upper 95% CI**	***p*-value**
Lack of respect	3.126	2.253	4.337	<0.001^***^	0.570	0.368	0.882	0.012^**^
Having a second job	1.733	1.186	2.533	0.004^**^	1.828	1.163	2.873	0.009^**^
Undertakes nurse clinics regularly	0.595	0.425	0.834	0.003^**^	0.581	0.394	0.856	0.006^**^
Salary satisfaction	0.333	0.226	0.491	<0.001^***^	0.655	0.407	1.053	0.081^*^
Valued by vet surgeons	0.249	0.179	0.347	<0.001^***^	0.599	0.386	0.929	0.022^**^
Satisfied with employer support	0.226	0.162	0.316	<0.001^***^	0.505	0.334	0.763	0.001^**^
Good opportunity for career progression	0.216	0.151	0.310	<0.001^***^	0.296	0.194	0.454	<0.001^***^
VN gives me job satisfaction	0.080	0.048	0.135	<0.001^***^	0.158	0.086	0.292	<0.001^***^

*** P-value < 0.001.

** P-value < 0.05.

* P-value < 0.1.

To investigate how appropriate the assumption of data missing at random is, the descriptive statistics for both the full sample and complete case sample are laid out in Table 4, in total and broken down by the values of the dependent variable.

**Table 4:** 2019 Unadjusted and adjusted odds ratios.

**Variable**	**Unadjusted odds ratios**				**Adjusted odds ratios**			
	**(no missing data**, ***n*** = **1,731)**				**(no missing data**, ***n*** = **1,731)**			
	**OR**	**Lower 95% CI**	**Upper 95% CI**	***p*-value**	**OR**	**Lower 95% CI**	**Upper 95% CI**	***p*-value**
Having a second job	1.776	1.316	2.396	0.000^***^	1.577	1.107	2.247	0.012^**^
Time off (paid) for CPD	0.557	0.428	0.724	0.000^***^	0.899	0.656	1.231	0.505
Satisfied with salary	0.215	0.157	0.293	0.000^***^	0.475	0.334	0.675	<0.001^***^
Satisfied with employer support	0.182	0.142	0.233	0.000^***^	0.431	0.317	0.587	<0.001^***^
Good opportunity for career progression	0.158	0.119	0.211	0.000^***^	0.305	0.223	0.418	<0.001^***^
VN gives me job satisfaction	0.100	0.071	0.140	0.000^***^	0.240	0.162	0.354	<0.001^***^

*** P-value < 0.001.

** P-value < 0.05.

The full sample, 4,586, is of those who answered question E4 within the 2014 survey regarding career plans—from which the outcome variable of “planning to leave the profession” is derived.

The complete case sample, 1,058, is of those who answered the outcome variable as well as all the predictor variables.

The missing at random assumption was supported if there is a similarity between the full and complete case samples in (a) the distribution of each predictor variable and (b) the per cent intending to leave the profession within each level of the predictor variable.

Supplementary Table S2 (the 2014 survey data) illustrates the closeness of the percentage of selected numbers (a) in the total columns (full and complete samples) and (b) in the two Leave VN columns. The fact that these percentages are so similar is consistent with the proposition that missing responses are missing at random. Examples of those variables which are best aligned with a 2% or less difference relate to a lack of respect, having a second job, employer support, and HE qualification. Those with a 3% or more difference include client expectations and demands, salary satisfaction, and career progression.

There may still be systematic differences between the two samples that are not measured by these specific predictor variables that could lead to bias in the results. However, there is little evidence of bias here, and the sample of (*n* = 1,058) provides the data needed for the logistic regression analysis that was undertaken.

The closeness of the percentage of selected numbers in the full sample to those within the complete data (*n* = 1,058) and the full sample (*n* = 4,586) highlights that the researcher did not find strong evidence that the complete case sample differed systematically from the full sample. The fact that these percentages are so similar is consistent with the proposition that the missing responses are missing at random due to human error in missing a box within the question in the survey as described in the following.

Supplementary Table S3 illustrates the closeness of the percentage of selected numbers (a) in the total columns (full and complete samples) and (b) in the two Leave VN columns in the 2019 survey data.

## Results

A comparison was undertaken between 2014 and 2019 adjusted odds ratios (Table 5). Four variables were significant in the adjusted models in both 2014 and 2019, and those were having a second job, job satisfaction, satisfaction with employer support, and believing that veterinary nursing offers good opportunities for career progression.

**Table 5:** Comparison of 2014 and 2019 multivariable models (adjusted odds ratios).

**Variable**	**2014**				**2019**			
	**OR**	**Lower 95% CI**	**Upper 95% CI**	***p*-value**	**OR**	**Lower 95% CI**	**Upper 95% CI**	***p*-value**
Having a second job	1.828	1.163	2.873	0.009^**^	1.577	1.107	2.247	0.012^**^
Lack of respect from veterinary surgeons/employers is a “main challenge”	0.570	0.368	0.882	0.012^**^	n/a	n/a	n/a	n/a
Lack of respect from employers is a “main challenge”	n/a	n/a	n/a	n/a	1.136	0.796	1.621	0.483
Lack of respect from veterinary surgeons is a “main challenge”	n/a	n/a	n/a	n/a	0.904	0.585	1.396	0.648
Undertakes nurse clinics regularly	0.581	0.394	0.856	0.006^**^	n/s	n/s	n/s	n/s
Client expectations are a “main challenge”	n/s	n/s	n/s	n/s	1.026	0.760	1.385	0.869
Time off (paid) for CPD	n/a	n/a	n/a	n/a	0.899	0.656	1.231	0.505
Age	n/s	n/s	n/s	n/s	0.990	0.973	1.007	0.244
Satisfied with salary	0.655	0.407	1.053	0.081^*^	0.475	0.334	0.675	<0.001^***^
Valued by vet	0.599	0.386	0.929	0.022^**^	0.923	0.661	1.289	0.639
Satisfied with employer support	0.505	0.334	0.763	0.001^**^	0.431	0.317	0.587	<0.001^***^
Good opportunity for career progression	0.296	0.194	0.454	<0.001^***^	0.305	0.223	0.418	<0.001^***^
VN gives me job satisfaction	0.158	0.086	0.292	<0.001^***^	0.240	0.162	0.354	<0.001^***^

n/a = not asked.

n/s = not included in the model because univariable analysis did not find to be significantly associated with intention to leave.

*** P-value < 0.001.

** P-value < 0.05.

* P-value < 0.1.

The following variables were significant in 2014, but not in 2019:

Undertaking nursing clinics (this was asked in the same way in 2014 and 2019), feeling valued, and being respected by vss (this question was asked differently in 2019). in 2014, this was a single question about respect from employers/veterinary surgeons, while in 2019 this was separated into two separate questions to employers and veterinary surgeons. therefore, we cannot draw conclusion from the differing results from 2014 and 2019.

The following variable was significant in 2019, but not in 2014, and this was “client expectations are a main challenge.”

In 2014, respondents who had a second job were 1.82 times more likely to plan to leave than those who do not have a second job (OR: 1.83, 95% CI: 1.16–2.87, *p*-value: 0.009).

Interpersonal relationships also were an important factor in veterinary nurses' decision to leave the career. Veterinary nurses who felt valued by surgeons were 41% less likely to want to leave the profession (OR: 0.60, 95% CI: 0.38–0.92, *p*-value: 0.022), and those who felt respected by veterinary surgeons were 43% less likely to want to leave the profession (OR: 0.570, 95% CI: 0.36–0.88, *p*-value: 0.012). This is also the case with veterinary nurses who were satisfied with their employer support. These nurses were 50% less likely to want to leave the profession than veterinary nurses who were not satisfied with their employer support (OR: 0.51, 95% CI: 0.33–0.76, *p*-value: 0.001).

A lack of good opportunities for career progression was identified as an important factor in leaving the profession, with those with the opportunity for career progression 71% less likely to leave (OR: 0.30, 95% CI: 0.19–0.45, *p*-value: < 0.001). Furthermore, those who had a sense of job satisfaction, with those who had a sense of job satisfaction 85% more likely to stay in the profession than those who do not (OR: 0.16, 95% CI: 0.08–0.29, *p*-value: < 0.001).

Being able to undertake nurse clinics was an important factor for staying in the profession with those given the opportunity to undertake nurse clinics 42% less likely to leave (OR: 0.58, 95% CI: 0.39–0.85, *p*-value: 0.006).

In 2019, much like in 2014, having a second job was associated with an increased likelihood of intending to leave the veterinary nursing profession. Respondents with a second job were 1.58 times more likely to intend to leave (OR: 1.58, 95% CI: 1.107–2.247, *p*-value: 0.012). In 2019, those who agreed that veterinary nursing work gives them job satisfaction were 76% less likely to leave the profession (OR: 0.24, 95% CI: 0.162–0.354, *p*-value: < 0.001). Similarly, those believing that veterinary nursing offers good opportunities for career progression were 70% less likely to intend to leave (OR: 0.31, 95% CI: 0.223–0.418, *p*-value: < 0.001).

Satisfaction with employer support was also found to be an important factor, and those satisfied with the support given to them by their employer were 57% less likely than those not satisfied to intend to leave the profession (OR: 0.43, 95% CI: 0.317–0.587, *p*-value: < 0.001).

Satisfaction with salary level was found to be strongly associated with intention to leave the veterinary nursing profession. This was not significantly associated in 2014, but in 2019, and those satisfied with their salary level were 53% less likely to intend to leave veterinary nursing (OR: 0.48, 95% CI: 0.334–0.675, *p*-value: < 0.001).

## Discussion

Four variables were found to be significant predictors of retention in both the 2014 and 2019 studies. Respondents with a second job ~ 1.6 to 1.8 times more likely to leave the profession, while 70% were less likely to leave if they believed their career could progress. Similarly, around 75–85% of respondents were motivated to stay if they experienced job satisfaction. However, employer support lagged the other predictors, with 50–60% indicating that employer support was a determinant for not wanting to leave the profession.

There were other significant predictors to stay within the profession, but they did not emerge from both studies. In 2019, salary was significant with those who were satisfied with salary 53% less likely to intend to leave. Pay and conditions also featured as issues relating to retention within human-centred nursing, particularly around staffing shortages, overtime, and paid time off (14), with an increase in retention being linked to an increase in salary (9). Salary is also a reason cited by veterinary surgeons around intention to leave (2, 5). Salary may not have been significant in 2014 as the financial climate was more stable than in 2019.

The key conclusions are that the factors that predict the retention of nurses within the profession are both multifactorial and individual in nature. Nurses themselves are responsible for ensuring that those they work with are aware of their skill set and that they use their full skill sets within their roles (33). Age *per se* is not a factor for staying or leaving; this is similar within the veterinary profession but different from human-centred and dental nursing where age is a factor. The education route into the profession is not significant in terms of retention, but there are some aspects of the HE curriculum that could be added to the FE curriculum to help build professional resilience in the areas of problem solving and clinical decision-making.

There is a relationship between being valued and respected by veterinary surgeons. Having respectful colleagues was identified as being important in terms of job satisfaction within other veterinary nursing research (7). In addition, when veterinary nurses are supported by employers, they are more likely to stay in the profession. Employer support was also key to job satisfaction and retention within human-centred nursing (12, 18, 22).

Having a defined career framework with clearly mapped progression routes, similar to those within the National Health Service (NHS) for human-centred nurses will be helpful not only for veterinary nurses but also for the veterinary team, as well as for employers. This will enable all concerned to understand the different levels of achievement and required skills at each level of a career framework to facilitate the teams working more effectively together. Within human-centred nursing, those who were career orientated were identified as having greater job satisfaction (19).

Having a sense of job satisfaction was an important factor in retention. This finding was supported by human-centred studies which identified that being able to use initiative, being empowered within the workplace, and having autonomy are also key to job satisfaction (9, 11, 22).

The one area of autonomous practise that might have been linked to job satisfaction was performing minor surgical procedures, but there was no evidence that this was associated with the likelihood of planning to leave the profession. Having a second job was evidence of intention to leave, and this finding was also reflected in other veterinary nursing research (7) and so pay structure linked to career pathway structures similar to human-centred nursing and teaching is an area for further work.

### Recommendations

Veterinary nurses should continue to strive for a salary reflective of their qualifications and skills. The representative body for veterinary nurses should work with the veterinary representative bodies and corporate veterinary groups to develop professional salary banding based on clear responsibilities and accountabilities at each level as well as clearly signposted career pathway progression routes aligned with the salary bands, using the NHS as a starting point for the framework. This would provide a clear view of roles linked to salaries and signpost the requirements to move from one band and salary point to the next.

Unfortunately, within the RCVS surveys of 2014 and 2019, the term ‘job satisfaction' was not defined. Castaneda and Scanlan (10) concluded that if autonomous practise, good working relationships, and patient care were all present, then so was job satisfaction. These findings were corroborated by Hayes et al. (17), who determined that there were 44 factors contributing to job satisfaction, categorised into intra-, inter-, and extra-personal factors. Included within the intra-personal factors were the individuals' own coping strategies to be able to reframe their own perspective in terms of the workplace and their own job satisfaction [(17), p. 808].

A recommendation to the RCVS is that if a question regarding job satisfaction is included in any future surveys, a clear definition is provided such as that by Castaneda and Scanlan (10) who determine job satisfaction being present when there are autonomous practise, good working relationships, and patient care. This prevents the term “job satisfaction” being used as an umbrella term meaning different things to different individuals and, therefore, resulting in data that are unable to be interrogated more fully.

Finally, to gain a more accurate analysis of survey findings, the RCVS may wish to request multivariable logistic regression modelling of the data by the IES to ensure that the data gathered from the large-scale surveys of the professions which they undertake are as valuable as they can be. This is important, as it can then provide a clear evidence base for strategic planning in future by the regulatory body concerning veterinary nursing.

### Possible limitations

In terms of providing previously published veterinary nursing research to inform the subject, this was limited to the previous RCVS surveys of the veterinary nursing profession and one other veterinary nursing paper. The main body of the literature, therefore, related to human-centred nursing and a small number related to dental nursing.

One other limitation may have been selection bias related to the way in which respondents answered the questions within the survey. For example, if respondents were particularly unhappy about their career, they may have been more motivated to fill in the entire survey, so could be over-represented in the final sample within the multivariable analysis.

Another possible weakness was that the predominant focus of the literature as well as within the original surveys was on the intention to leave rather than on those who had already left the profession, be it veterinary nursing, veterinary medicine, human-centred, or dental nursing (17).

## Data availability statement

The original contributions presented in the study are included in the article/Supplementary material, further inquiries can be directed to the corresponding author.

## Ethics statement

The studies involving human participants were reviewed and approved by University of Bristol, Faculty of Social Sciences and Law, Graduate School of Education. The patients/participants provided their written informed consent to participate in this study.

## Author contributions

ET completed the statistical analysis of the 2019 Survey data. Both authors contributed to the article and approved the submitted version.

## Funding

Linnaeus Veterinary Limited supported the costs of the Open Access Publication Charges.

## Conflict of interest

The authors declare that the research was conducted in the absence of any commercial or financial relationships that could be construed as a potential conflict of interest.

## Publisher's note

All claims expressed in this article are solely those of the authors and do not necessarily represent those of their affiliated organizations, or those of the publisher, the editors and the reviewers. Any product that may be evaluated in this article, or claim that may be made by its manufacturer, is not guaranteed or endorsed by the publisher.
