# Supplementary material for: Veterinary nursing in the United Kingdom: Identifying the factors that influence retention within the profession
Source: Front Vet Sci. 2022 Nov 23;9:927499. doi: 10.3389/fvets.2022.927499 (PMC9728522; doi:10.3389/fvets.2022.927499)
Supplement: Supplementary file 1 [file Data_Sheet_1.docx]

**Supplementary Material (1)**

Figure 1. RVN removals, restorations and registrations 2008 - 2021

Table 1. Survey questions including subject area, full wording, possible responses ,coding undertaken and risk ratio (RR)

| **Survey item** | **Subject area** | **Full question wording** | **Responses** | **Variable name** | **Coding to variable** | **Value labels** | **Comparison with 2014 variable** | **Full sample relative risk (RR)** | |
| --- | --- | --- | --- | --- | --- | --- | --- | --- | --- |
|  |  |  |  |  |  |  |  | **2014** | **2019** |
| A4 (2019)  A2 (2014) | Age | What is your age? | Age in years | Age | Coded <16yrs and >100yrs as missing | No labels | No change to question wording | n/a | n/a |
| A16 (2019)  A11 (2014) | Primary qualification | Which of the following is your primary veterinary nurse qualification? | 1. Degree 2. Foundation degree 3. Level 3 Diploma 4. NVQ 5. Certificate (pre-2000) 6. Other | Higher_  ed | 3,4,5 = 0  1,2 = 1  6 = NA | 0 = Further education  1 = Higher education | Change to question wording.  2014 wording: “Is the primary VN qualification that you have, or for which you are currently studying a …” This question wording change was due to student VNs not being included in the survey in 2019. | 1.555 | 0.986 |
| C9 (2019)  C7 (2014) | Second job | Do you have more than one job (including voluntary / unpaid work), regardless of whether your additional jobs are within or outside the veterinary nursing profession? | 1. Yes 2. No | Second_  job | 2 = 0  1 = 1  NA = NA | 0 = doesn't have second job  1 = has second job | Change to question wording, clarifying further what is meant by ‘second job’. (Note similar proportion said ‘yes’ in both years).  2014 wording: “Do you have a second job?” | 0.783 | 0.708 |
| F3-b (2019)  F1-b (2014) | Undertake nurse clinics | Please indicate how often you undertake the following clinical and non-clinical work.  * Nursing clinics / counselling | 1. Every day 2. Several times per week 3. Once per week or less, but at least once a month 4. Rarely, ie less than once a month 5. Never | Nurse_  clinics | 3,4,5 = 0  1,2 = 1  NA = NA | 0 = once a week or less  1= more than once a week | No change to question wording  (“Nursing clinics/counselling”). Slight wording change to question intro, and answer options, but meaning is retained.  2014 wording:  “Please indicate which of the following clinical and non-clinical work you do in practice, and how often (please tick one box in each row)  1. Every day  2. Several times a week  3. Once per week or less  4. Rarely  5. Never” | 1.010 | 0.832 |
| F3-r (2019)  F1-j (2014) | Undertake minor surgical procedures | Please indicate how often you undertake the following clinical and non-clinical work.  * Minor surgery not entering the body  cavity | 1. Every day 2. Several times per week 3. Once per week or less, but at least once a month 4. Rarely, ie less than once a month 5. Never | Nurse_  clinics2 | 3,4,5 = 0  1,2 = 1  NA = NA | 0 = once a week or less  1= more than once a week | Statement changed from “performing minor surgical procedures”.  Moved position in list of statements.  Slight wording change to question intro and answer options, but meaning is retained. (see row above) | 0.975 | 0.994 |
| I1-9 (2019)  J1-11 (2014) | Valued by VS | * Veterinary nurses are valued by veterinary surgeons. | 1. Strongly disagree 2. Disagree 3. Neither agree nor disagree 4. Agree 5. Strongly agree | Valued | 1,2=0  4,5=1  3,NA=NA | 0 = Disagree  1 = Agree | Slight wording change (spelling out VN).  2014 wording:  “VNs are valued by veterinary surgeons” | 2.959 | 2.307 |
| I1-14 (2019)  J1-14 (2019) | Career progression | *Veterinary nursing offers good opportunities for career progression. | 1. Strongly disagree 2. Disagree 3. Neither agree nor disagree 4. Agree 5. Strongly agree | Career_  prog | 1,2=0  4,5=1  3,NA=NA | 0 = Disagree  1 = Agree | No change to question wording | 5.100 | 3.666 |
| I1-15 (2019  J1-15 (2014) | Support by employer | * (For employed veterinary nurses) I am satisfied with the support given by my employer. | 1. Strongly disagree 2. Disagree 3. Neither agree nor disagree 4. Agree 5. Strongly agree | Employer_sup | 1,2=0  4,5=1  3,NA=NA | 0 = Disagree  1 = Agree | No change to question wording | 3.697 | 2.744 |
| I1-17 (2019)  J1-17 (2014) | Salary satisfaction | * I am satisfied with my salary /  remuneration level. | 1. Strongly disagree 2. Disagree 3. Neither agree nor disagree 4. Agree 5. Strongly agree | Salary_  sat | 1,2=0  4,5=1  3,NA=NA | 0 = Disagree  1 = Agree | Question wording change.  2014 wording:  “I am satisfied with my salary level”. | 2.412 | 3.146 |
| I5-2 (2019)  J5-2 (2014) | Client expectations and demands | In your opinion, what are the main challenges currently facing the veterinary nursing profession (please select up to three of the following, which have been derived from previous RCVS surveys)  * Client expectations / demands | Participant required to tick box | Client_  challenge | 1 = 1  0 = 0  8 = NA | 0 = Disagree  1 = Agree | Small change to question wording (stating that the answer options have been derived from previous RCVS surveys).  Some answer options have been added, but these appear after ‘client expectations’ in the list.  2014 question wording.  “In your opinion, what are the main challenges currently facing the veterinary nursing profession (please select up to three)” | 1.310 | 1.193 |
| I5-9 (2019)  J5-7 (2014) | Lack of respect/recognition - employers | * Lack of respect / recognition from employers | Participant required to tick box | Lack_of_  respect_  emp | 0 = 0  1 = 1  NA = NA | 0 = feel there isn't a lack of respect  1 = feel there is a lack of respect | Question change. In 2014 there was only one question asking about “Lack of respect/recognition for the profession from employers/vets”.  Also note that some answer options preceding these are new since 2014.  The data shows a notable difference between people selecting this option in 2019 and the equivalent option in 2014. | 0.508 | 0.666 |
| I5-10 (2019)  J5-7 (2014) | Lack of respect/recognition -vets | * Lack of respect / recognition from veterinary surgeons | Participant required to tick box | Lack_of_  respect_vs | 0 = 0  1 = 1  NA = NA | 0 = feel there isn't a lack of respect  1 = feel there is a lack of respect | See “lack of respect/recognition – employers” above. | 0.508 | 0.724 |
| G3-1(2019)  G1 (2014) | Time off for CPD | Do you receive any of the following benefits at work? (Please select all that apply.)  * Time off (paid) for training / CPD | Participant required to tick box | cpd_time  off | 0 = 0  1 = 1  NA = NA | 0 "no paid time off for CPD"  1 "receive paid time off for CPD". | Not asked in 2014. | n/a | 1.364 |
| I5-6 (2019)  J1-6 (2014) | Stress | In your opinion, what are the main challenges currently facing the veterinary nursing profession (please select up to three of the following, which have been derived from previous RCVS surveys)  * Stress levels | Participant required to tick box | Stress_  challenge | 0 = 0  1 = 1  NA = NA | 0 “stress not a main challenge for VNs”  1 “stress is a main challenge for VNs” | Not asked in 2014. | n/a | 1.019 |

Table 2. The descriptive statistics for both the 2014 full sample and complete case sample, in total and broken down by the

values of the dependent variable

| Question | Response | Full sample (n =4586) | | | Complete case sample (n = 1058) | | |
| --- | --- | --- | --- | --- | --- | --- | --- |
|  |  | Stay in VN | Leave VN | Total | Stay in VN | Leave VN | Total |
| Feels there is a lack of respect | Selected  Not selected  Missing | 890 (76%)  2170 (88%)  932 (20%) | 287 (25%)  307 (12%) | 1177 (32%)  2477 (68%) | 221 (70%)  654 (88%) | 94 (30%)  89 (12%) | 315 (30%)  743 (70%) |
| Has second job | Selected  Not selected  Missing | 606 (81%)  3244 (85%)  18 (0.4%) | 144 (19%)  574 (15%) | 750 (16%)  3818(84%) | 142 (75%)  733 (84.3%) | 46(25%)  137 (16%) | 188 (18%)  870 (82%) |
| Undertakes nurse clinics regularly | Selected  Not selected  Missing | 2008 (84%)  1483 (85%)  441 (9.6%) | 381 (16%)  273 (15%) | 2389 (58%)  1756 (42%) | 526 (85.3%)  265 (77.5%)  99 (9.4%) | 91 (14.7%)  77 (22.5%) | 617 (64.3%)  342 (35.7%) |
| Performs minor surgical procedures | Selected  Not selected  Missing | 268 (86%)  3229 (84%)  439 (9.6%) | 43 (14%)  607 (16%) | 311 (7.5%)  3836 (92.5%) | 66 (84.6%)  720 (82.5%)  107(10.1%) | 12 (15.4%)  153 (17.5%) | 78 (8.2%)  873 (91.8%) |
| Any days CPD | Selected  Not selected  Missing | 1527 (82%)  615 (78%)  1934 (42%) | 341 (18%)  171 (22%) | 1868 (70%)  786 (30%) | 642 (84%)  233 (80%) | 124 (16%)  59 (20%) | 766 (72%)  292 (28%) |
| Client expectations and demands are a challenge | Selected  Not selected  Missing | 1036 (86%)  2024 (82%)  932 (20%) | 161 (14%)  433 (18%) | 1197 (33%)  2457 (67%) | 371 (91%)  504 (77%) | 36 (9%)  147 (23%) | 407 (38%)  651 (62%) |
| Higher education qualification | Yes  No  Missing | 713 (89%)  3022 (83%)  149 (3%) | 87 (11%)  615 (17%) | 800 (18%)  3637 (82%) | 159 (86%)  716 (82%) | 26 (14%)  157 (18%) | 185 (17%)  873 (83%) |
| Salary Satisfaction | Selected  Not selected  Missing | 830 (91%)  1639 (79%)  1596 (35%) | 80 (9%)  441 (21%) | 910 (30%)  2080 (70%) | 371 (91%)  504 (77%) | 36 (8.8%)  147 (23%) | 407 (38%)  651 (62%) |
| Valued by vet | Selected  Not selected  Missing | 1794 (90%)  624 (71%)  1710 (37%) | 198 (10%)  260 (29%) | 1992 (69%)  884 (31%) | 666 (89%)  209 (67%) | 81 (11%)  102 (33%) | 747 (71%)  311 (29%) |
| Satisfied with employer support | Selected  Not selected  Missing | 2006 (91%)  476 (66%)  1664 (36%) | 200 (9%)  240 (34%) | 2206 (75%)  716 (25%) | 697 (89%)  178 (65%) | 86 (11%)  97 (35%) | 783 (74%)  275 (26%) |
| Good opportunity for career progression | Selected  Not selected  Missing | 1690 (94%)  813 (70%)  1616 (35%) | 108 (6%)  359 (31%) | 1798(60%)  1172 (40%) | 532 (92%)  343 (71%) | 46 (8%)  137 (29%) | 578 (55%)  480 (45%) |
| VN gives me job satisfaction | Selected  Not selected  Missing | 2963 (88%)  55 (37%)  1089 (23.7%) | 385 (12%)  94 (63%) | 3348 (96%)  149 (4%) | 850 (86%)  25 (34%) | 134 (14%)  49 (66%) | 984 (93%)  74 (7%) |
| Age | N  Mean  (SD)Missing | 3840 (84.3%)30.2 (8.8%)  22 (0.6%) | 715(15.7%)  31.6 (7.3%)  9 (1.3%) | 4555 (100%)  30.4 (8.6%)  31 (0.7%) | 875 (82.7%)  33.0 (8.0%) | 183 (17.3%)  33.1 (6.7%) | 1058 (100%)  33.0 (7.8%) |

Notes: ^a^ Percentages of those intending to stay in/leave the profession, by levels of the predictor variable (sum horizontally to 100%).

^b^ Percentage of total sample at each level of the predictor variable (sum vertically to 100%).

Table 3. The descriptive statistics for both the 2019 full sample and complete case sample, in total and broken down by the

values of the dependent variable

| Question | Response | Full sample (n = 6369) | | | | | | Complete case sample (n=1731) | | | | | |
| --- | --- | --- | --- | --- | --- | --- | --- | --- | --- | --- | --- | --- | --- |
|  |  | Stay in VN | | Leave VN | | Total | | Stay in VN | | Leave VN | | Total | |
|  |  | N | % | N | % | N | % | N | % | N | % | N | % |
| Feels there is a lack of respect | Selected | 583 | 15.4% | 304 | 24.3% | 887 | 18% | 167 | 12.2% | 90 | 24.6% | 257 | 14.8% |
|  | Not selected | 3206 | 84.6% | 949 | 75.7% | 4155 | 82% | 1198 | 87.8% | 276 | 75.4% | 1474 | 85.2% |
|  | Missing | 1327 | 21% |  |  |  |  | 0 | 0.0% |  |  |  |  |
|  |  | 416 | 11.0% | 203 | 16.2% | 619 | 12% | 131 | 9.6% | 58 | 15.8% | 189 | 10.9% |
|  |  | 3373 | 89.0% | 1050 | 83.8% | 4423 | 88% | 1234 | 90.4% | 308 | 84.2% | 1542 | 89.1% |
|  |  | 1327 | 21% |  |  |  |  | 0 | 0.0% |  |  |  |  |
| Has second job | Selected | 684 | 14.7% | 347 | 21.8% | 1031 | 16% | 173 | 12.7% | 75 | 20.5% | 248 | 14.3% |
|  | Not selected | 3974 | 85.3% | 1244 | 78.2% | 5218 | 84% | 1192 | 87.3% | 291 | 79.5% | 1483 | 85.7% |
|  | Missing | 6369 | 100% |  |  |  |  | 0 | 0.0% |  |  |  |  |
| Undertakes nurse clinics regularly | Selected | 2045 | 59.9% | 710 | 60.1% | 2755 | 60% | 620 | 59.8% | 177 | 64.6% | 797 | 60.8% |
|  | Not selected | 1367 | 40.1% | 471 | 39.9% | 1838 | 40% | 417 | 40.2% | 97 | 35.4% | 514 | 39.2% |
|  | Missing | 1776 | 28% |  |  |  |  | 420 | 24.3% |  |  |  |  |
| Performs minor surgical procedures | Selected | 177 | 5.2% | 78 | 6.6% | 255 | 6% | 37 | 3.6% | 18 | 6.6% | 55 | 4.2% |
|  | Not selected | 3234 | 94.8% | 1104 | 93.4% | 4338 | 94% | 999 | 96.4% | 255 | 93.4% | 1254 | 95.8% |
|  | Missing | 1776 | 28% |  |  |  |  | 422 | 24.4% |  |  |  |  |
| Any days CPD | Selected | 3122 | 79.6% | 904 | 71.9% | 4026 | 78% | 1107 | 81.1% | 258 | 70.5% | 1365 | 78.9% |
|  | Not selected | 802 | 20.4% | 354 | 28.1% | 1156 | 22% | 258 | 18.9% | 108 | 29.5% | 366 | 21.1% |
|  | Missing | 1187 | 19% |  |  |  |  | 0 | 0.0% |  |  |  |  |
| Client expectations and demands are a challenge | Selected | 1373 | 36.2% | 389 | 31.0% | 1762 | 35% | 517 | 37.9% | 105 | 28.7% | 622 | 35.9% |
|  | Not selected | 2416 | 63.8% | 864 | 69.0% | 3280 | 65% | 848 | 62.1% | 261 | 71.3% | 1109 | 64.1% |
|  | Missing | 1327 | 21% |  |  |  |  | 0 | 0.0% |  |  |  |  |
| Higher education qualification | Selected | 843 | 18.2% | 297 | 18.5% | 1140 | 18% | 261 | 19.5% | 86 | 23.8% | 347 | 20.4% |
|  | Not selected | 3783 | 81.8% | 1307 | 81.5% | 5090 | 82% | 1078 | 80.5% | 276 | 76.2% | 1354 | 79.6% |
|  | Missing | 139 | 2% |  |  |  |  | 1731 | 100.0% |  |  |  |  |
| Salary Satisfaction | Selected | 1246 | 39.6% | 147 | 13.5% | 1393 | 33% | 602 | 44.1% | 53 | 14.5% | 655 | 37.8% |
|  | Not selected | 1897 | 60.4% | 943 | 86.5% | 2840 | 67% | 763 | 55.9% | 313 | 85.5% | 1076 | 62.2% |
|  | Missing | 2136 | 34% |  |  |  |  | 0 | 0.0% |  |  |  |  |
| Valued by vet | Selected | 2276 | 77.0% | 470 | 51.5% | 2746 | 71% | 1070 | 78.4% | 187 | 51.1% | 1257 | 72.6% |
|  | Not selected | 679 | 23.0% | 443 | 48.5% | 1122 | 29% | 295 | 21.6% | 179 | 48.9% | 474 | 27.4% |
|  | Missing | 2501 | 39% |  |  |  |  | 0 | 0.0% |  |  |  |  |
| Satisfied with employer support | Selected | 2364 | 81.3% | 446 | 51.6% | 2810 | 74% | 1117 | 81.8% | 165 | 45.1% | 1282 | 74.1% |
|  | Not selected | 543 | 18.7% | 419 | 48.4% | 962 | 26% | 248 | 18.2% | 201 | 54.9% | 449 | 25.9% |
|  | Missing | 2597 | 41% |  |  |  |  | 0 | 0.0% |  |  |  |  |
| Good opportunity for career progression | Selected | 1535 | 55.2% | 181 | 18.7% | 1716 | 46% | 800 | 58.6% | 67 | 18.3% | 867 | 50.1% |
|  | Not selected | 1245 | 44.8% | 785 | 81.3% | 2030 | 54% | 565 | 41.4% | 299 | 81.7% | 864 | 49.9% |
|  | Missing | 2623 | 41% |  |  |  |  | 0 | 0.0% |  |  |  |  |
| VN gives me job satisfaction | Selected | 3436 | 96.5% | 736 | 75.8% | 4172 | 92% | 1304 | 95.5% | 249 | 68.0% | 1553 | 89.7% |
|  | Not selected | 124 | 3.5% | 235 | 24.2% | 359 | 8% | 61 | 4.5% | 117 | 32.0% | 178 | 10.3% |
|  | Missing | 1838 | 29% |  |  |  |  | 0 | 0.0% |  |  |  |  |
| Stress is a challenge for VNs | Selected | 1780 | 47.0% | 581 | 46.4% | 2361 | 47% | 624 | 45.7% | 157 | 42.9% | 781 | 45.1% |
|  | Not selected | 2009 | 53.0% | 672 | 53.6% | 2681 | 53% | 741 | 54.3% | 209 | 57.1% | 950 | 54.9% |
|  | Missing | 1327 | 21% |  |  |  |  | 0 | 0% |  |  |  |  |
| Age | Mean | 4677 | 34.76 | 1610 | 33.86 | 6287 | 34.53 | 1365 | 34.27 | 366 | 33.20 | 1731 | 34.05 |
|  | Missing | 82 | 1% |  |  |  |  | 0 | 0% |  |  |  |  |

Notes: ^a^ Percentages of those intending to stay in/leave the profession, by levels of the predictor variable (sum horizontally to 100%). ^b^ Percentage of total sample at each level of the predictor variable (sum vertically to 100%)

Table 4. Unadjusted odds ratios for full and complete samples

| Variable | Unadjusted odds ratios (full sample n= 4586) | | | | | Unadjusted odds ratios (Complete sample n= 1058) | | | |
| --- | --- | --- | --- | --- | --- | --- | --- | --- | --- |
|  | OR | Lower 95% CI | Upper 95% CI | p-value | N | OR | Lower 95% CI | Upper 95% CI | p-value |
| Lack of respect | 2.279 | 1.906 | 2.726 | <0.001*** | 3654 | 3.126 | 2.253 | 4.337 | <0.001*** |
| Has second job | 1.343 | 1.097 | 1.644 | 0.004** | 4568 | 1.733 | 1.186 | 2.533 | 0.004** |
| Undertakes nurse clinics regularly | 1.031 | 0.870 | 1.221 | 0.726 | 4145 | 0.595 | 0.425 | 0.834 | 0.003** |
| Performs minor surgical procedures | 0.852 | 0.675 | 1.076 | 0.178 | 4147 | 0.856 | 0.451 | 1.622 | 0.633 |
| Age | 1.018 | 1.009 | 1.027 | <0.001*** | 4555 | 1.003 | 0.982 | 1.023 | 0.809 |
| Any days of CPD | 0.803 | 0.654 | 0.987 | 0.037** | 2654 | 0.763 | 0.541 | 1.076 | 0.123 |
| Client expectations and demands are a challenge in VN | 0.726 | 0.597 | 0.884 | 0.001** | 3654 | 0.753 | 0.531 | 1.069 | 0.113 |
| Higher education qualification | 0.600 | 0.472 | 0.761 | <0.001*** | 4437 | 0.746 | 0.476 | 1.169 | 0.200 |
| Salary satisfaction | 0.358 | 0.278 | 0.461 | <0.001*** | 2990 | 0.333 | 0.226 | 0.491 | <0.001*** |
| Valued by vet surgeons | 0.265 | 0.216 | 0.326 | <0.001*** | 2876 | 0.249 | 0.179 | 0.347 | <0.001*** |
| Satisfied with employer support | 0.198 | 0.160 | 0.245 | <0.001*** | 2922 | 0.226 | 0.162 | 0.316 | <0.001*** |
| Good opportunity for career progression | 0.145 | 0.115 | 0.182 | <0.001*** | 2970 | 0.216 | 0.151 | 0.310 | <0.001*** |
| VN gives me job satisfaction | 0.076 | 0.054 | 0.108 | <0.001*** | 3497 | 0.080 | 0.048 | 0.135 | <0.001*** |

**
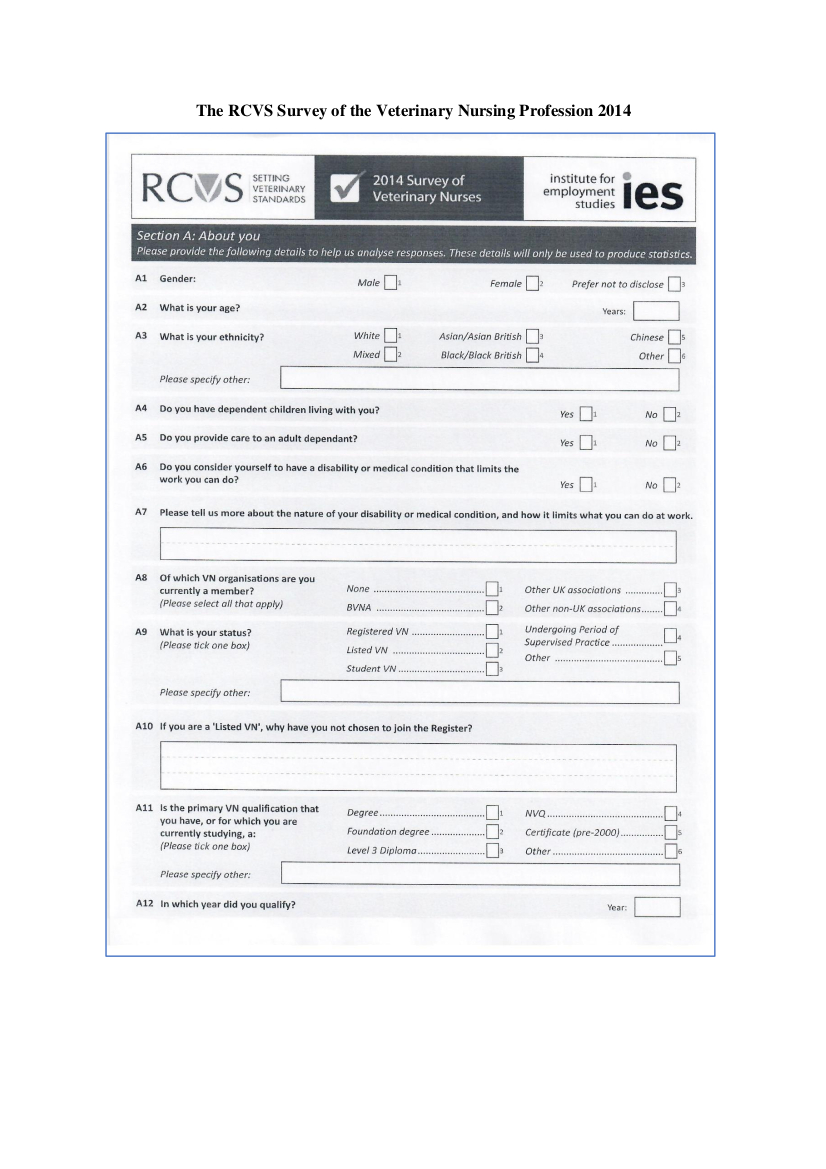
**


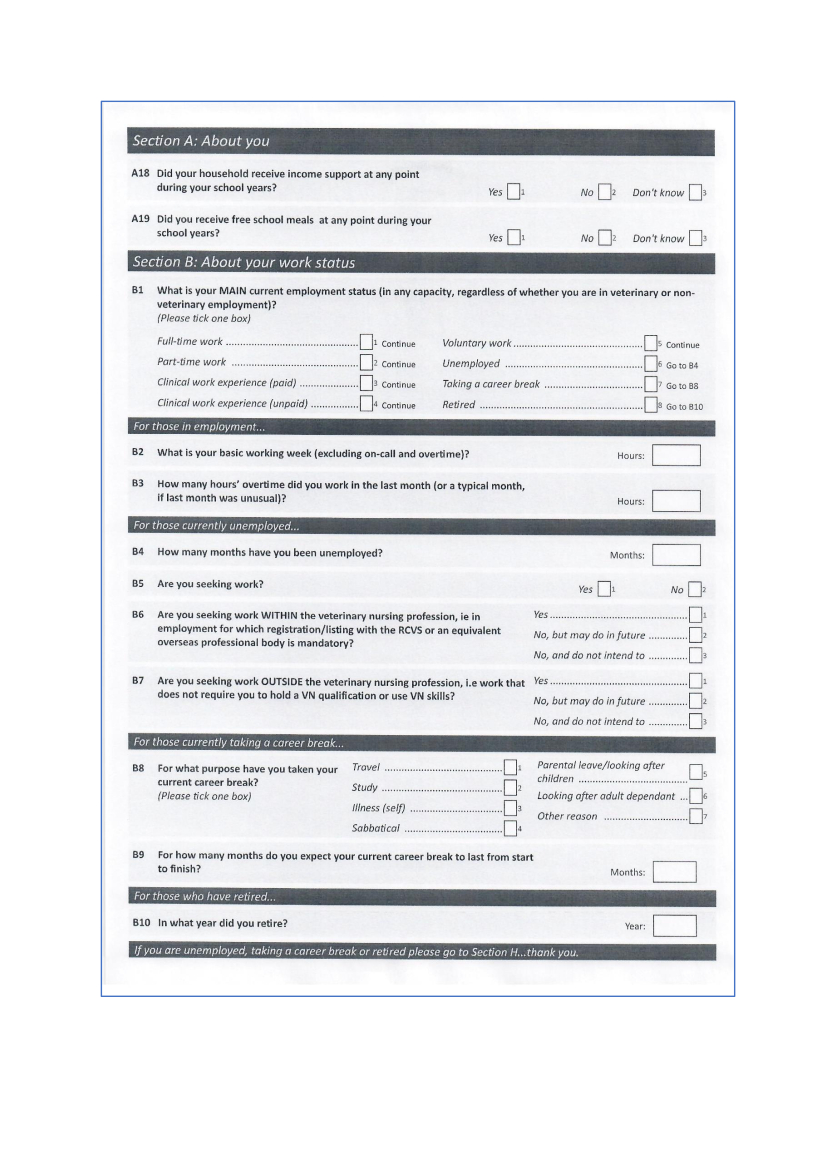


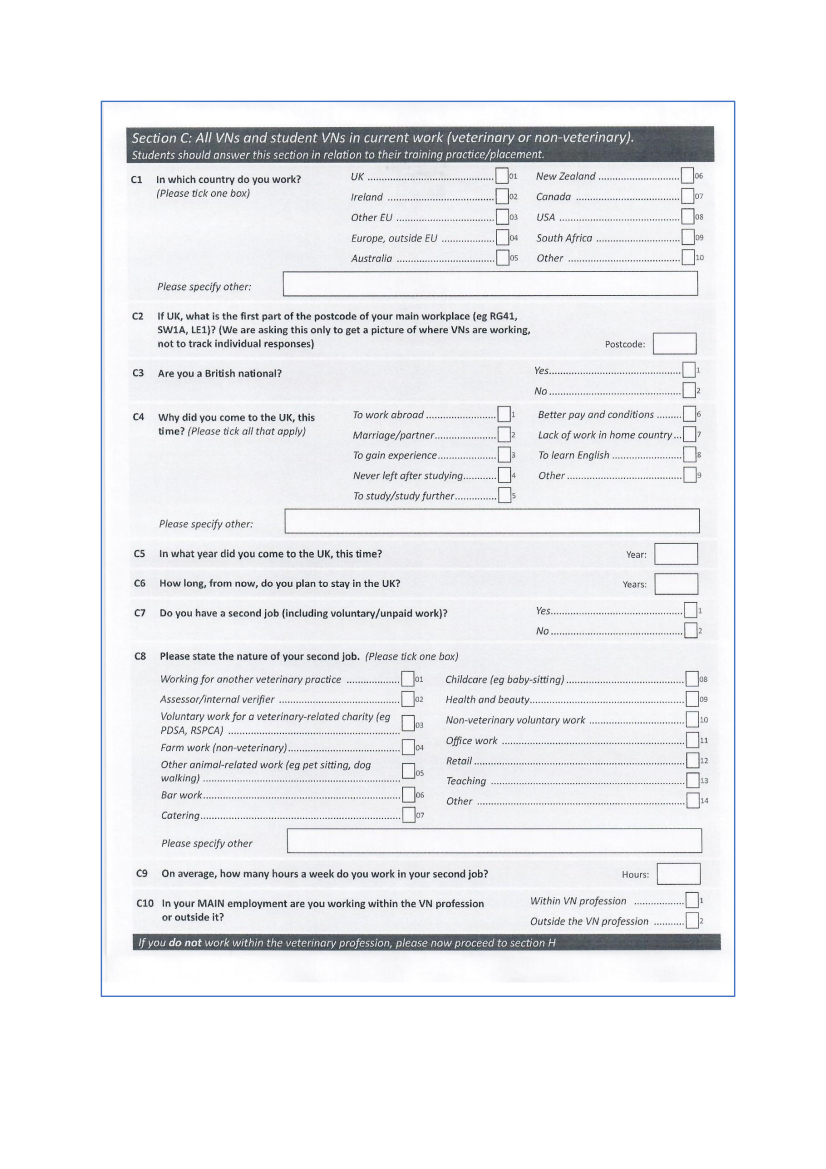


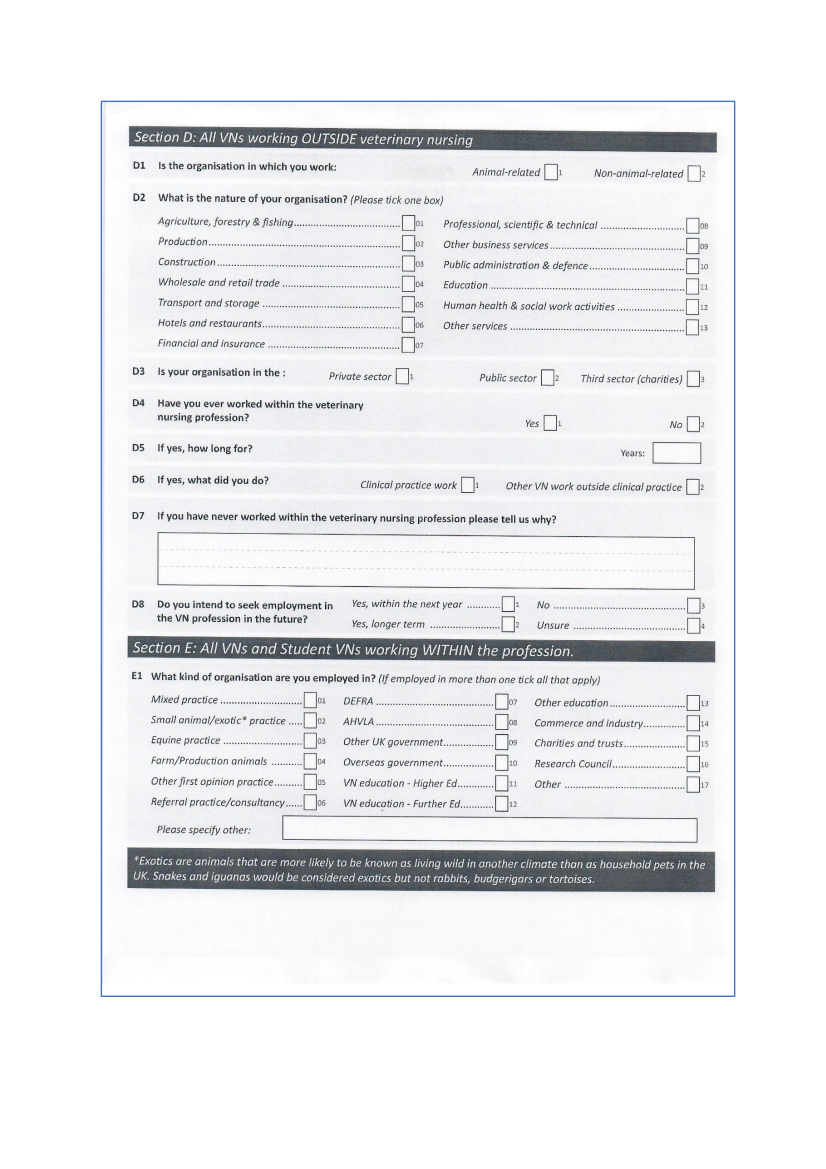


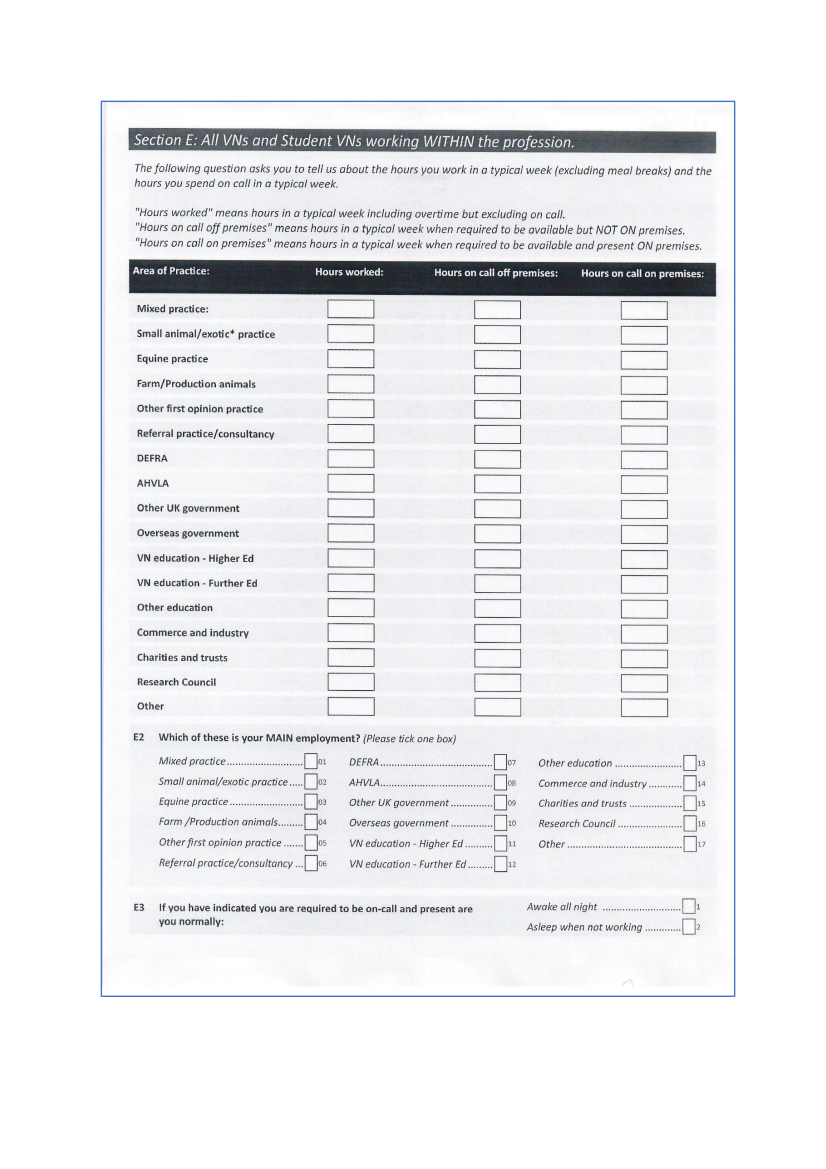


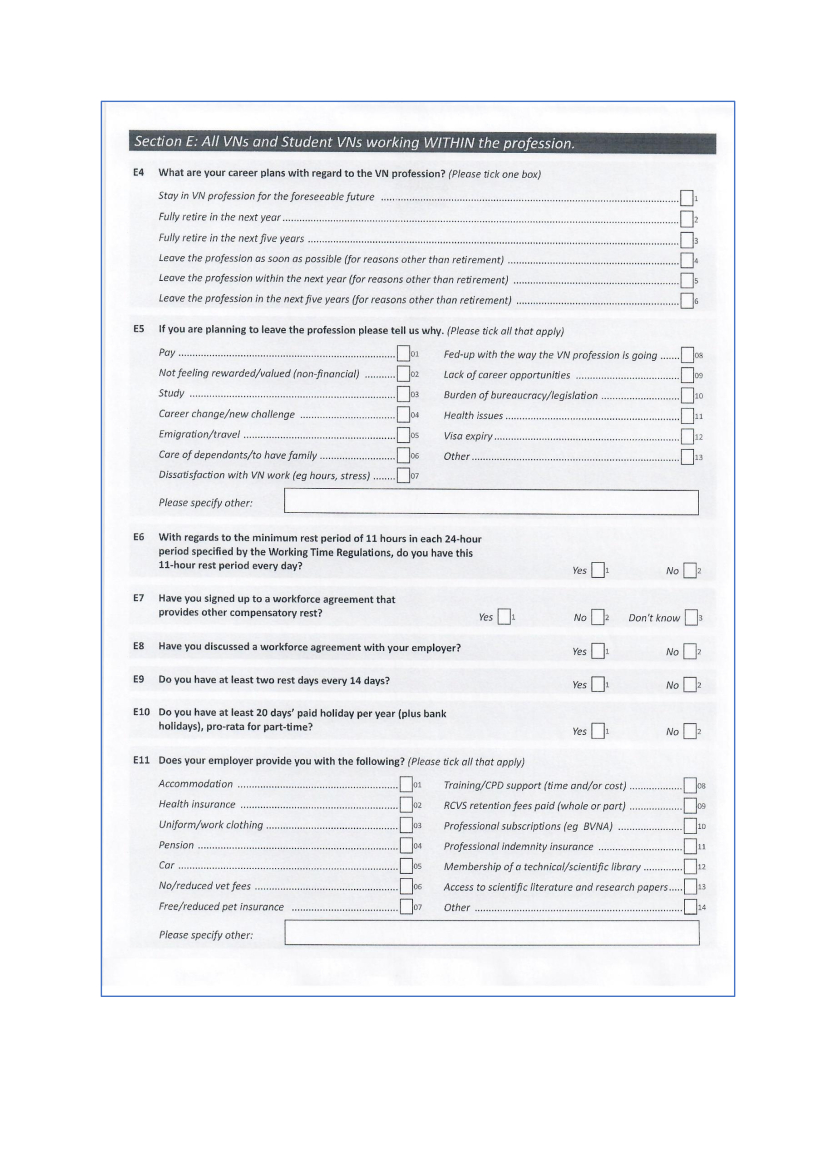


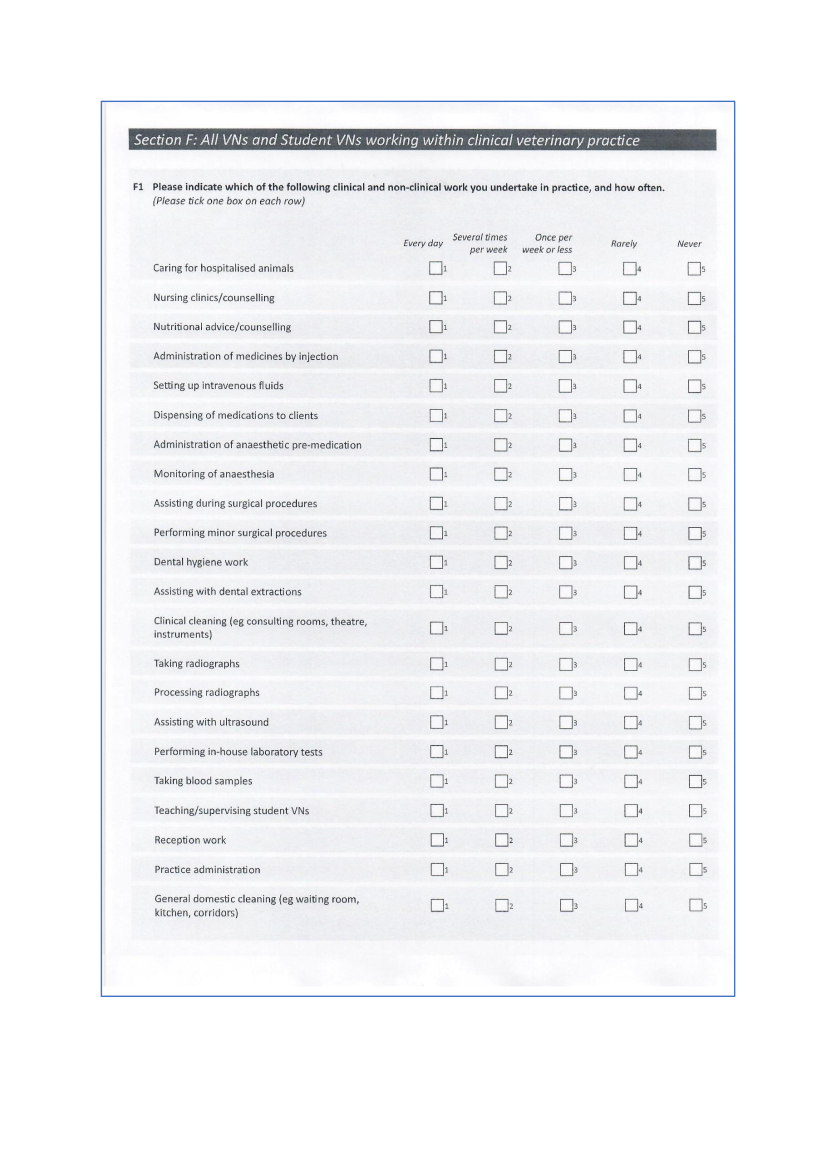


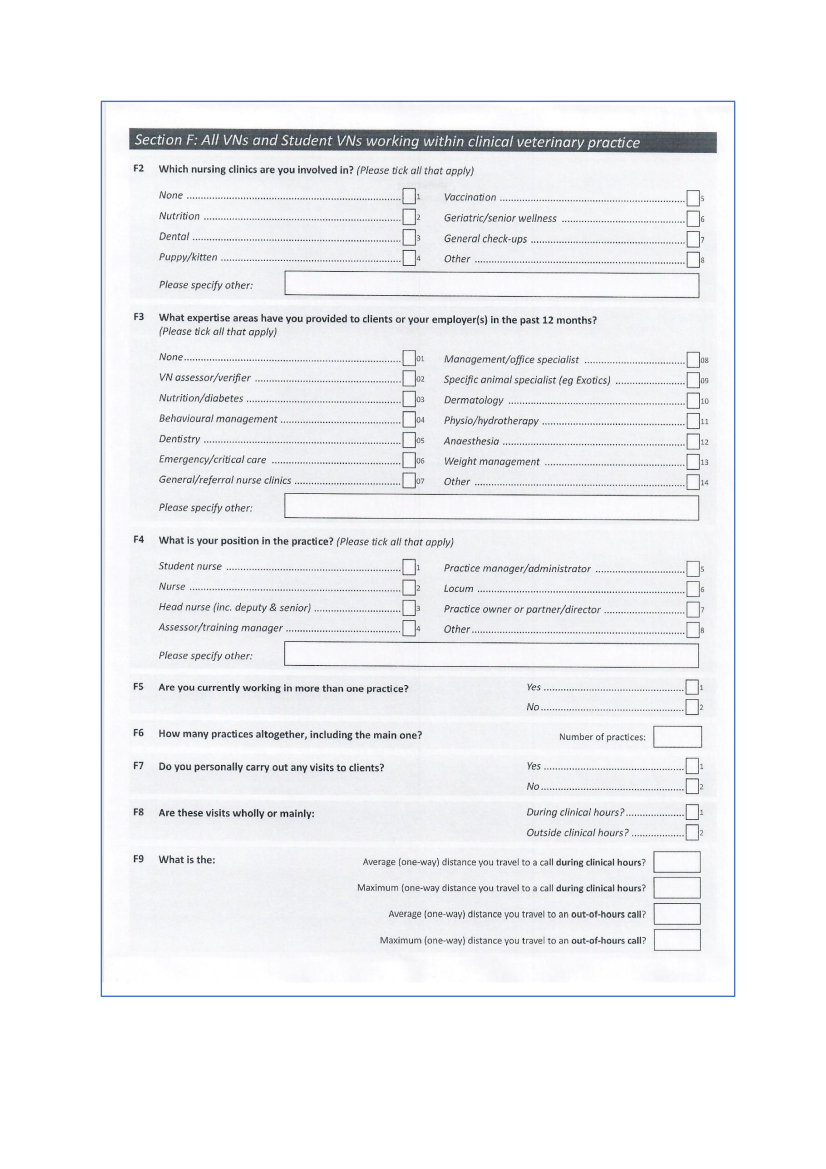


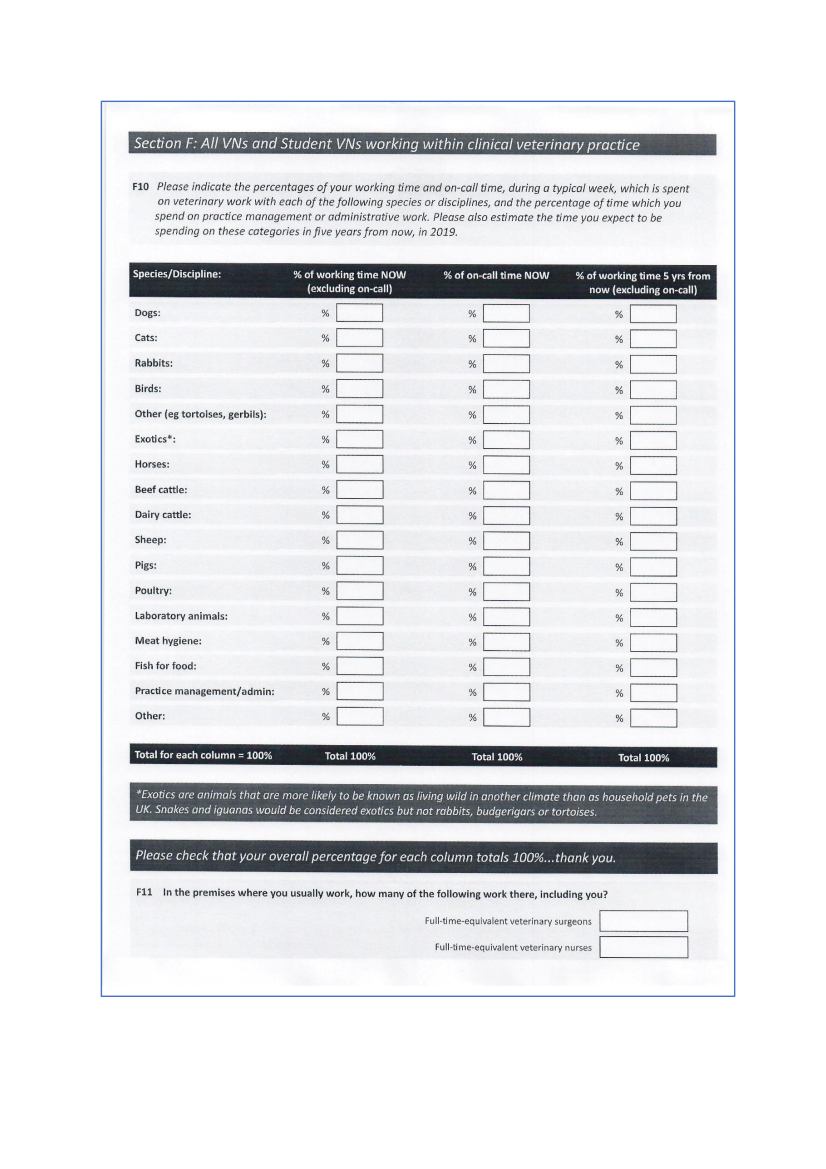


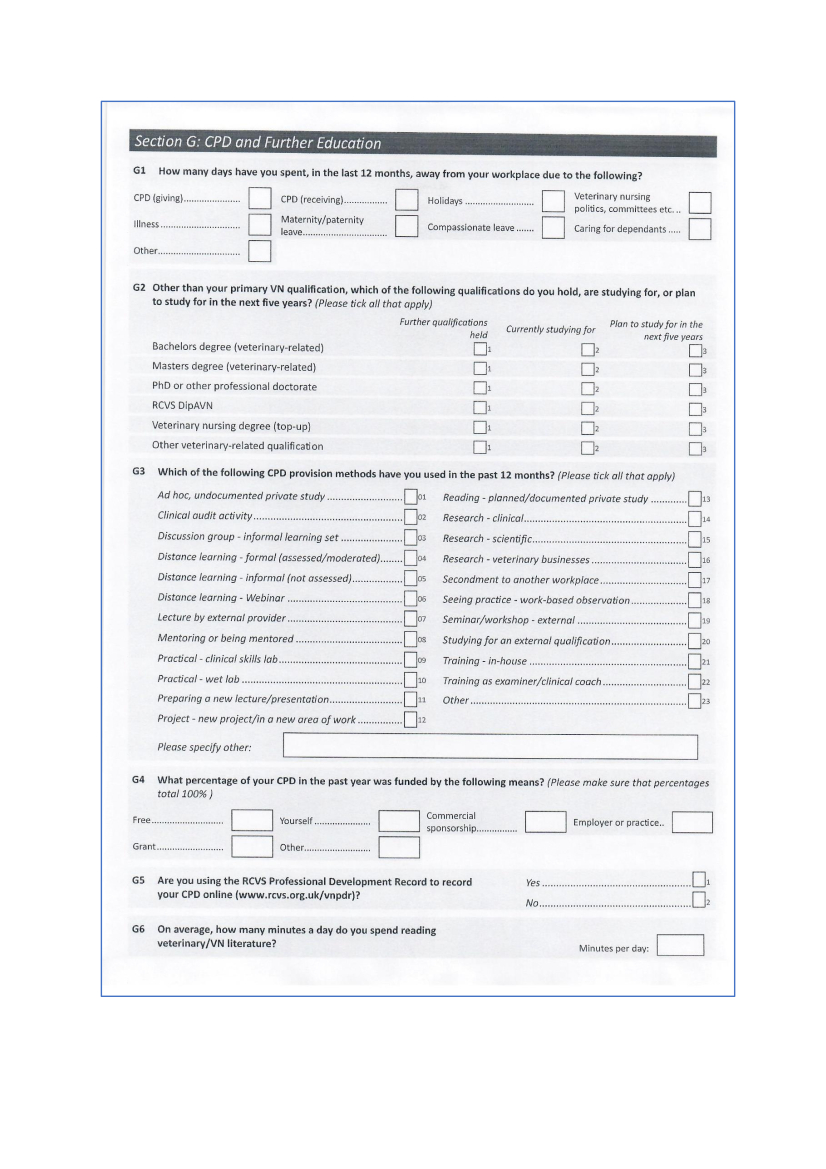


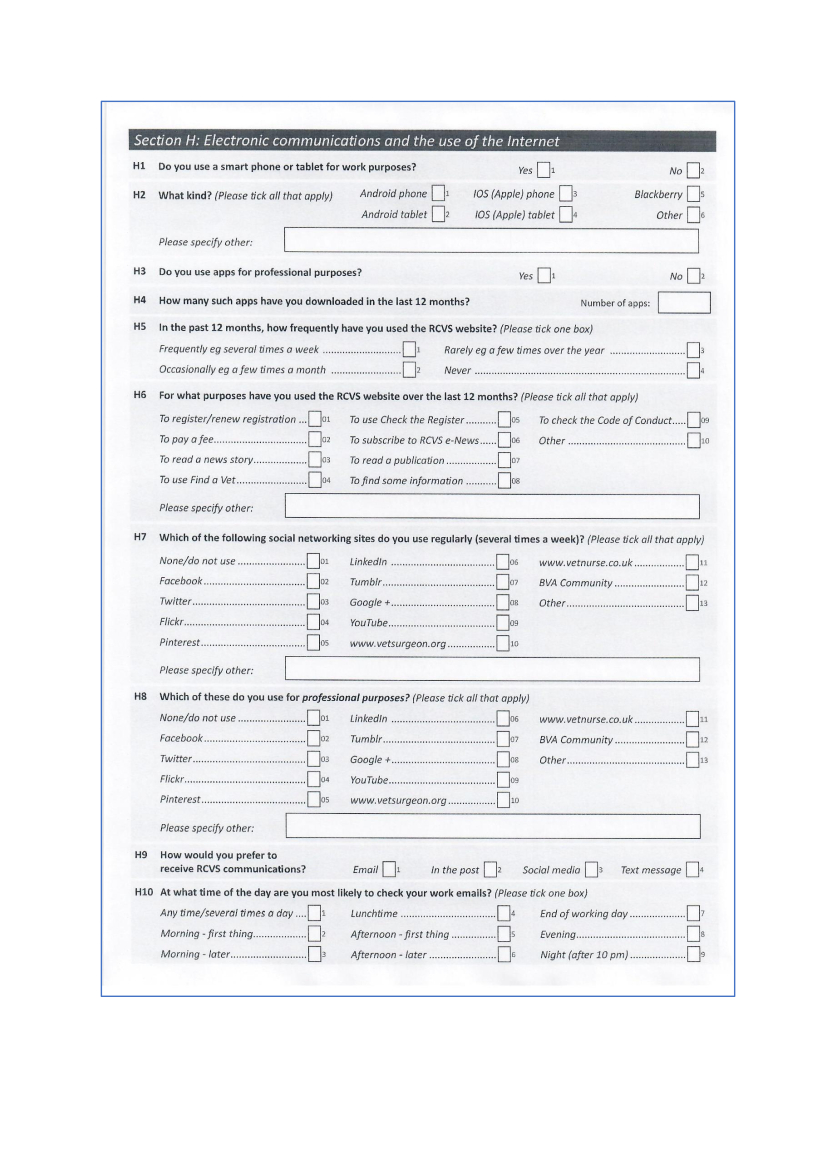


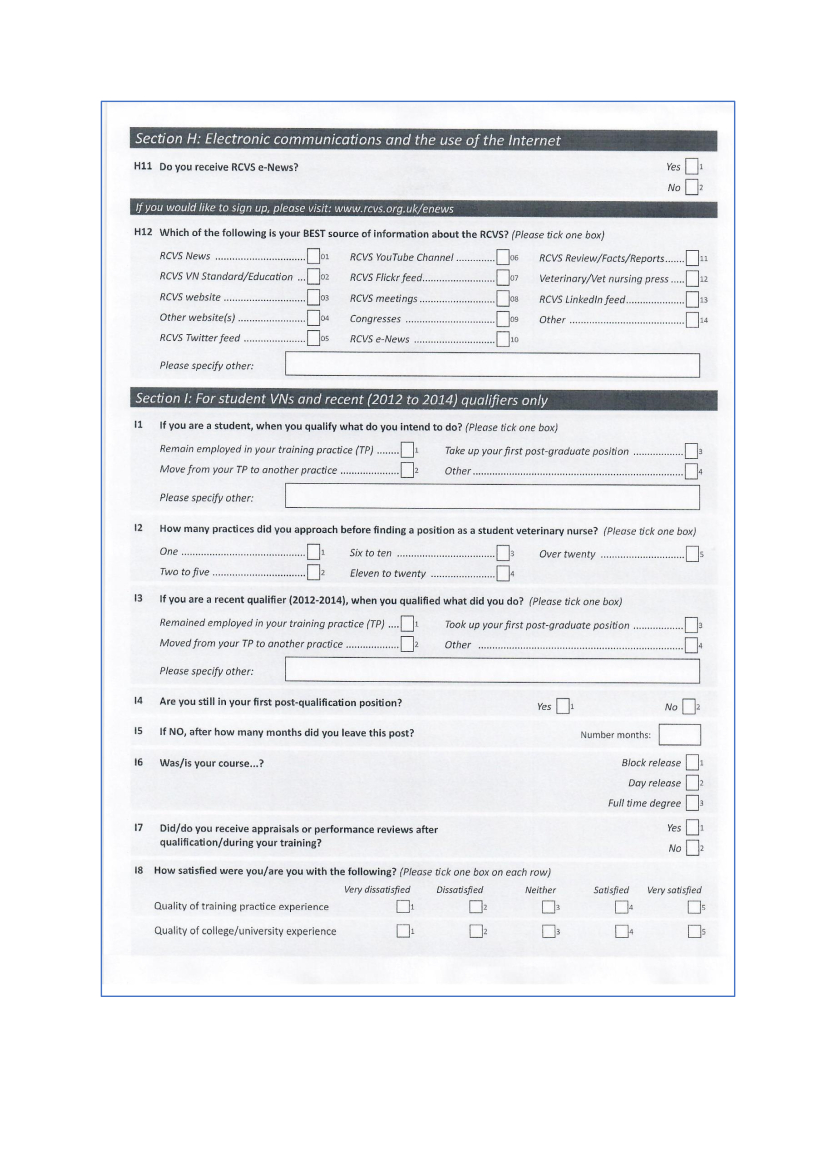


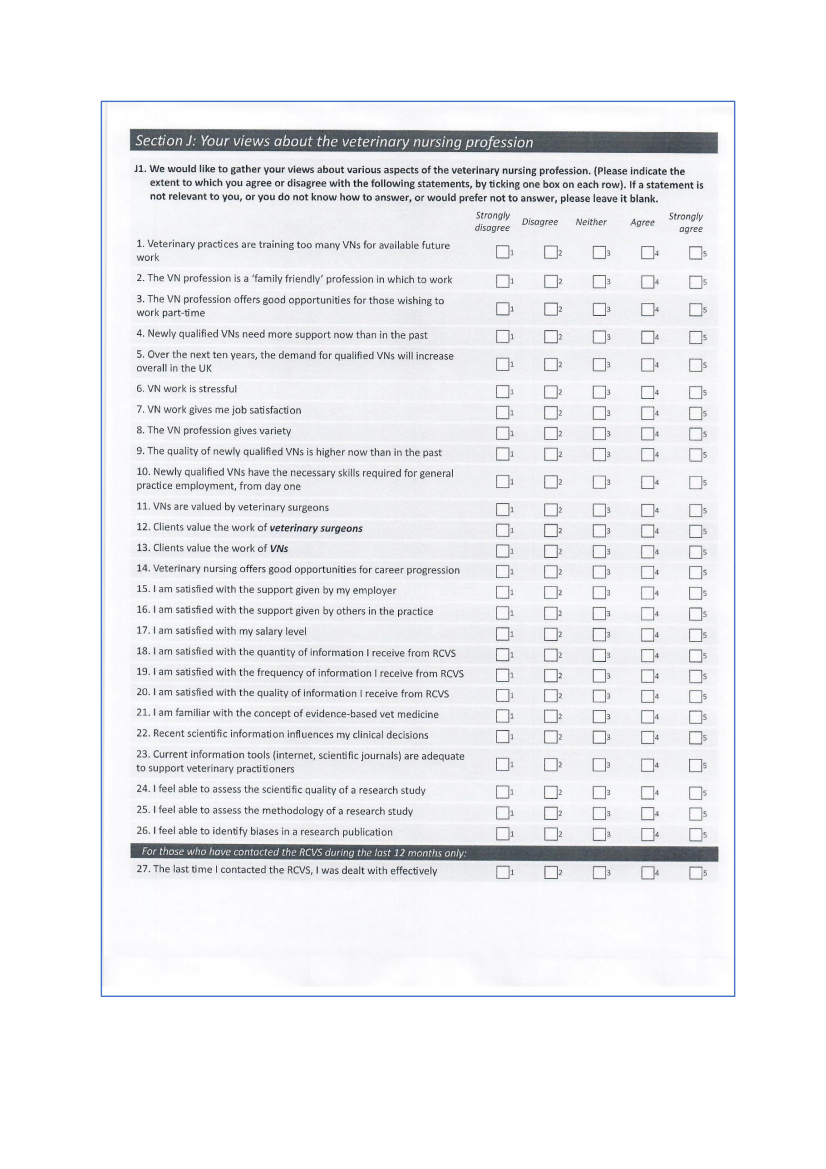


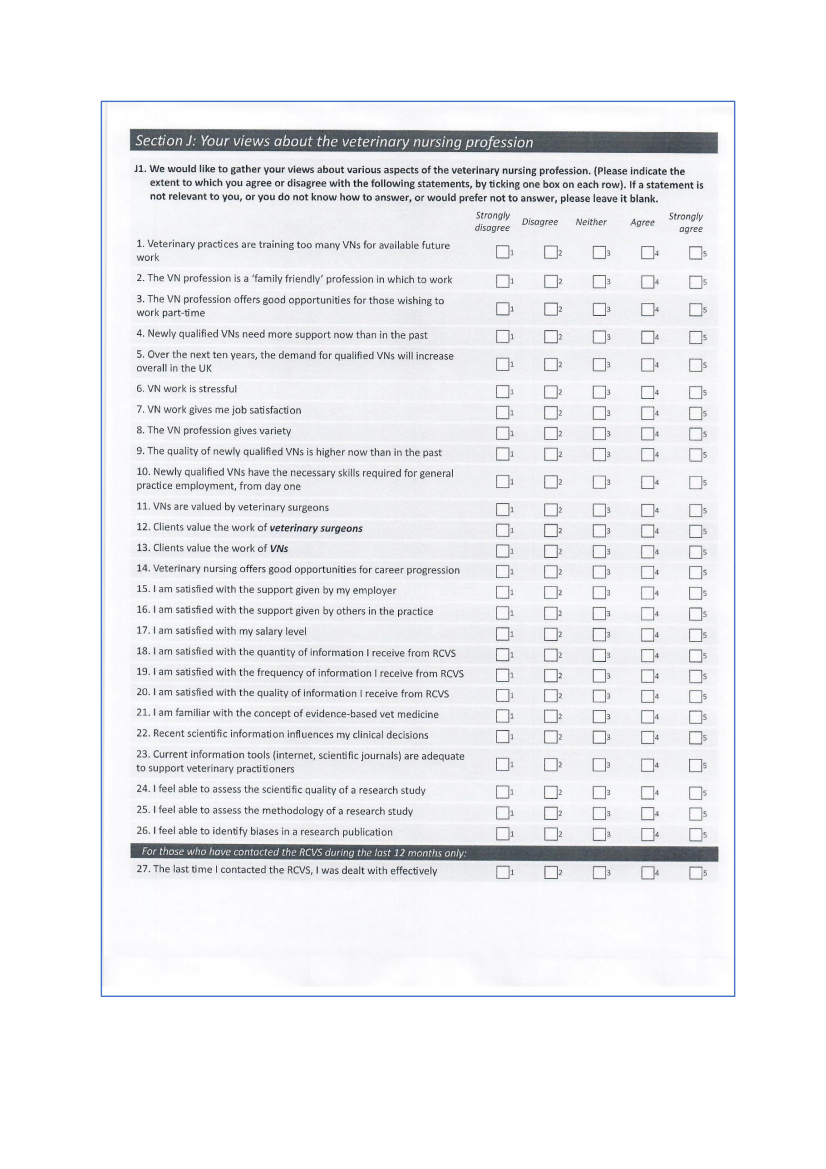


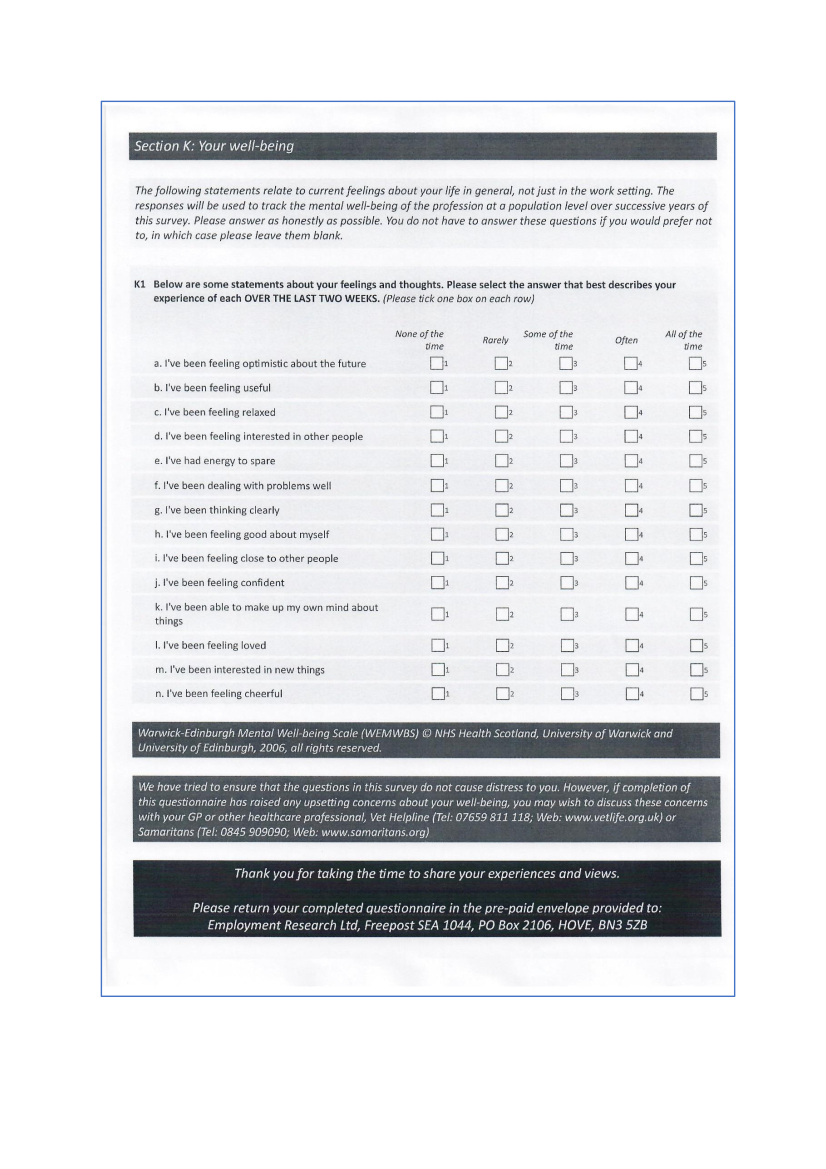


**
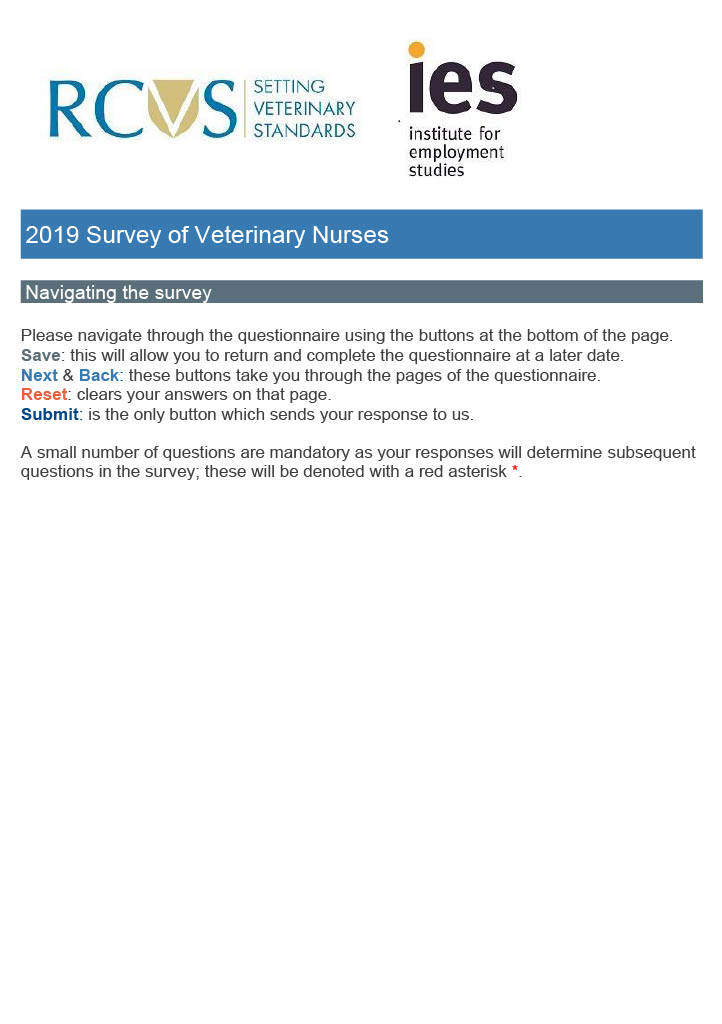
**

**
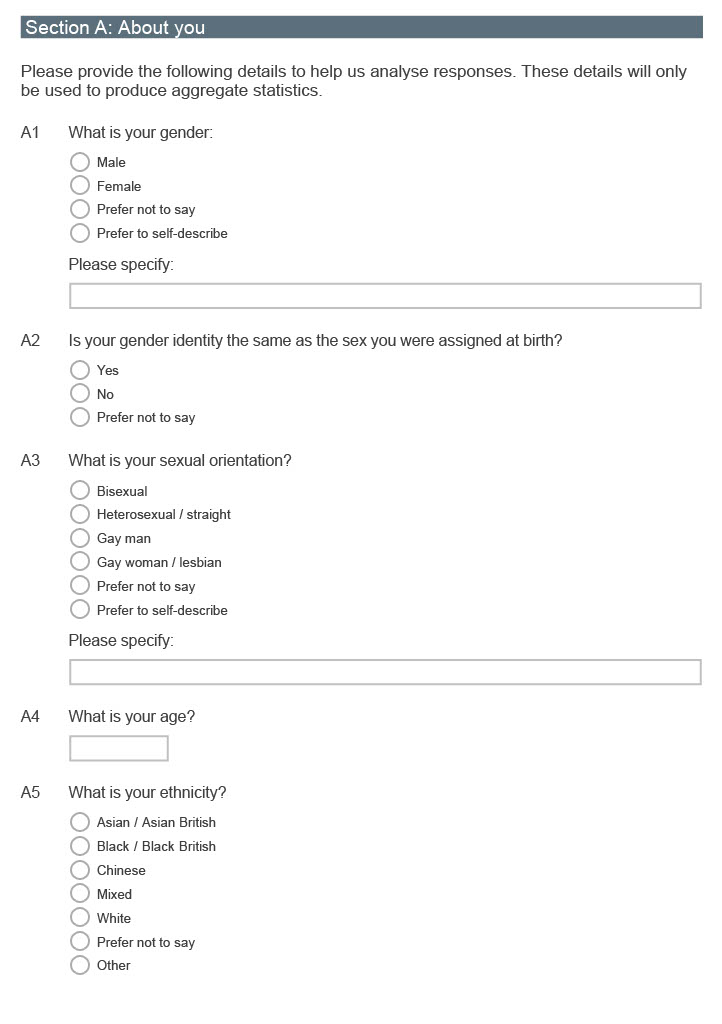
**

**
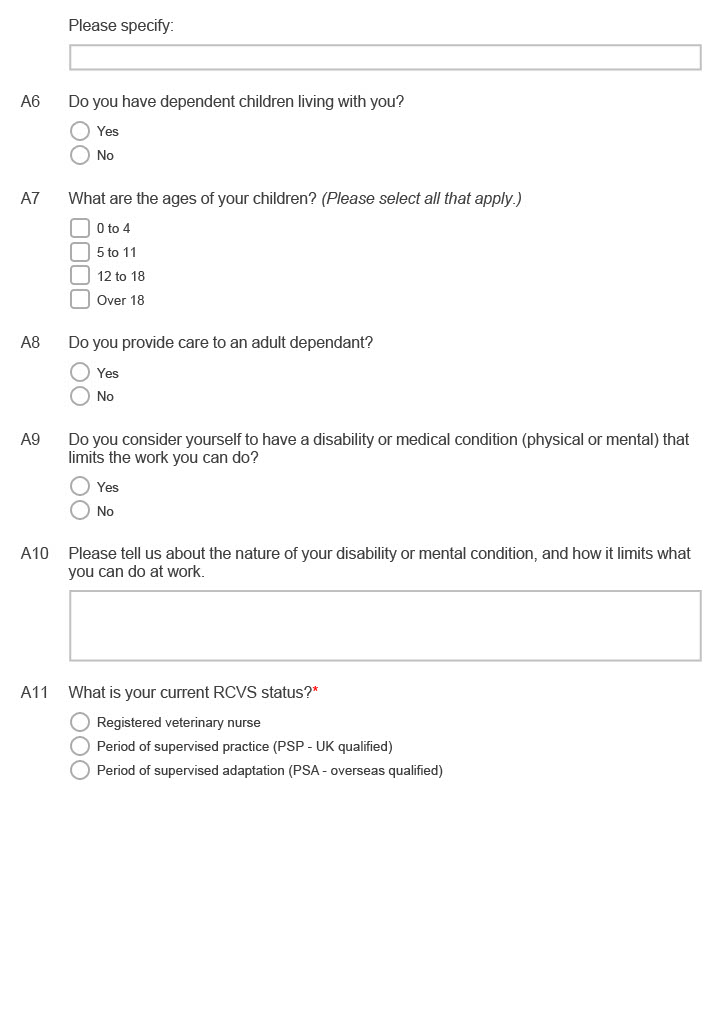
**

**
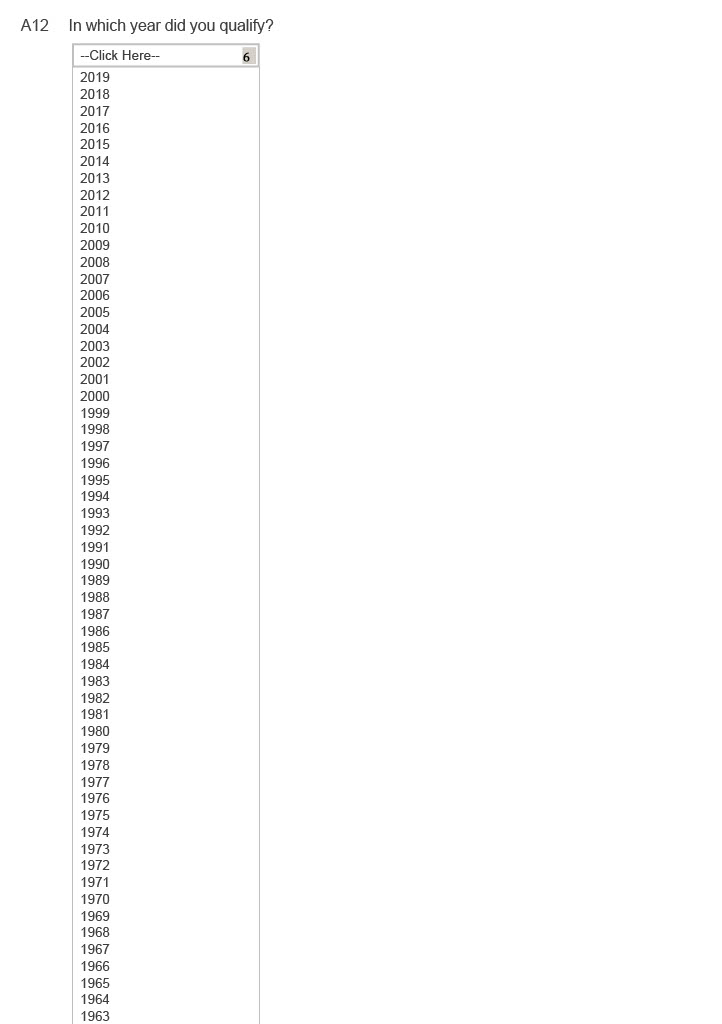
**

**
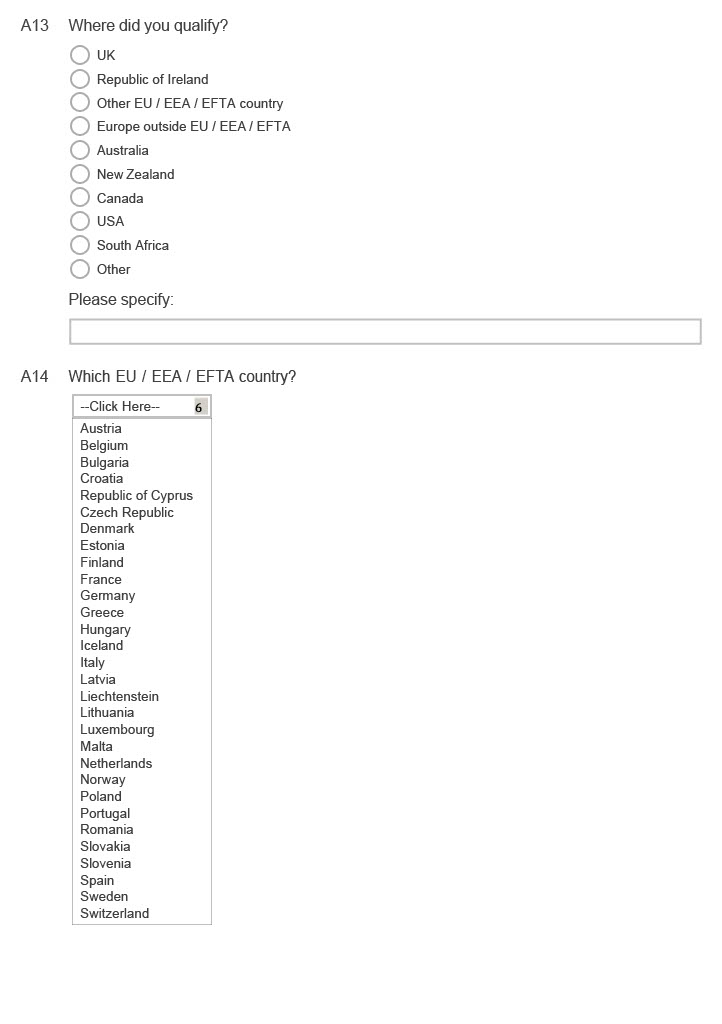
**

**
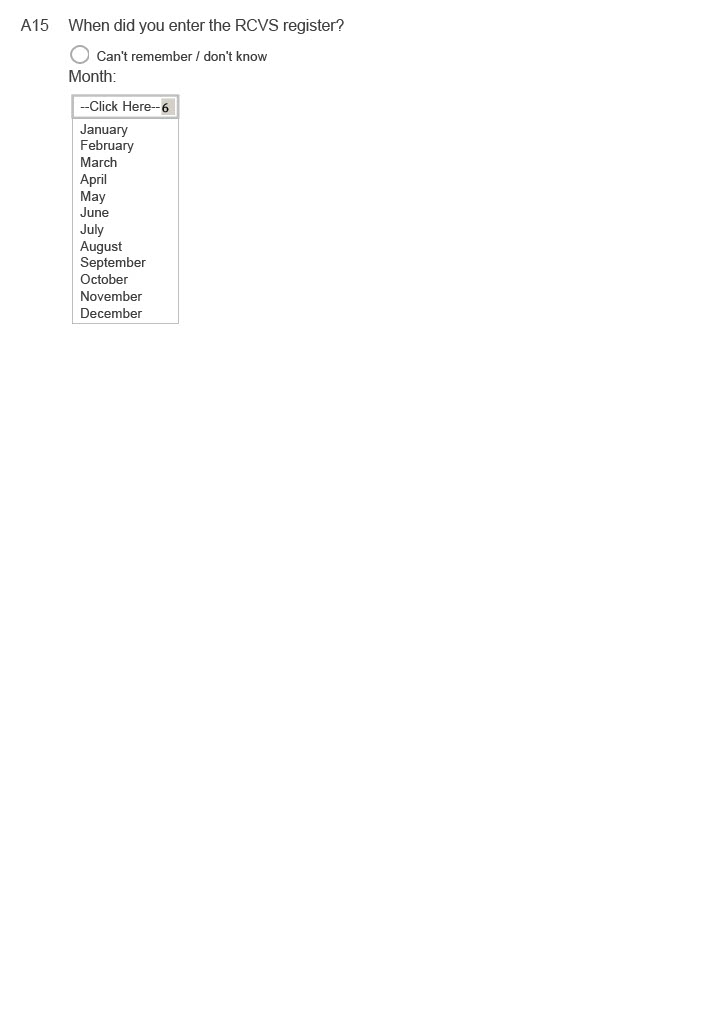
**

**
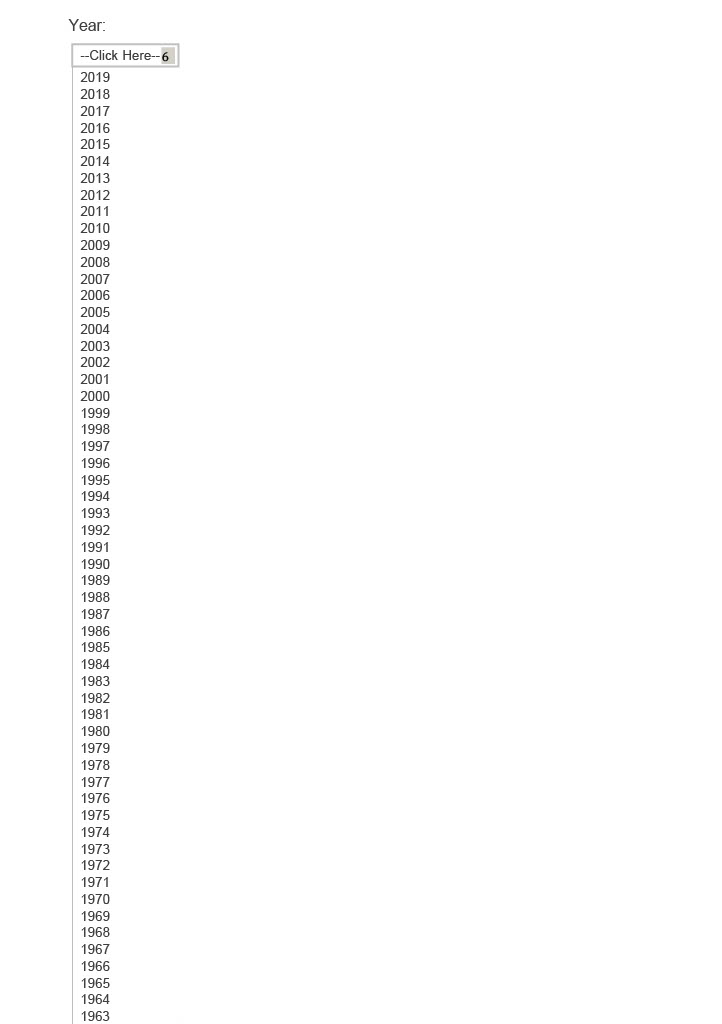
**

**
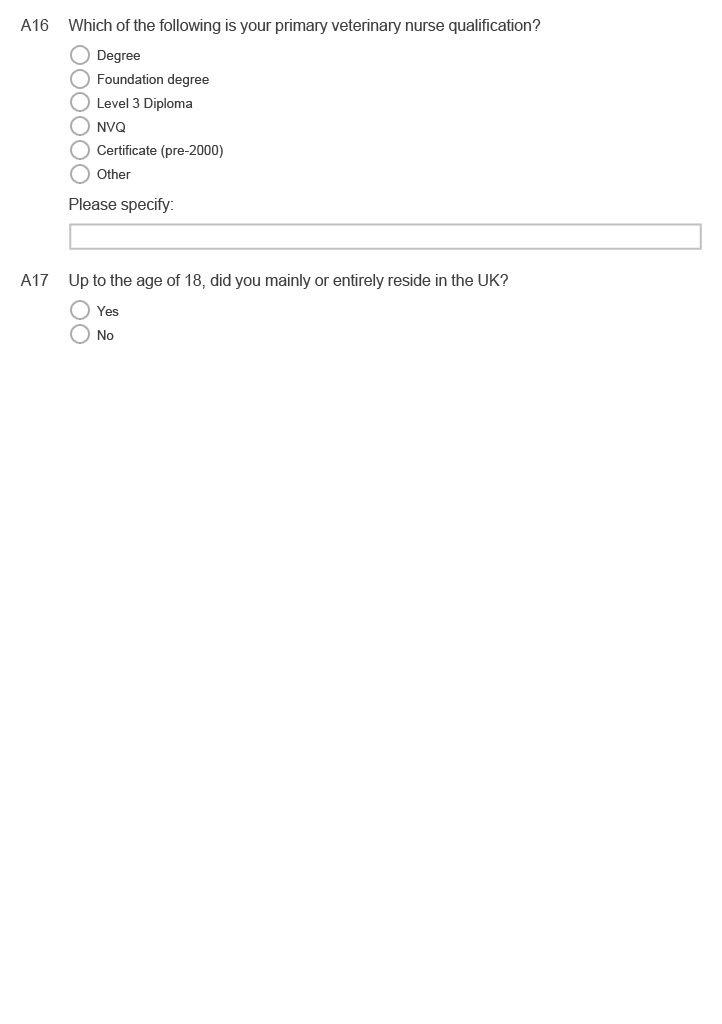
**

**
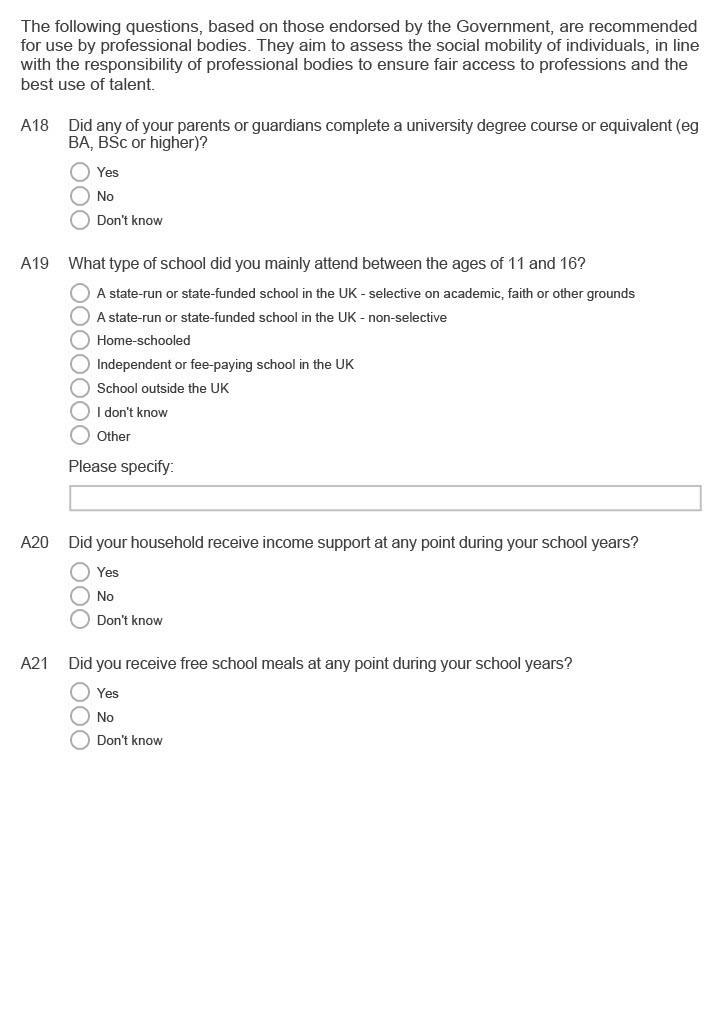
**

**
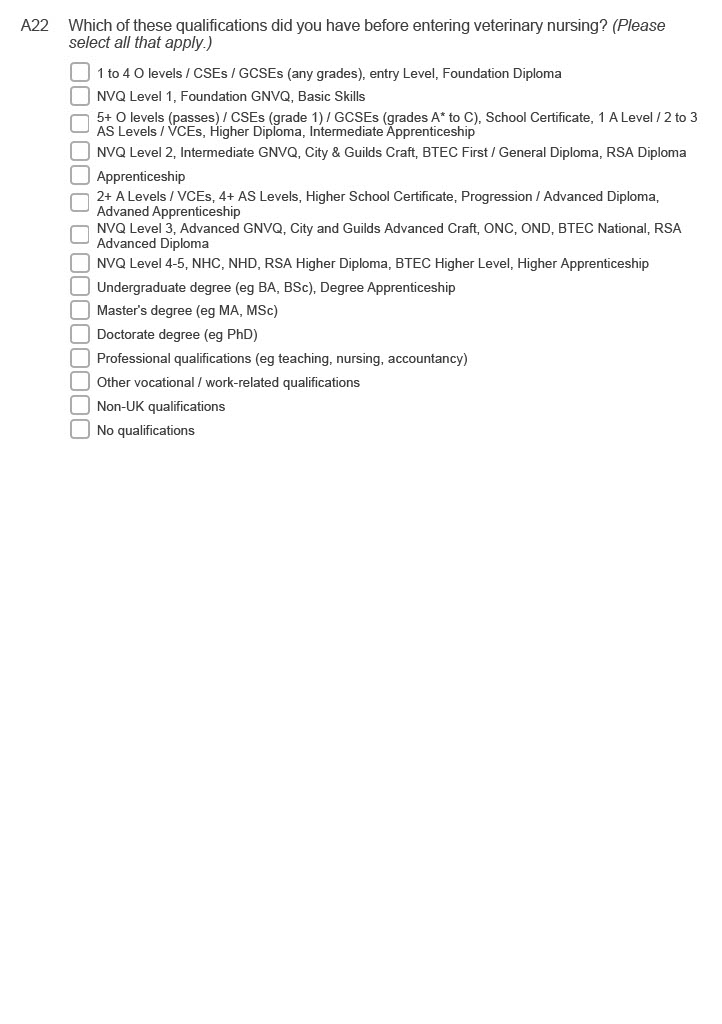
**

**
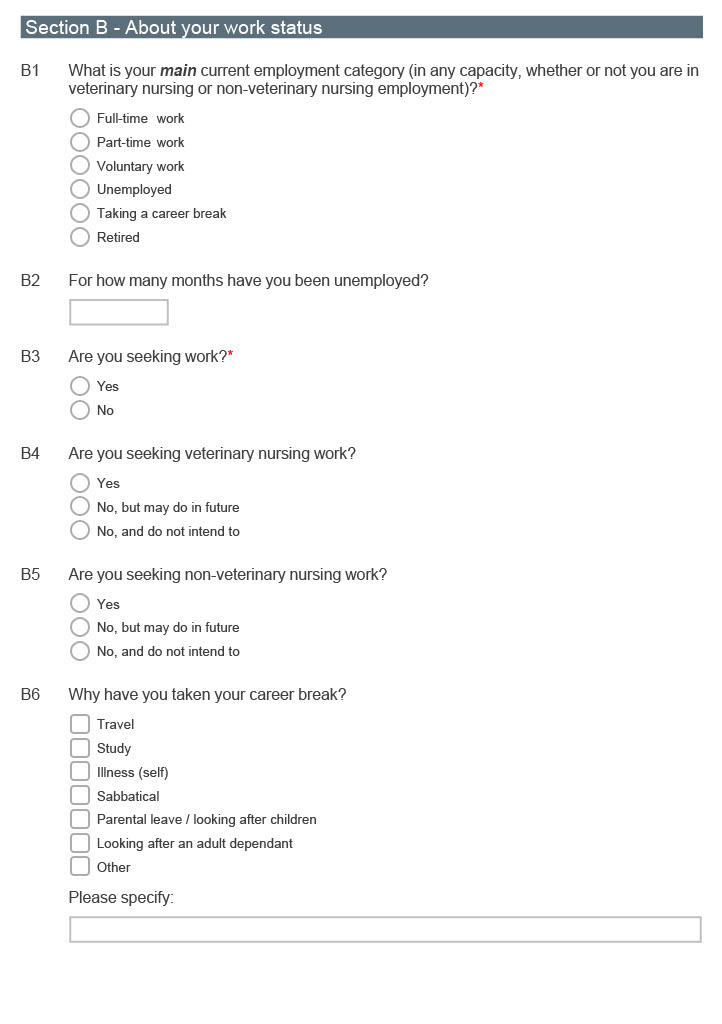
**

**
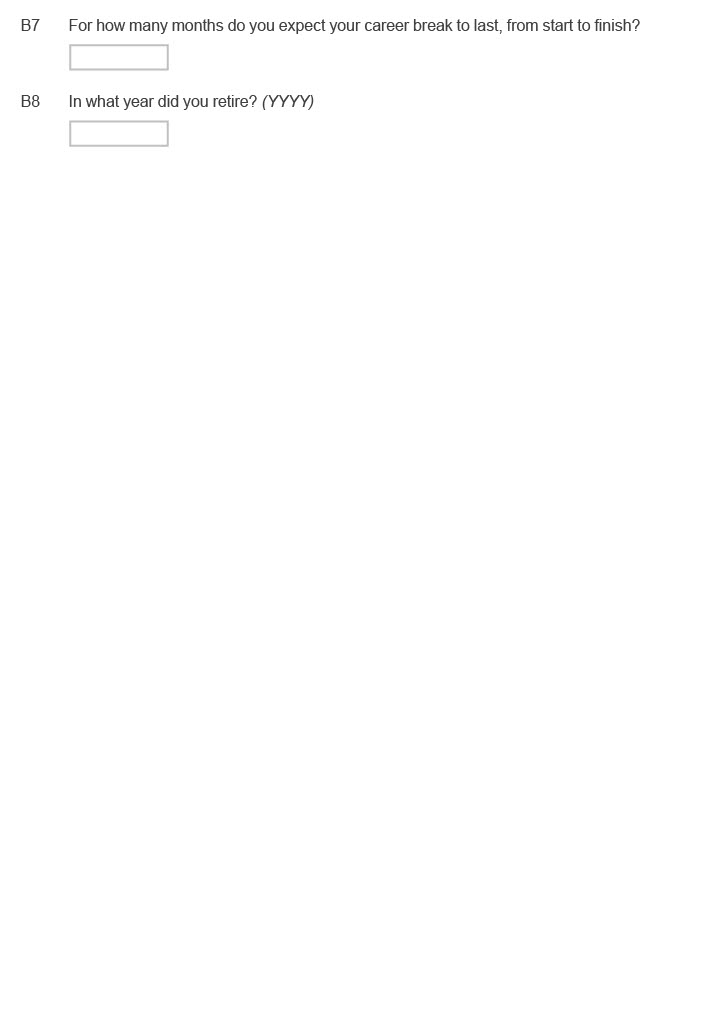
**

**
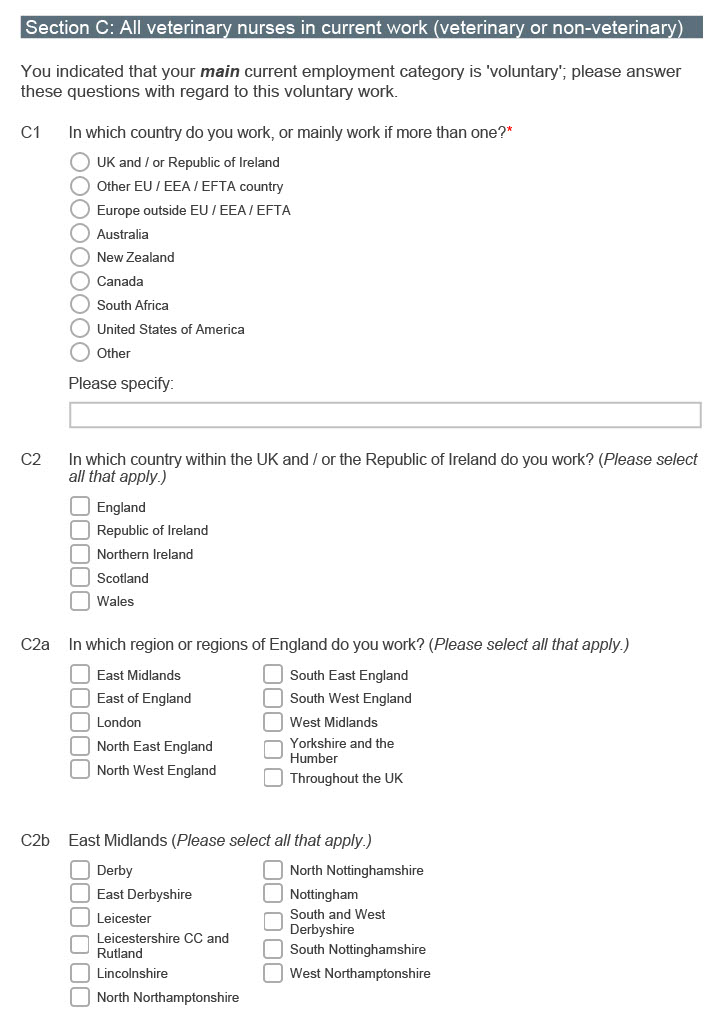
**

**
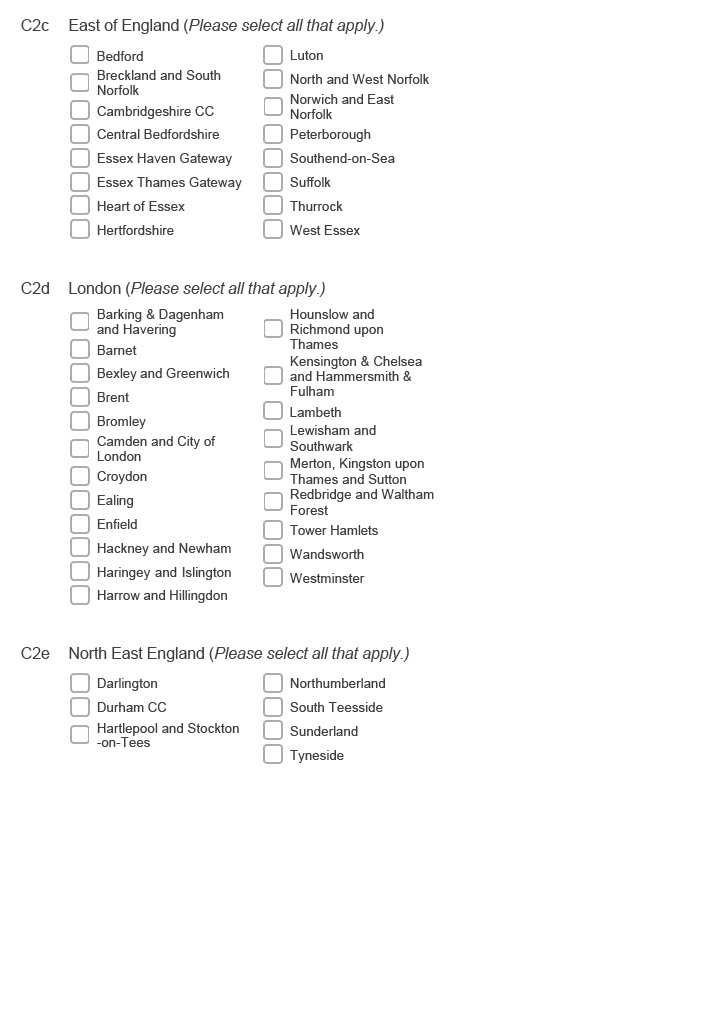
**

**
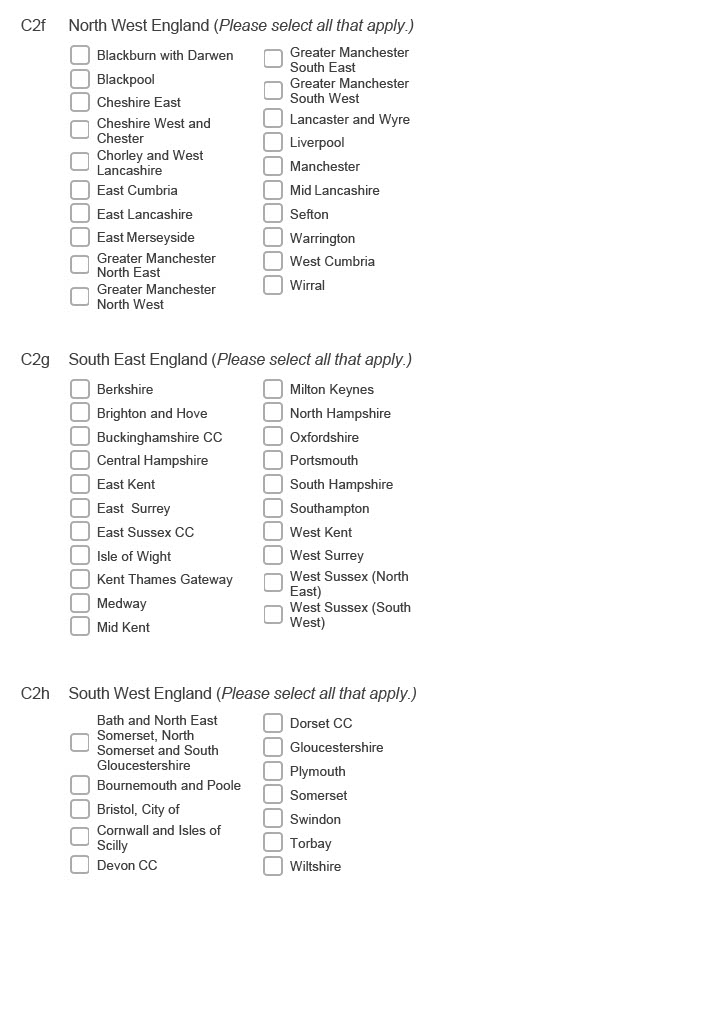
**

**
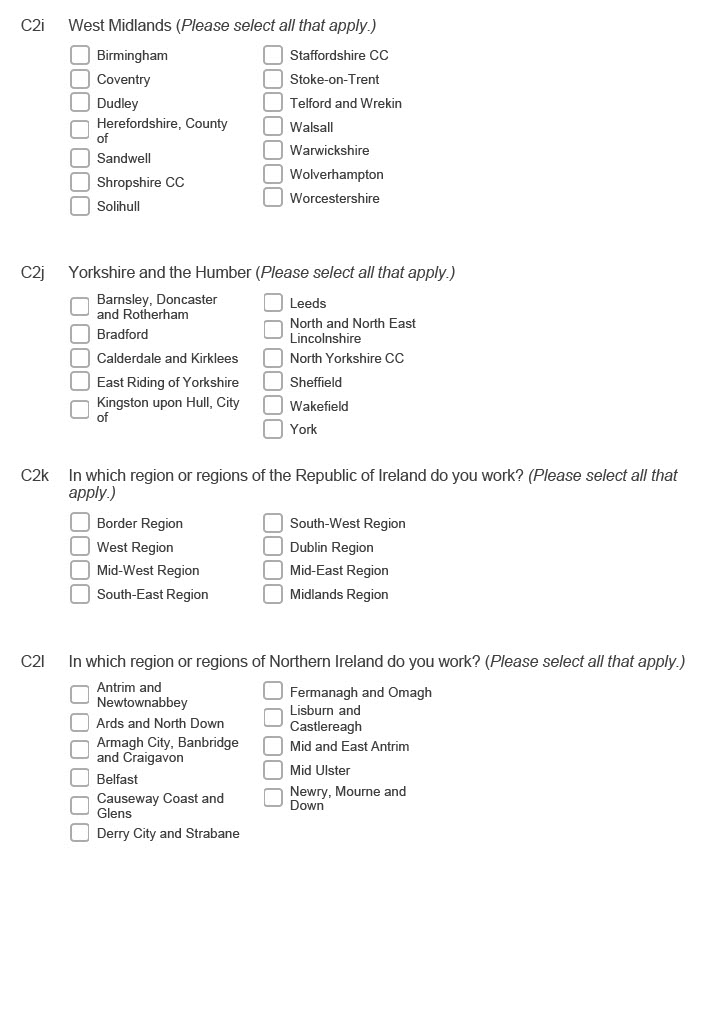
**

**
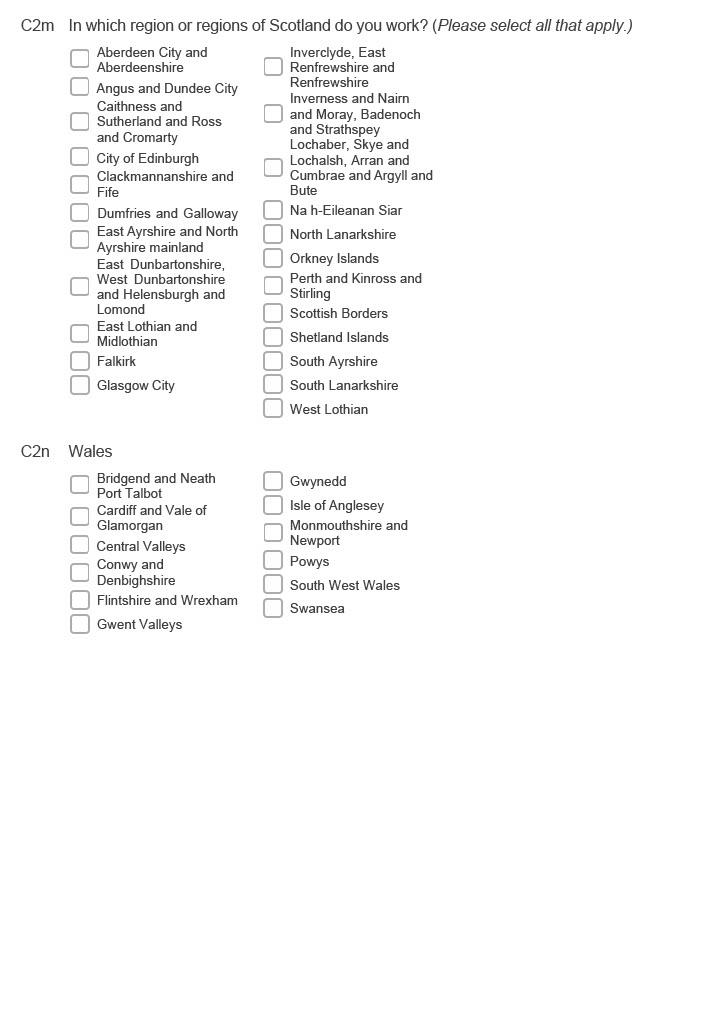
**

**
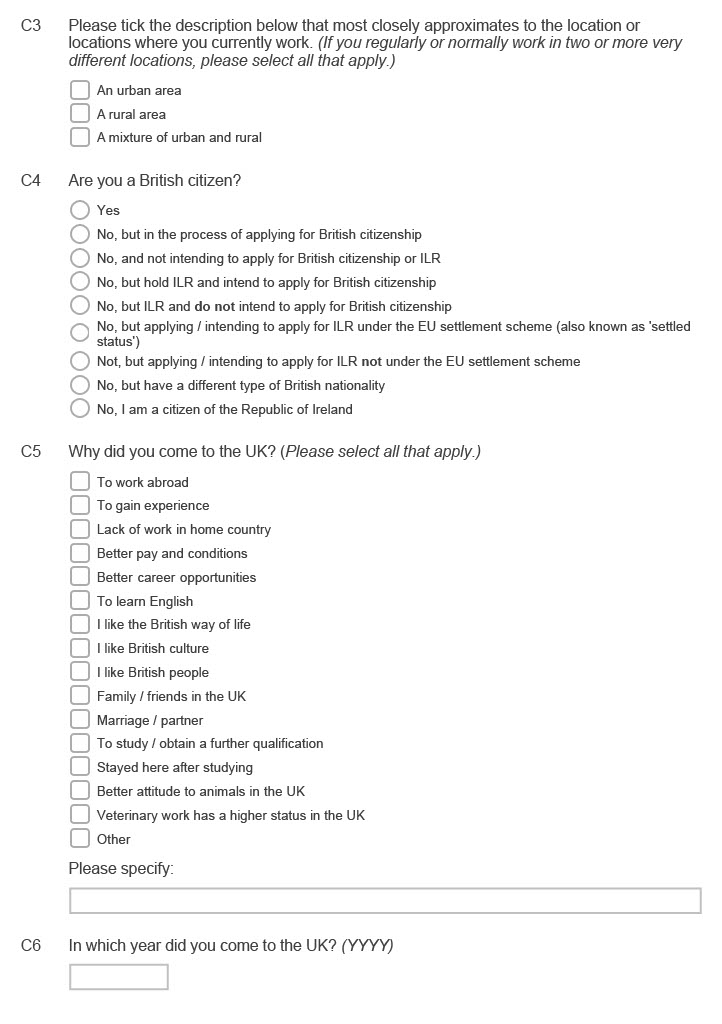
**

**
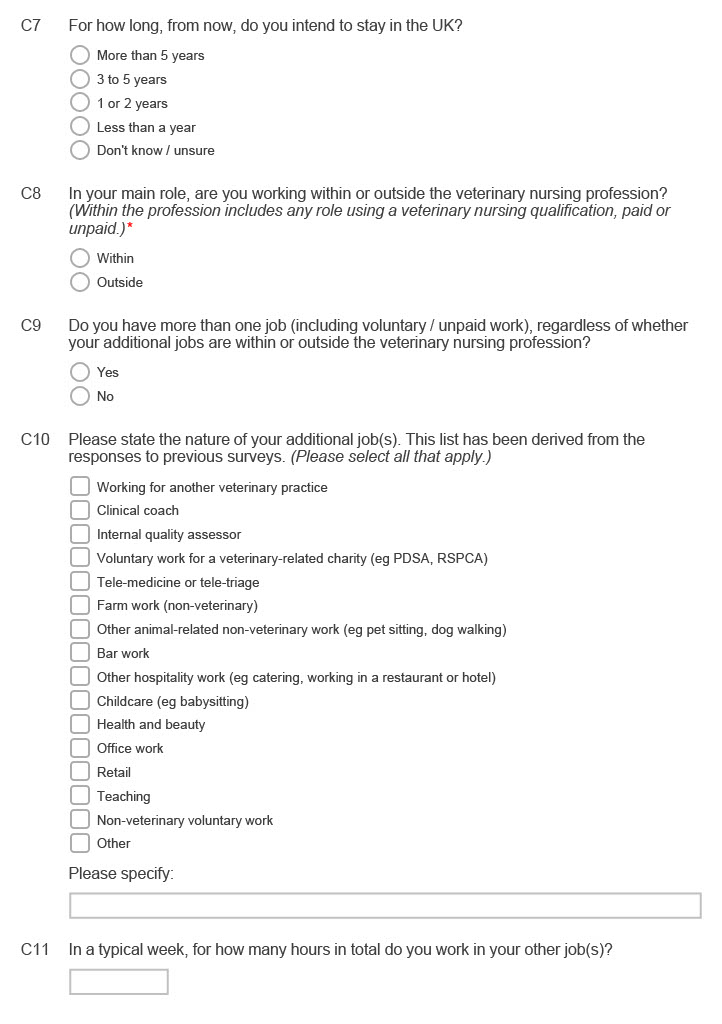
**

**
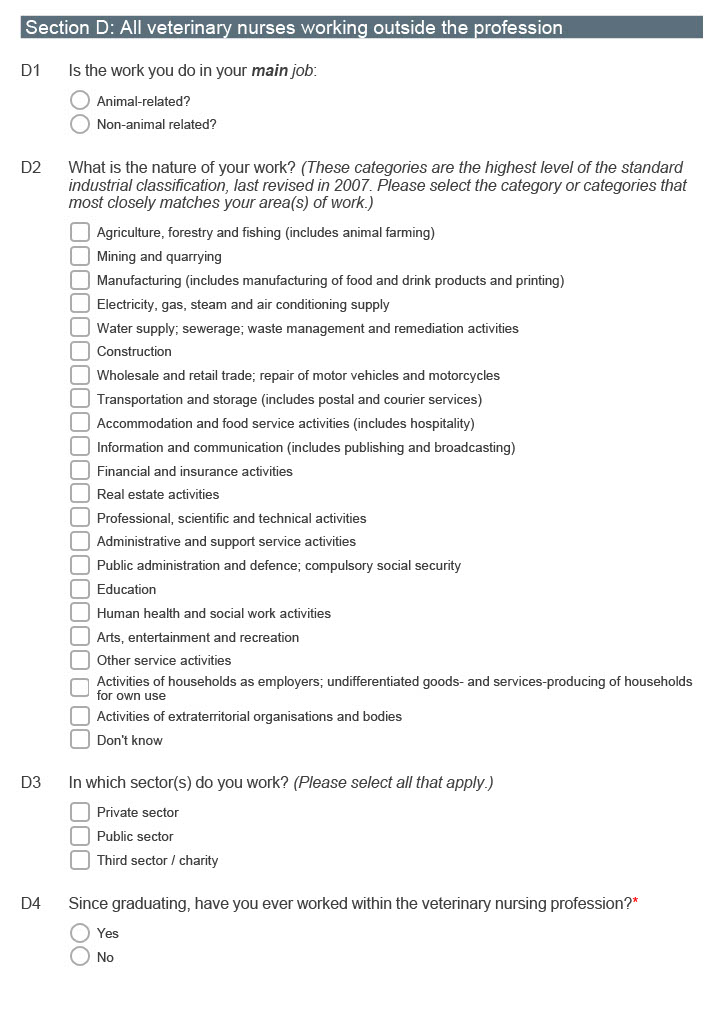
**

**
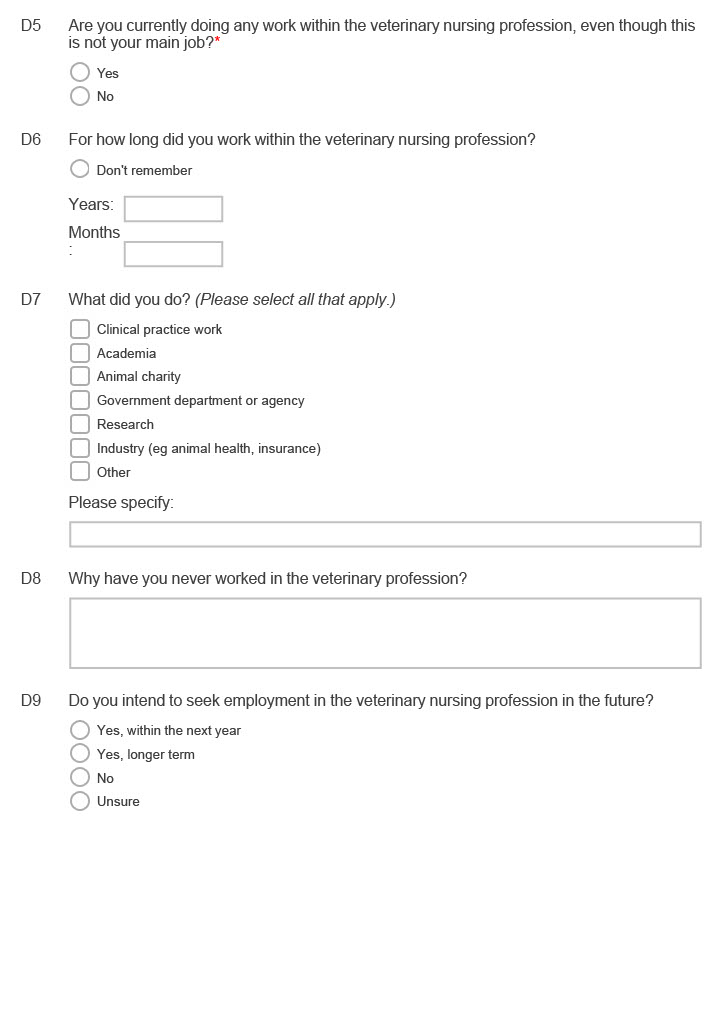
**

**
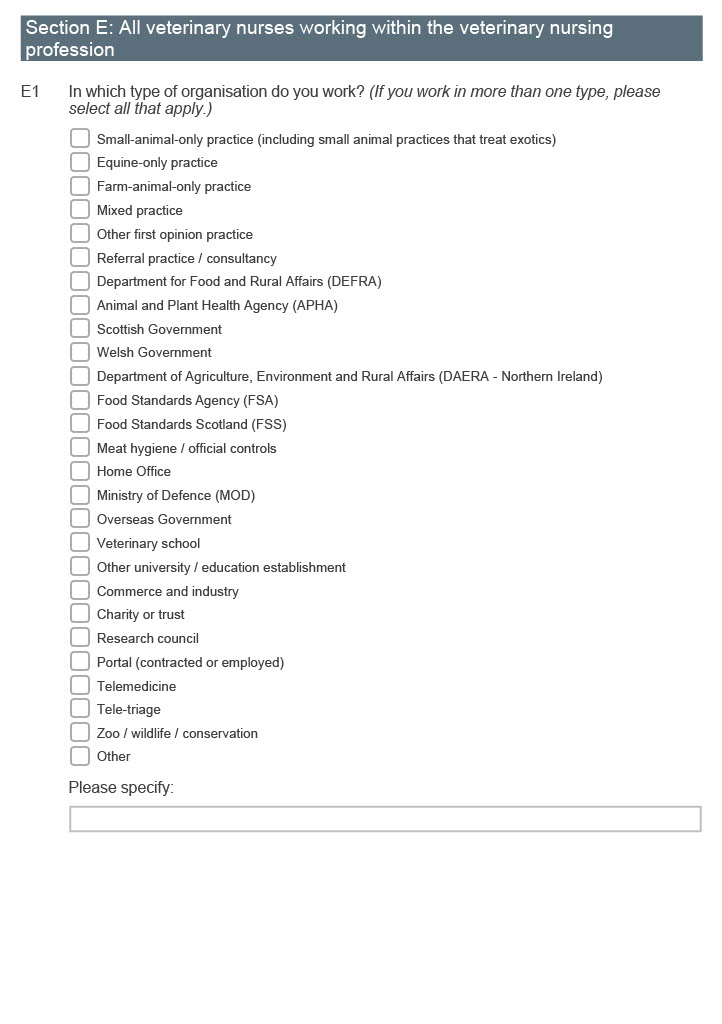
**

**
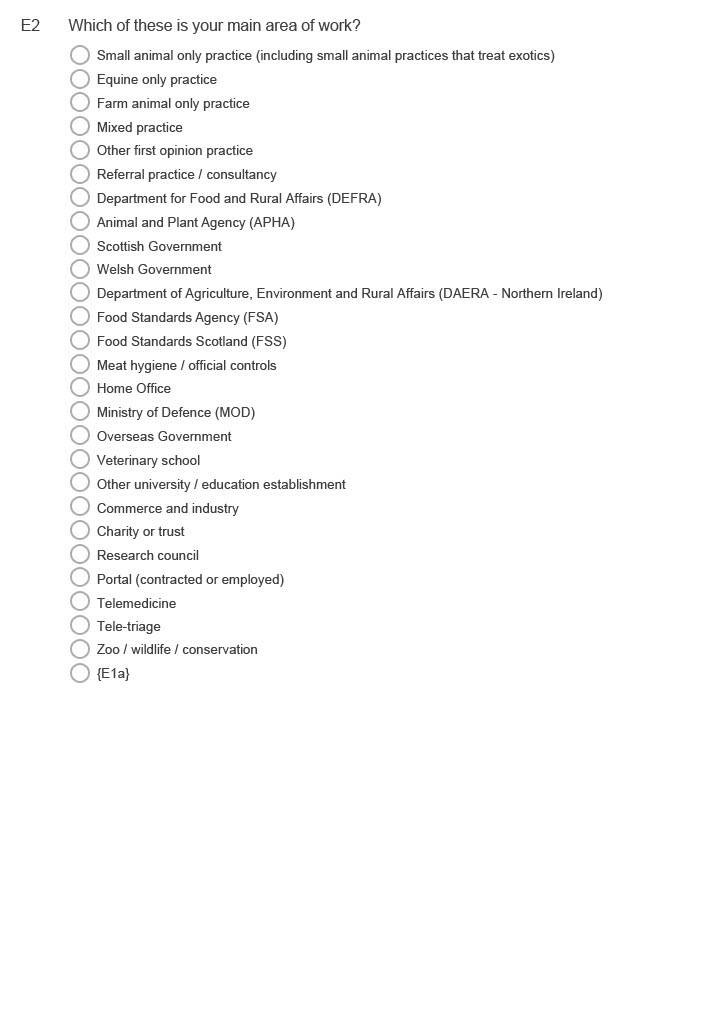
**

**
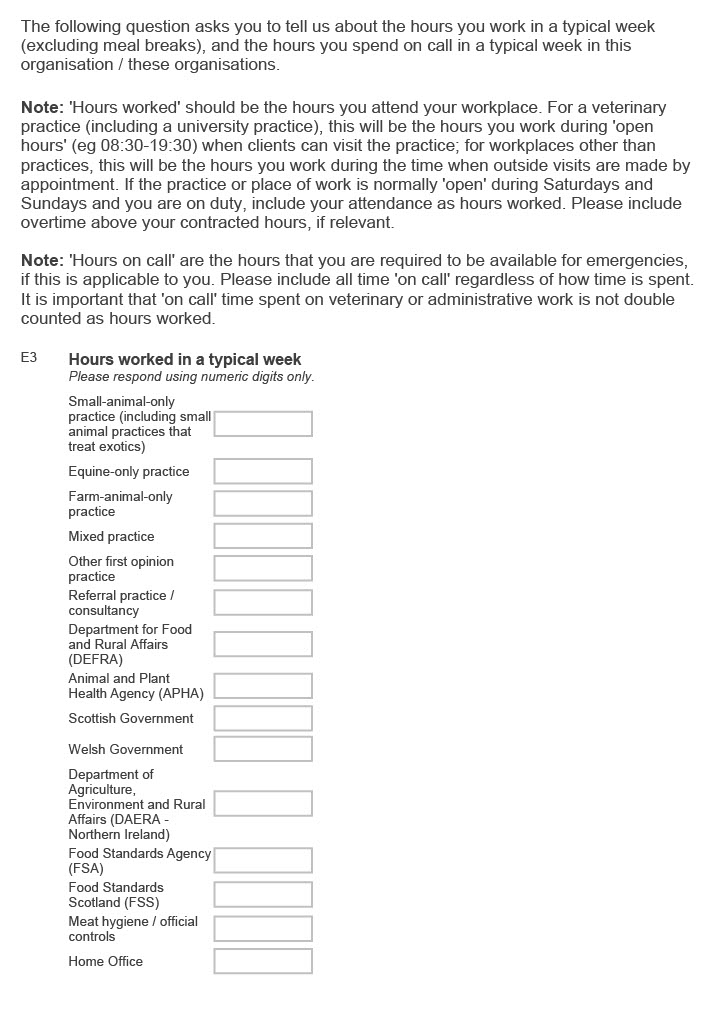
**

**
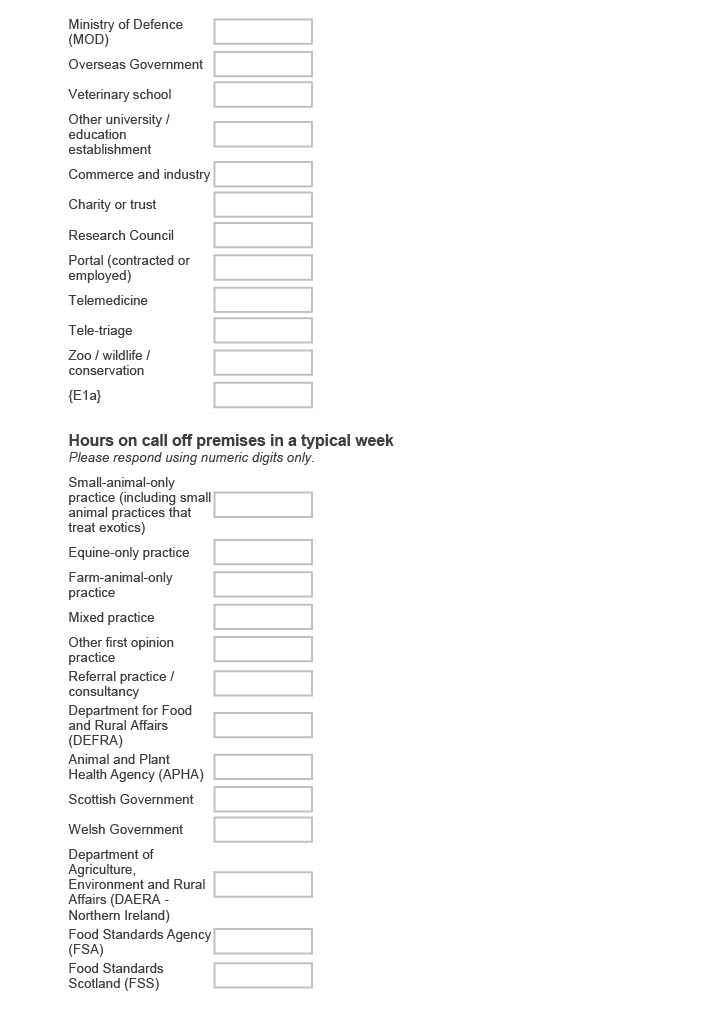
**

**
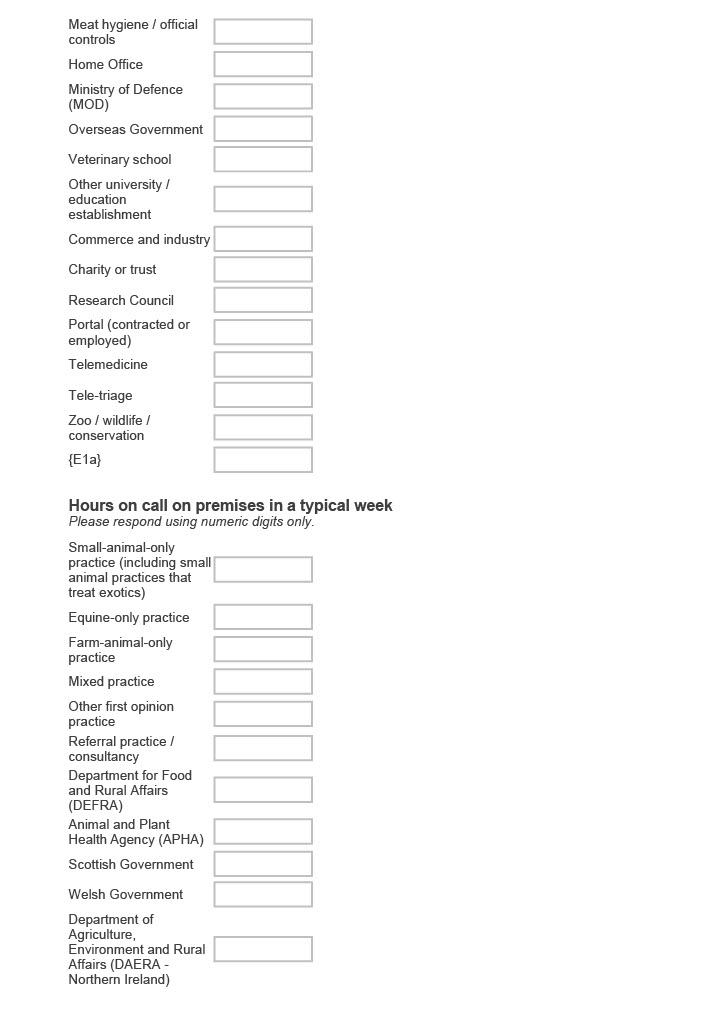
**

**
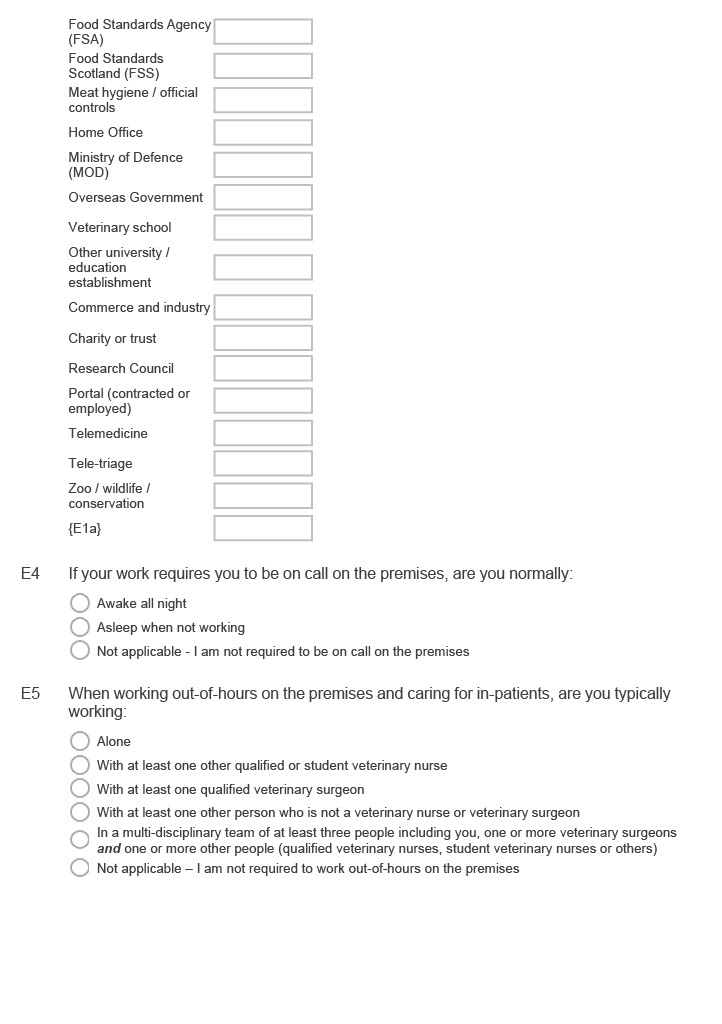
**

**
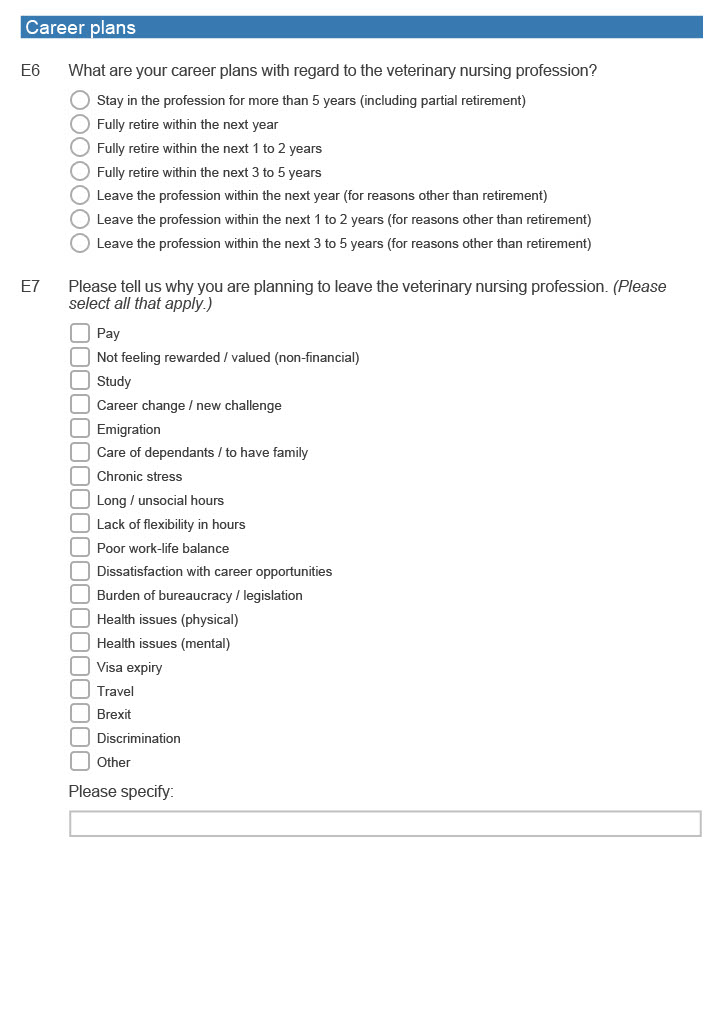
**

**
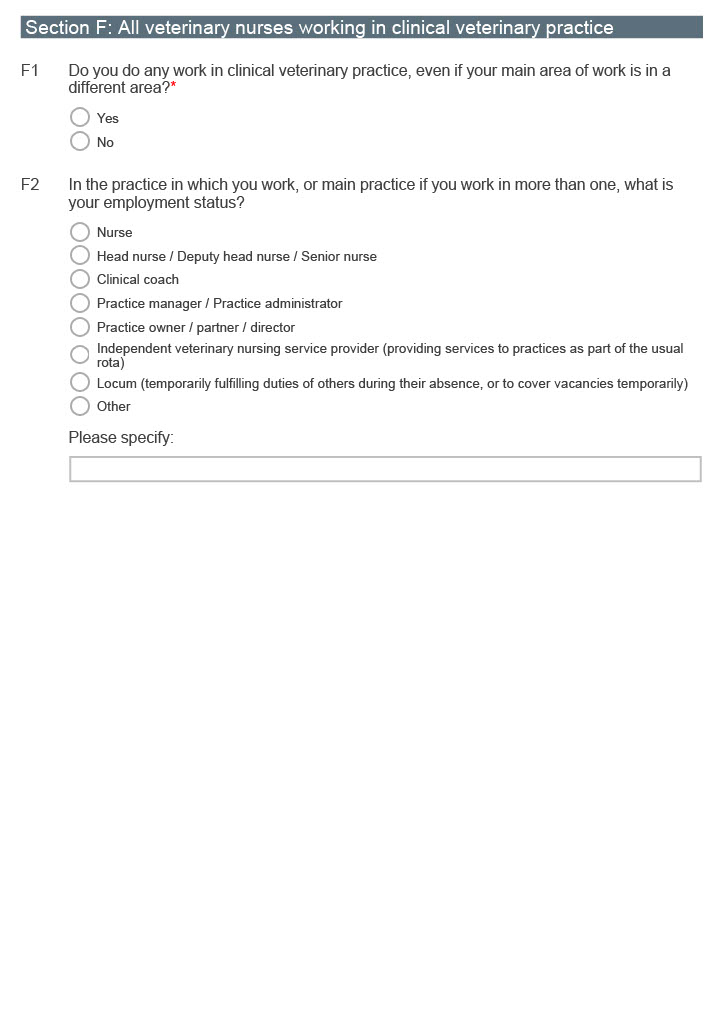
**

**
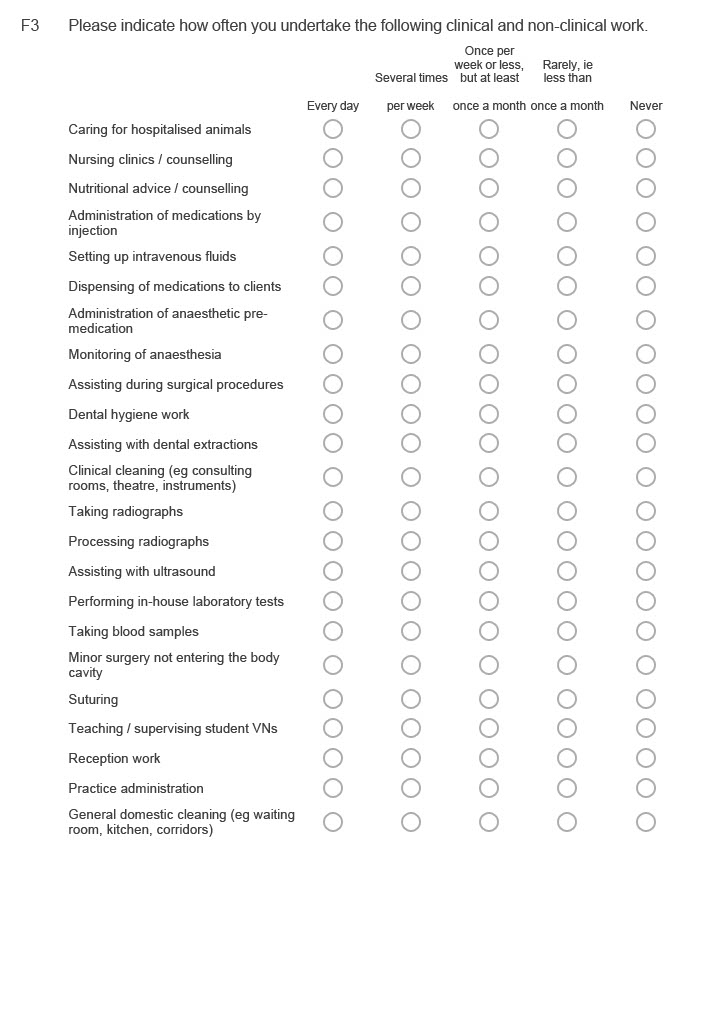
**

**
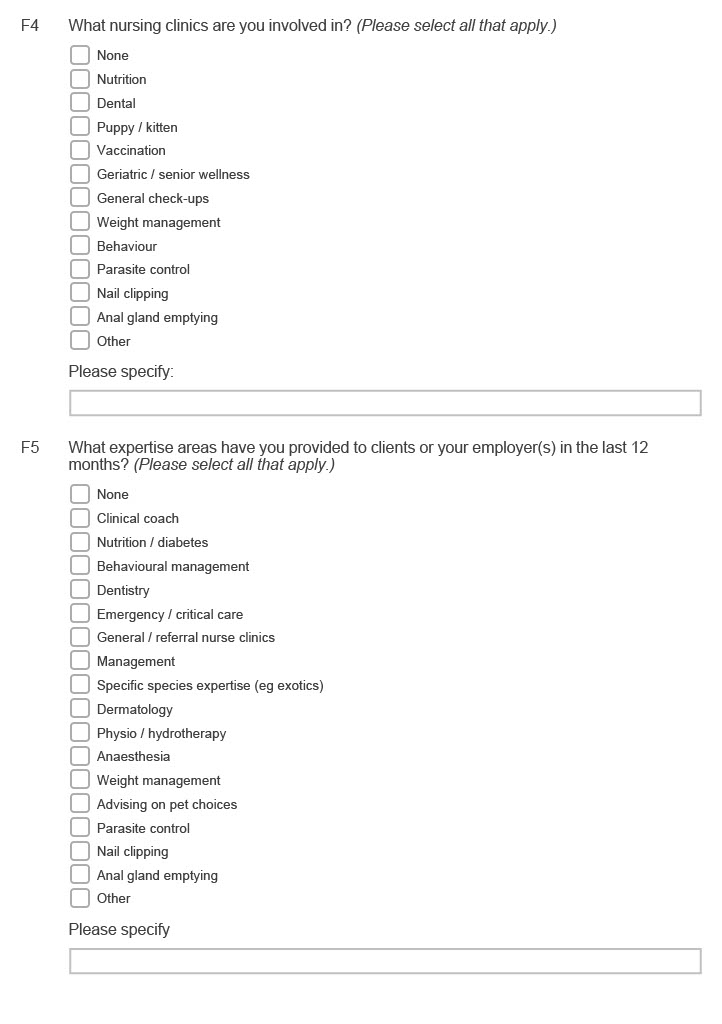
**

**
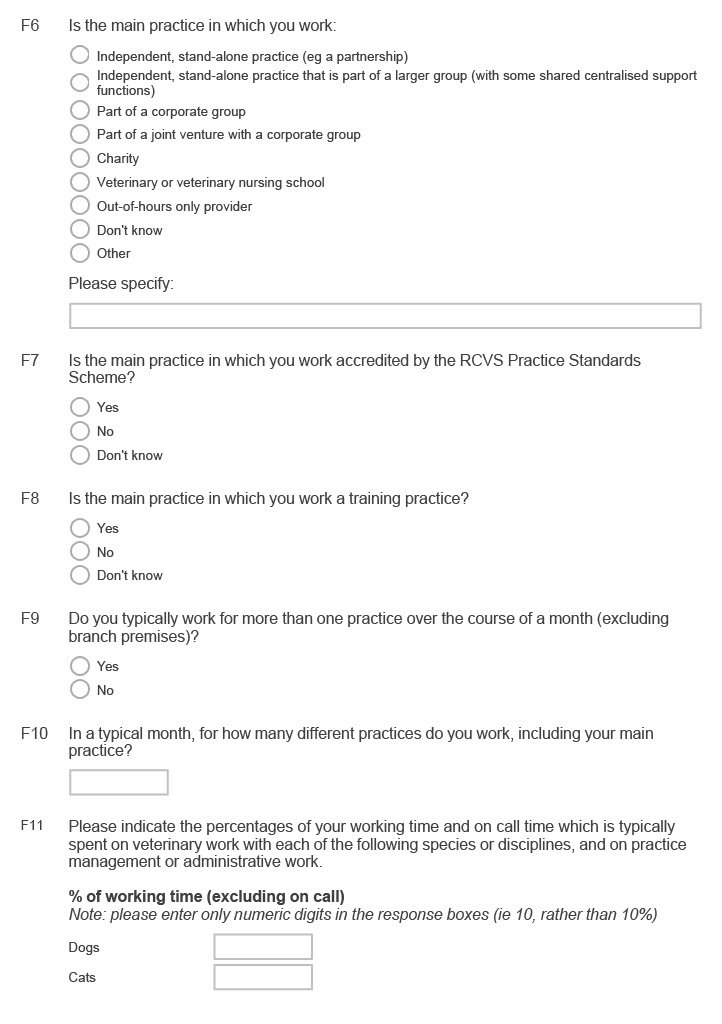
**

**
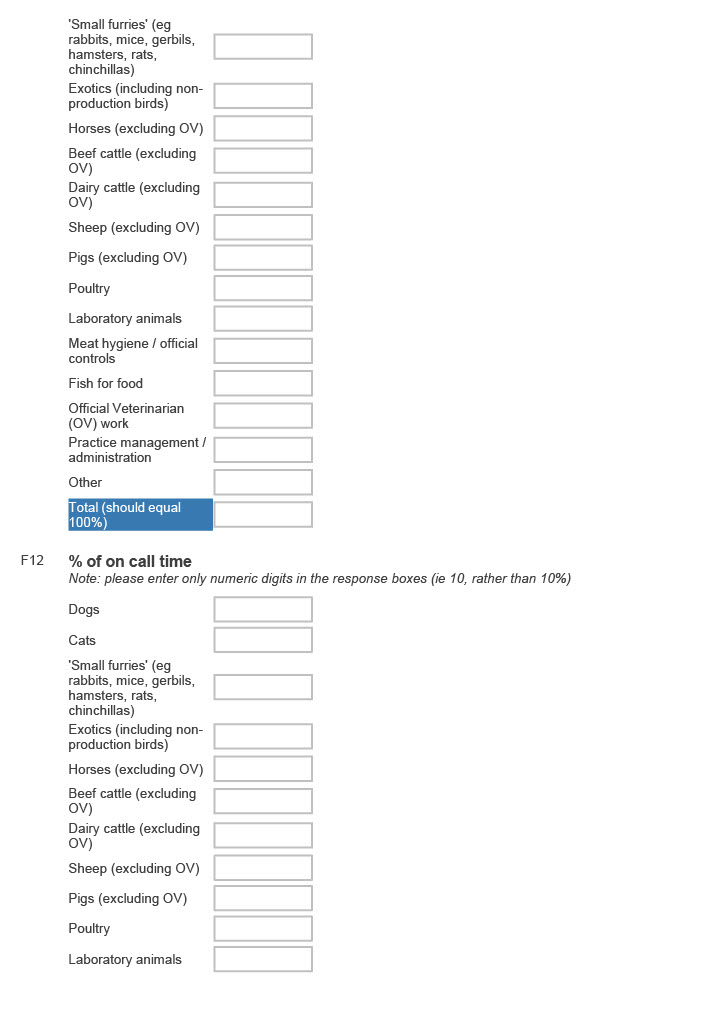
**

**
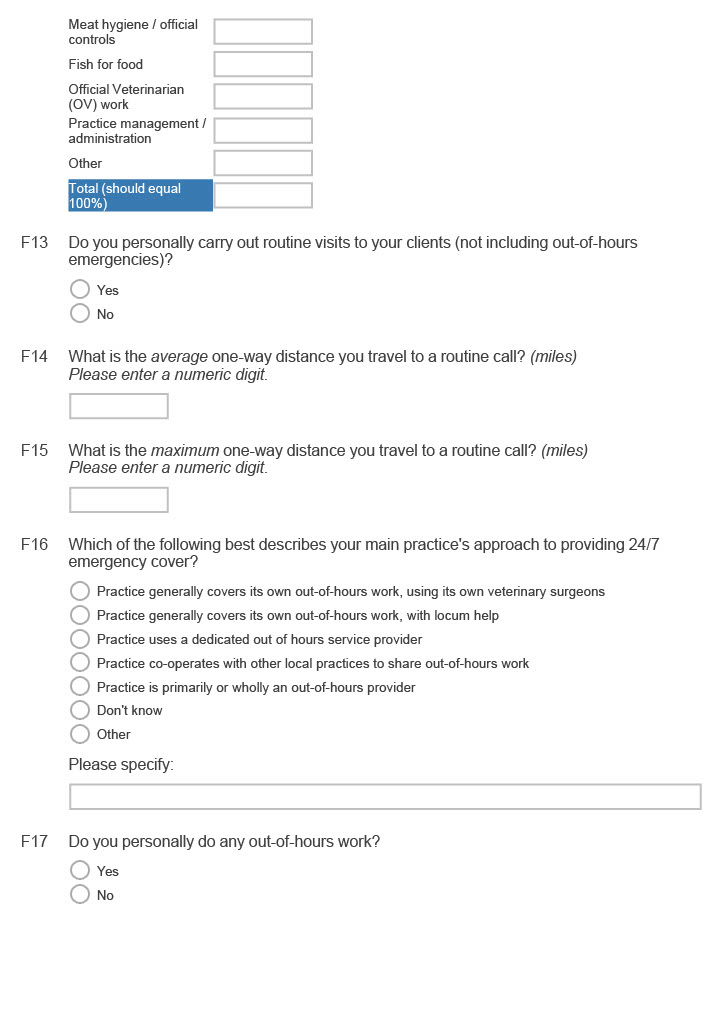
**

**
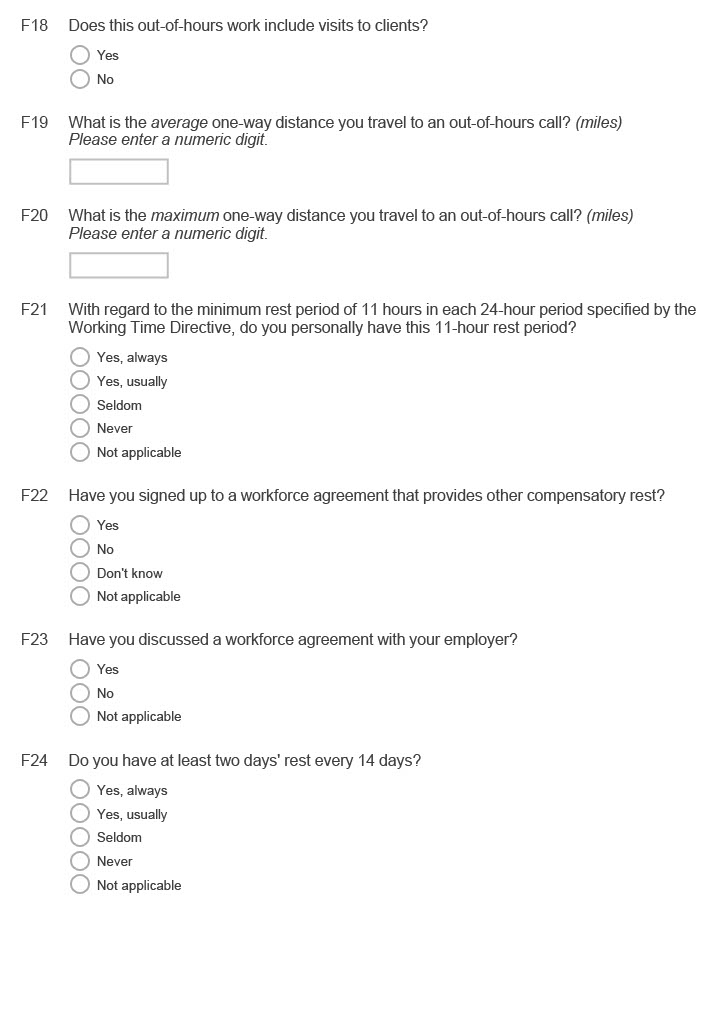
**

**
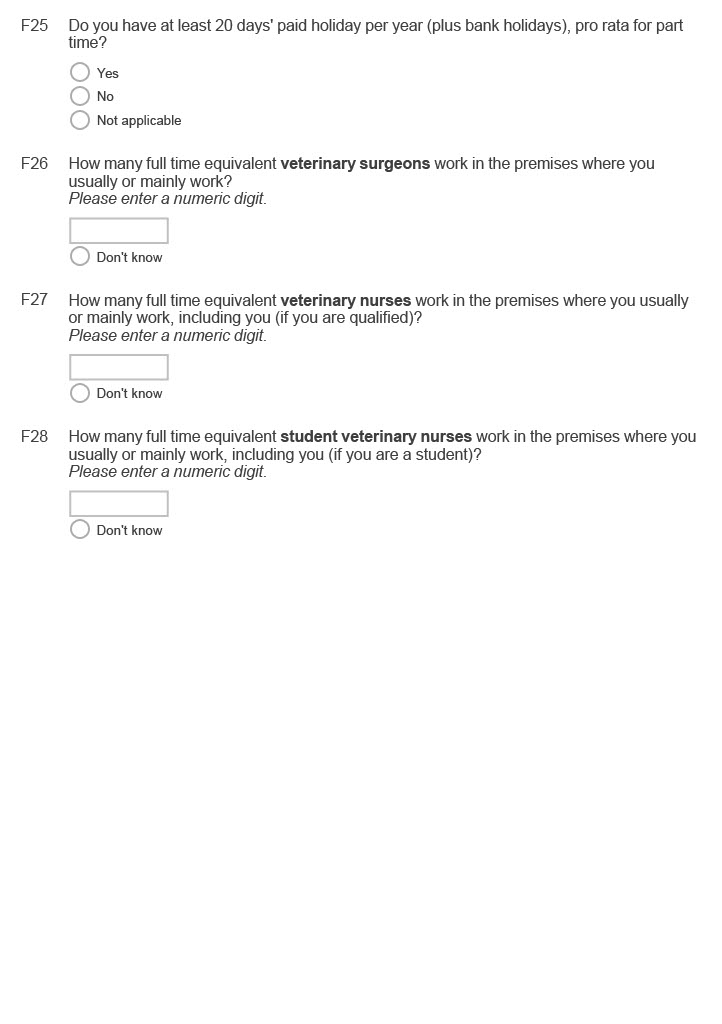
**

**
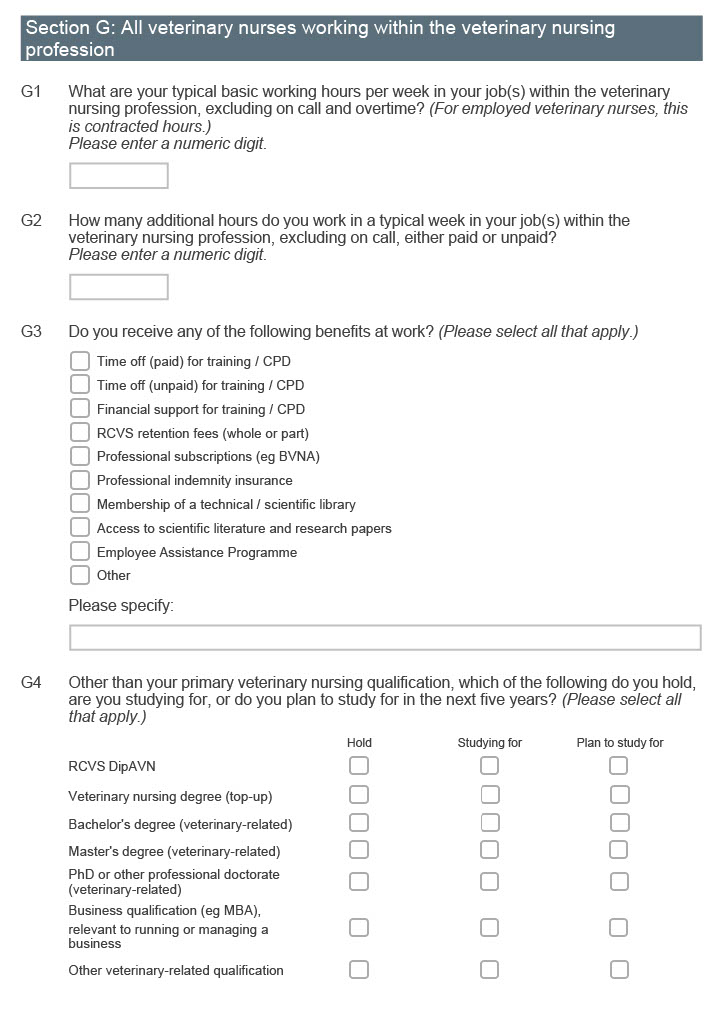
**

**
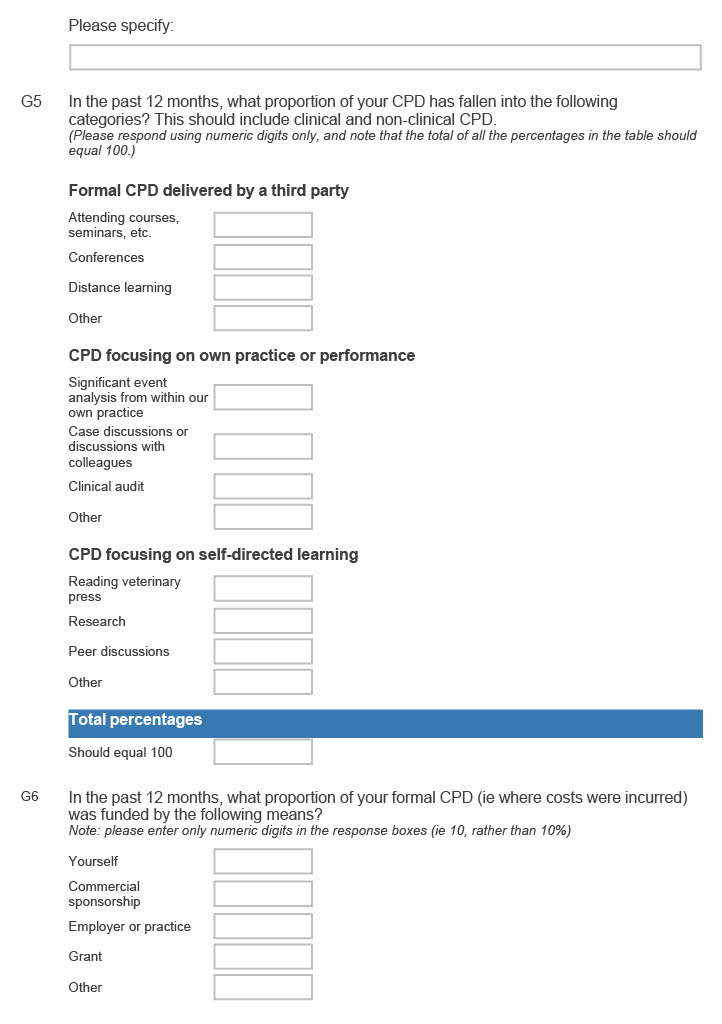
**

**
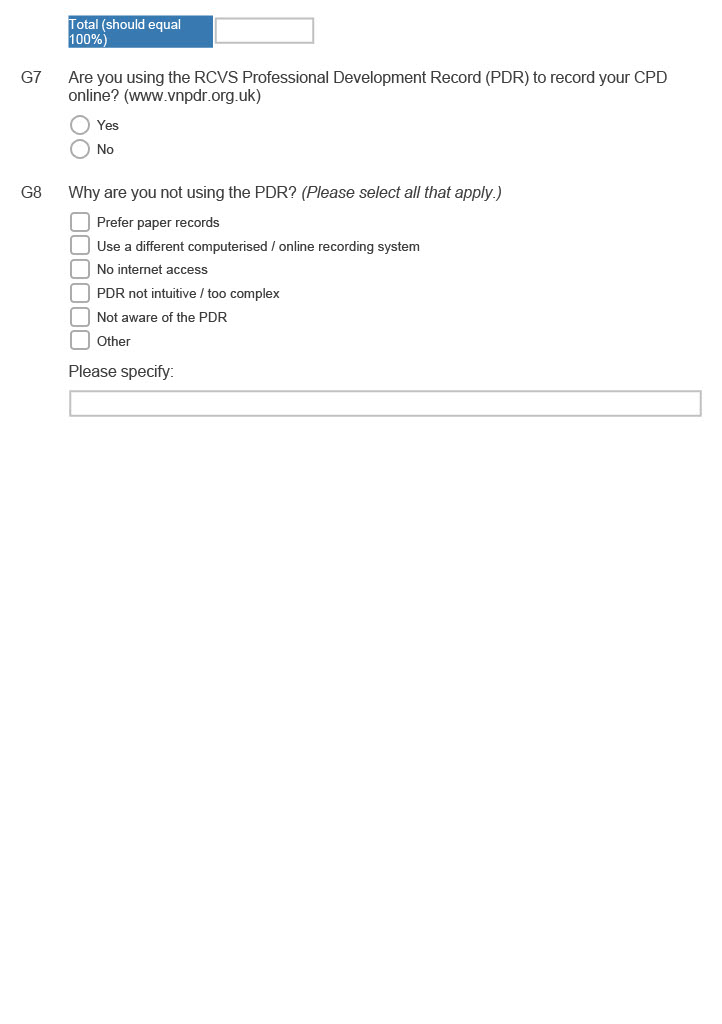

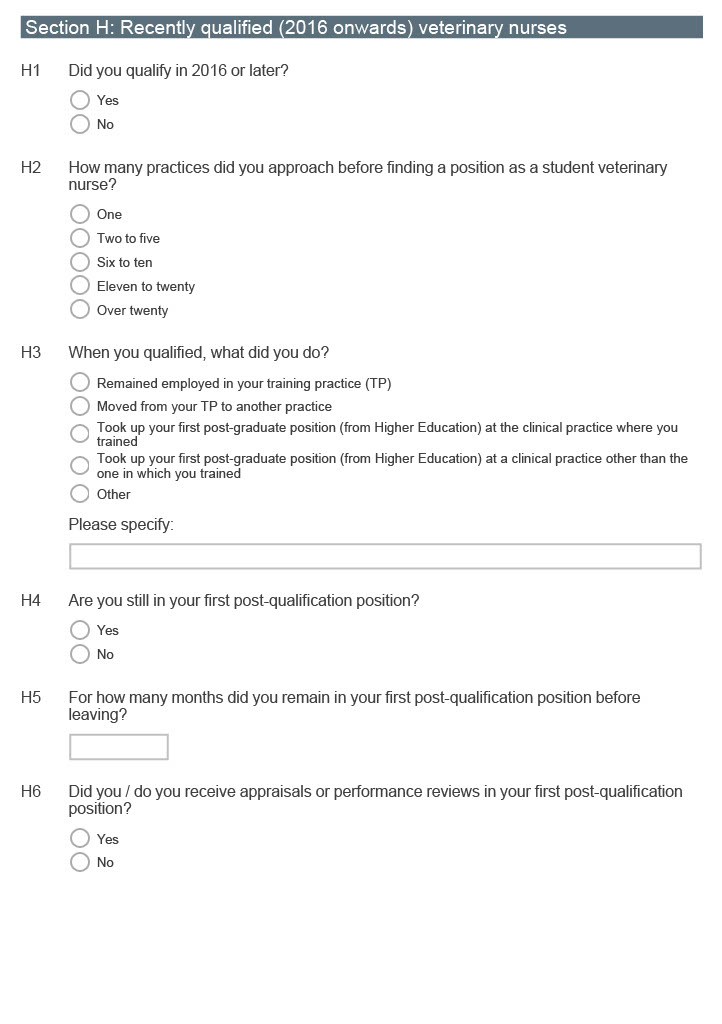
**

**
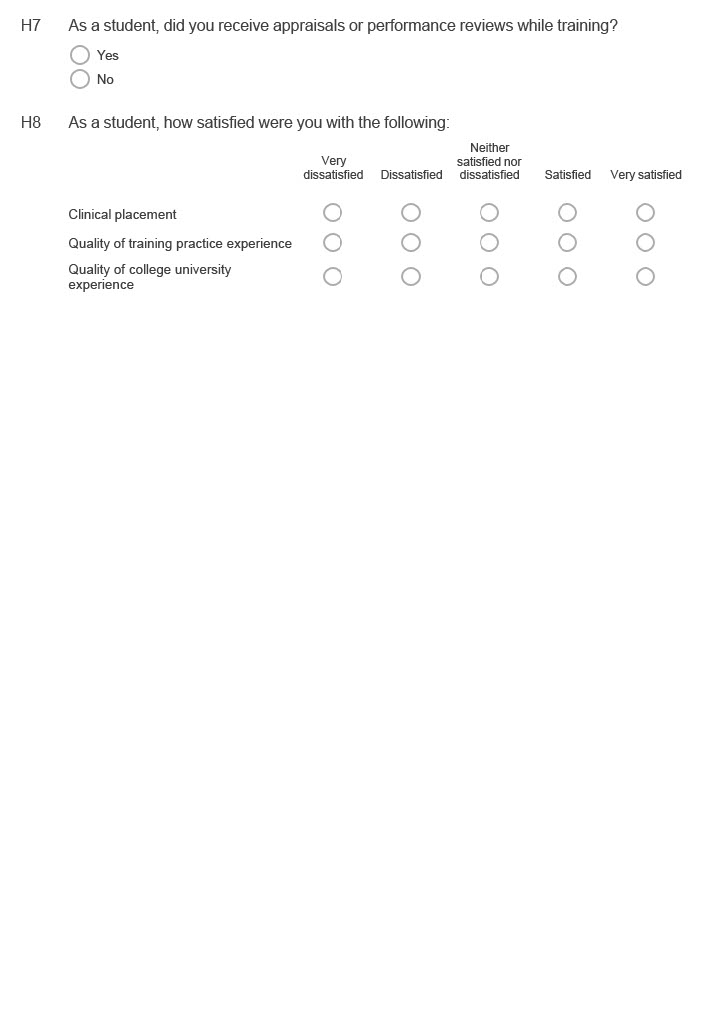
**

**
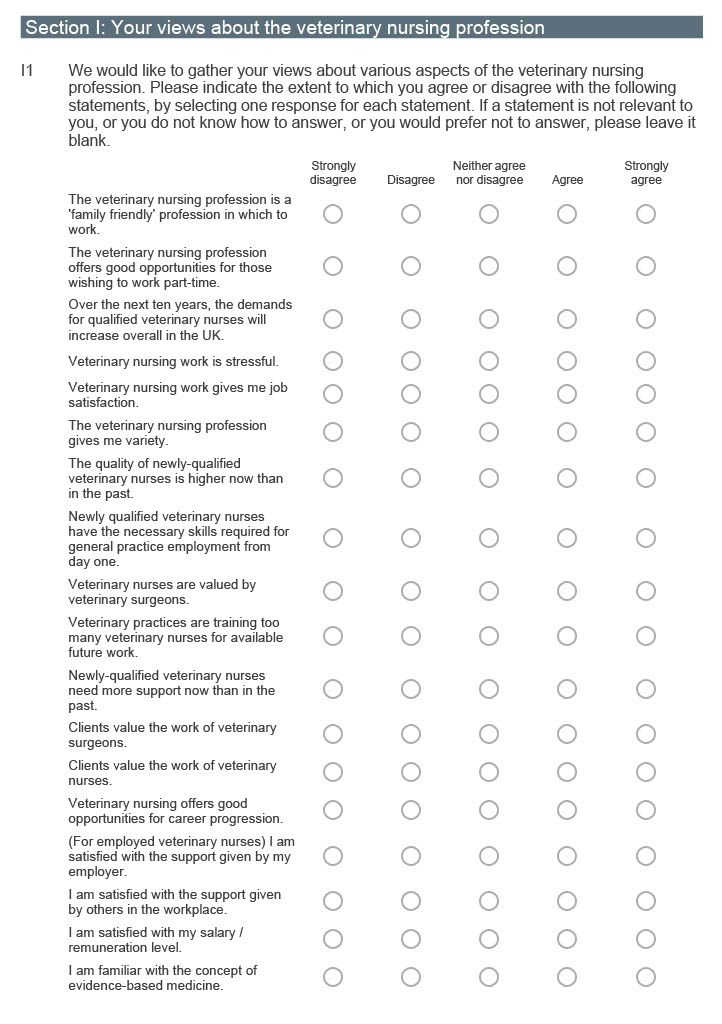
**

**
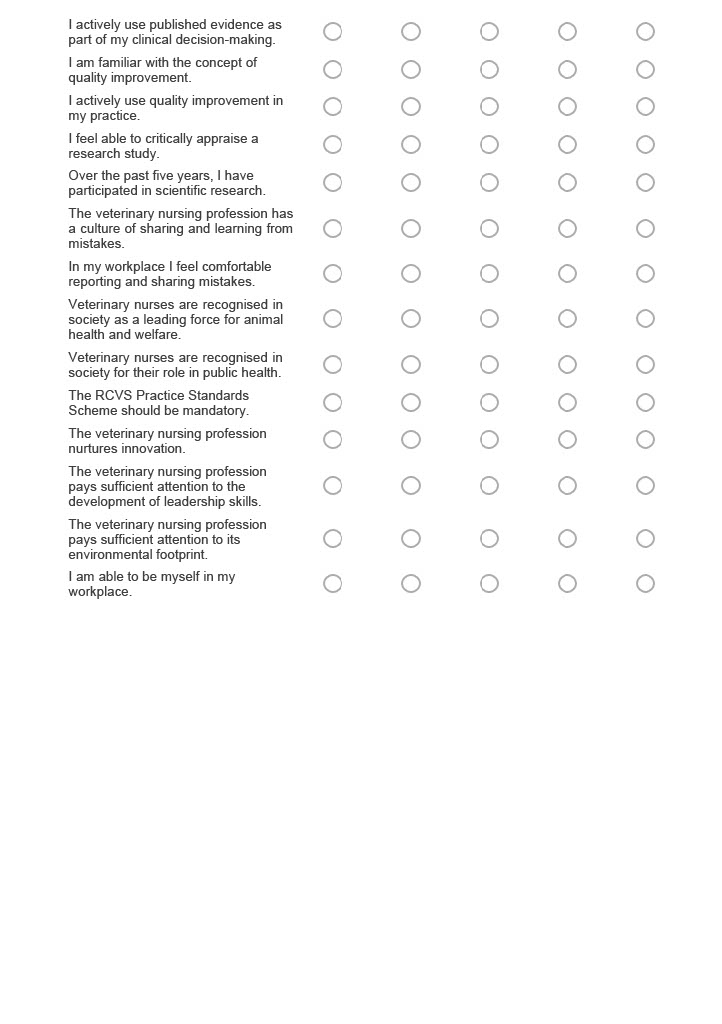
**

**
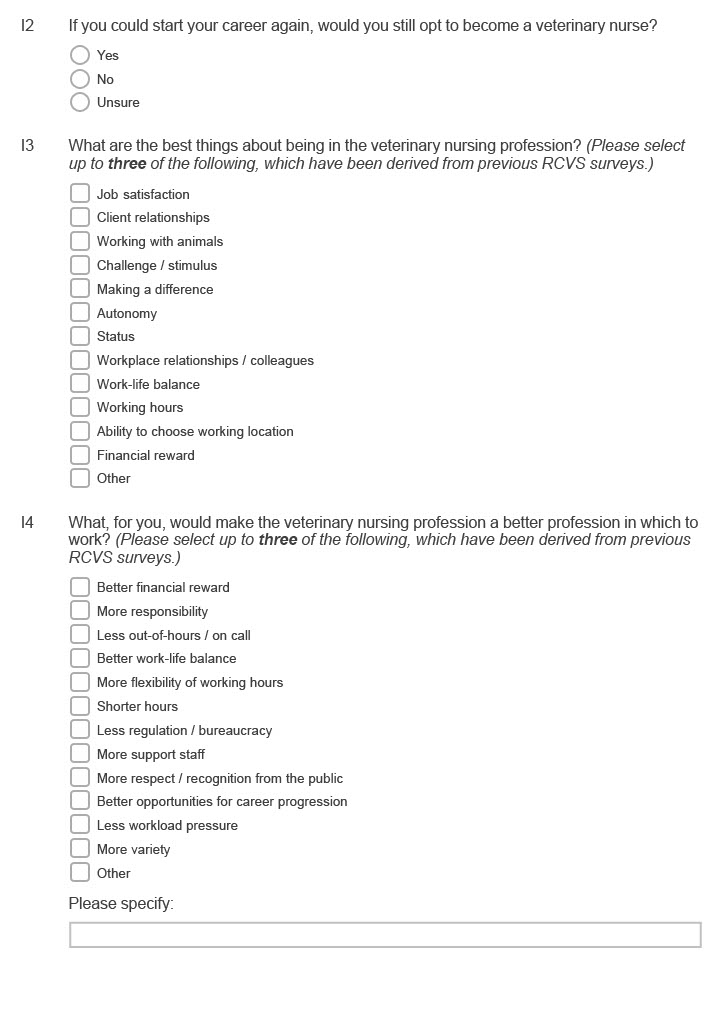
**

**
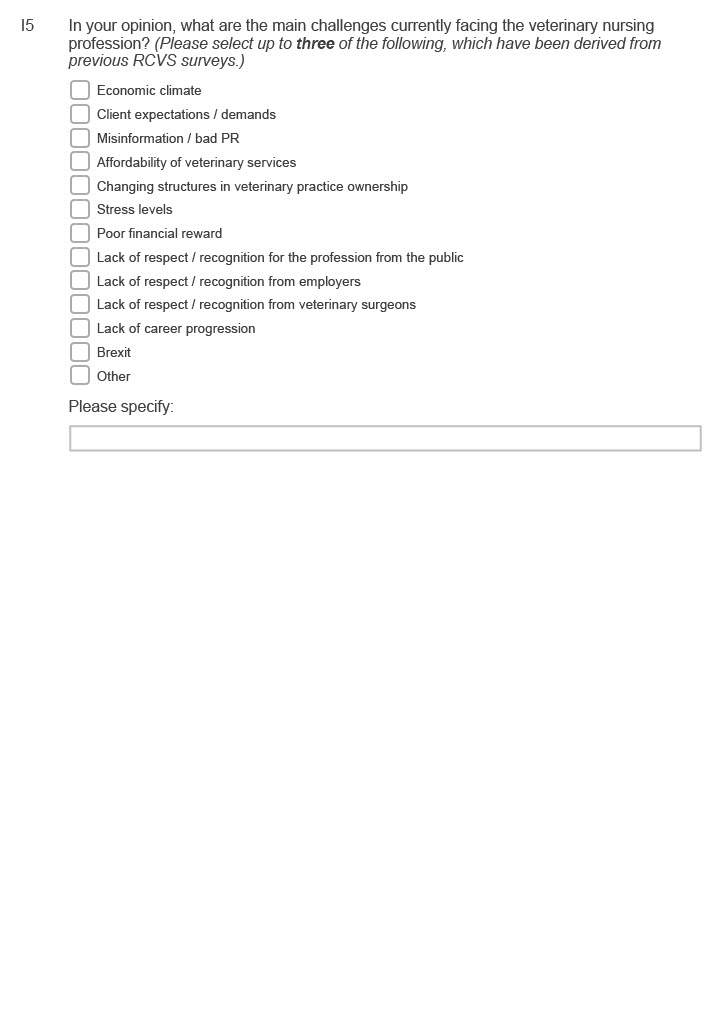
**

**
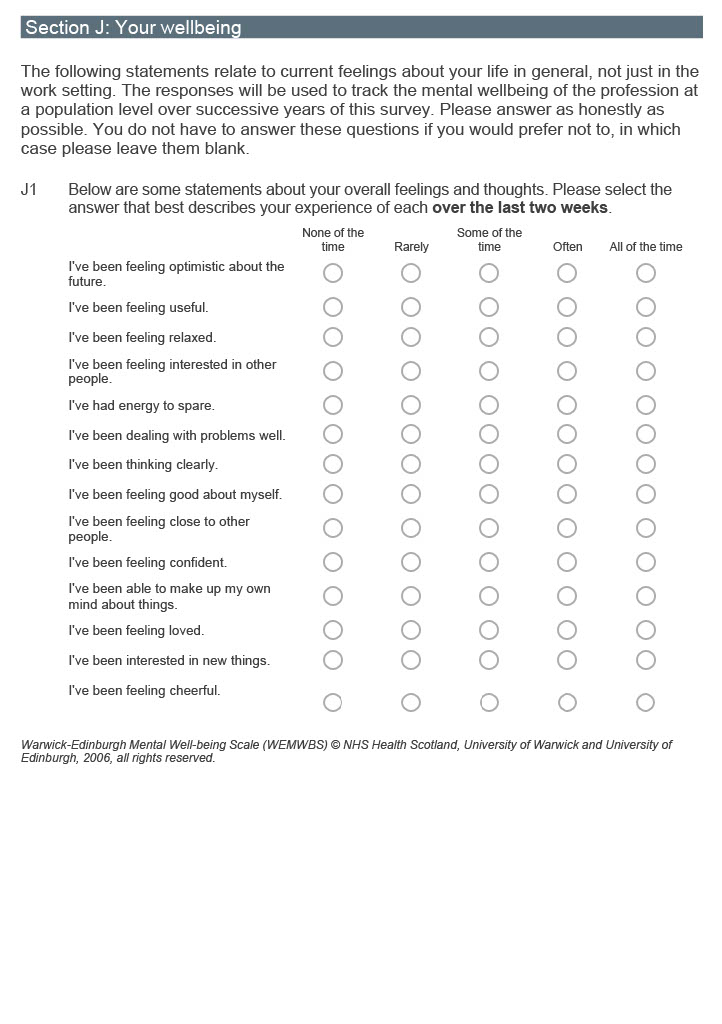
**

**
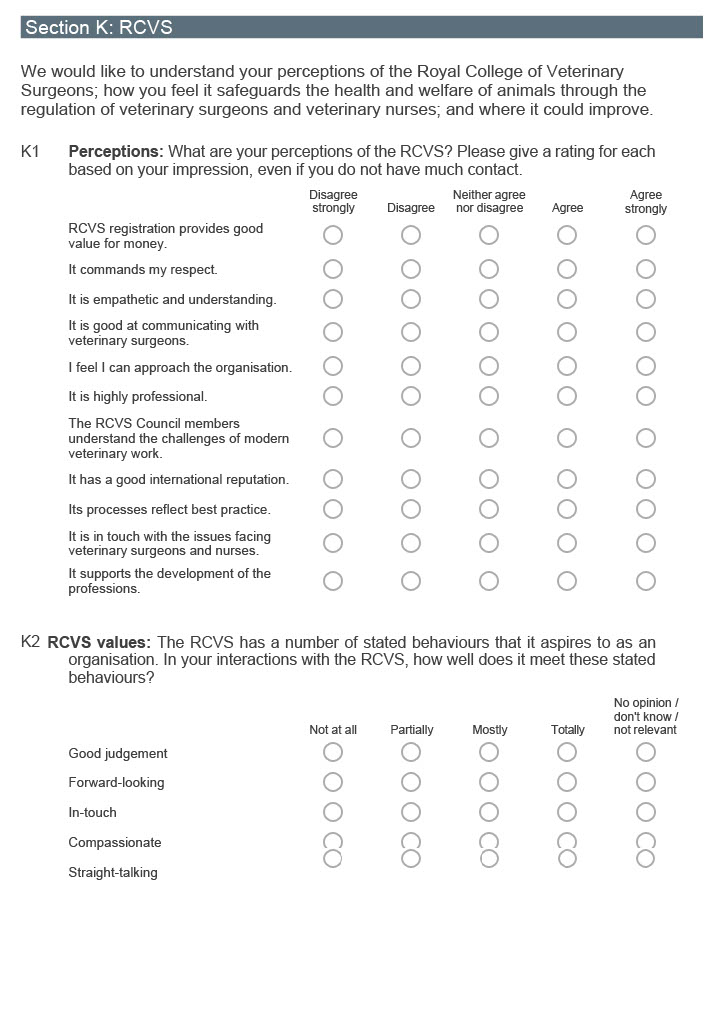
**

**
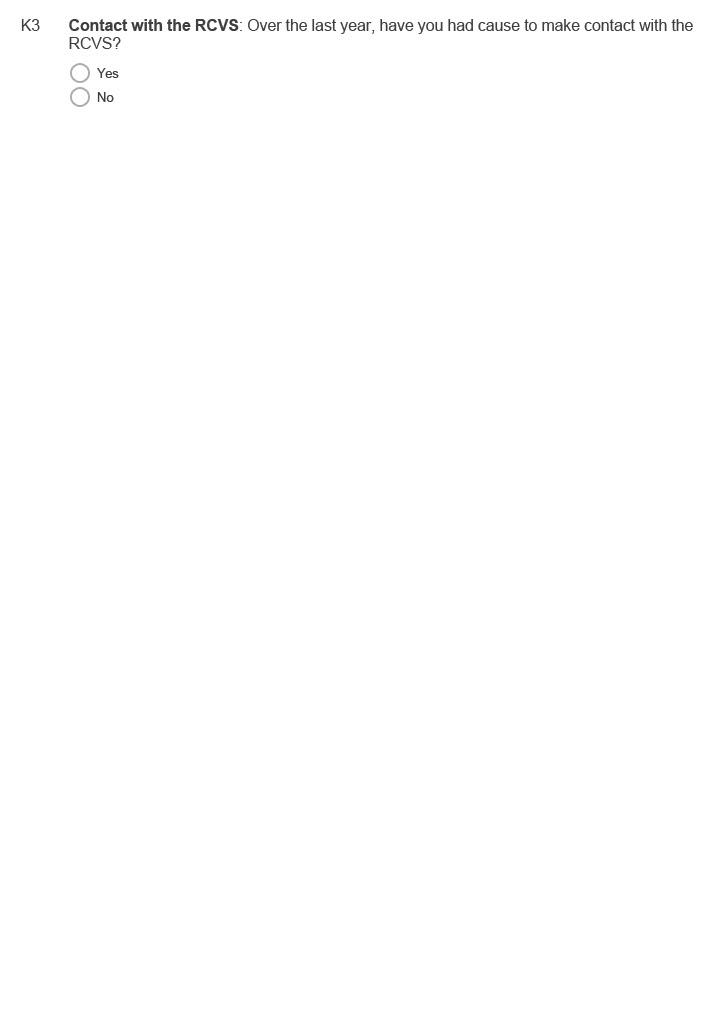
**

**
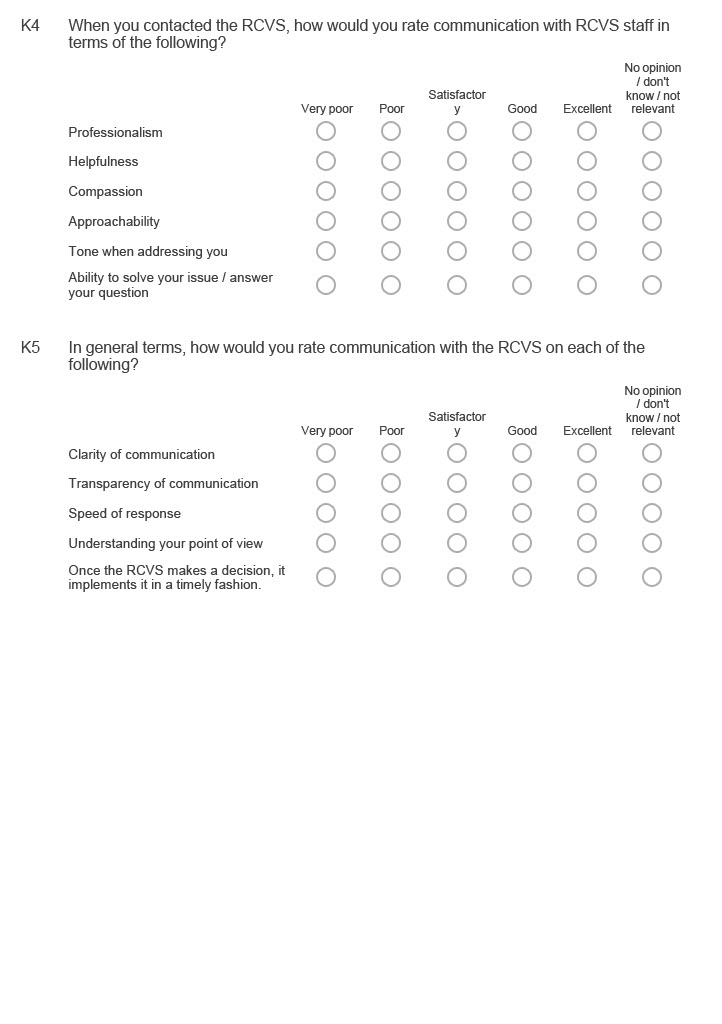
**

**
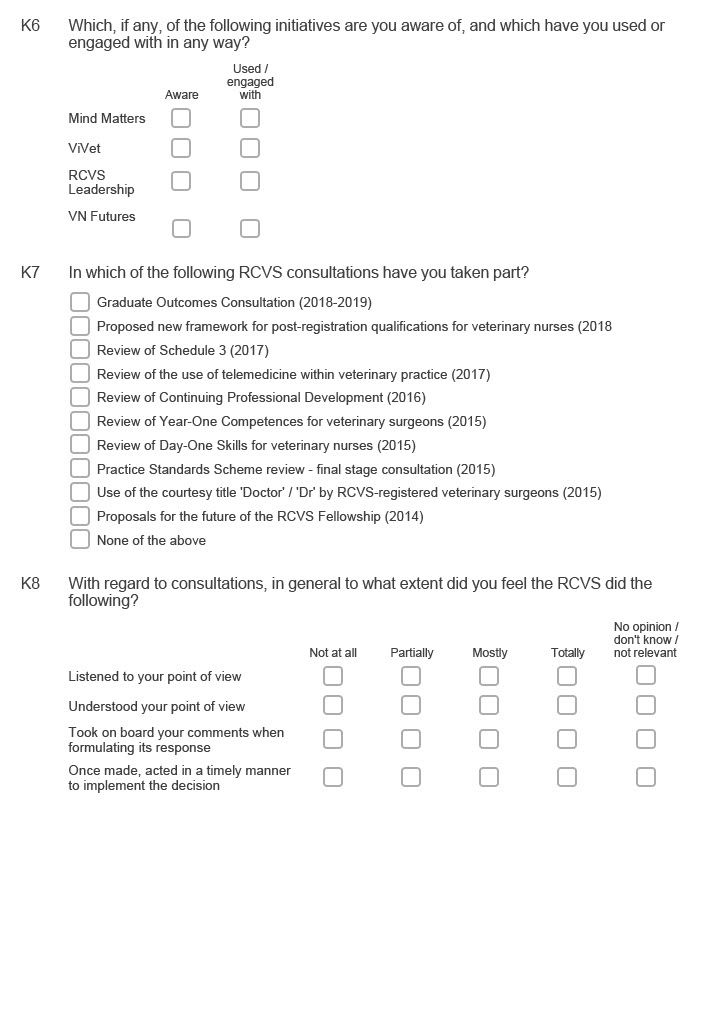
**

**
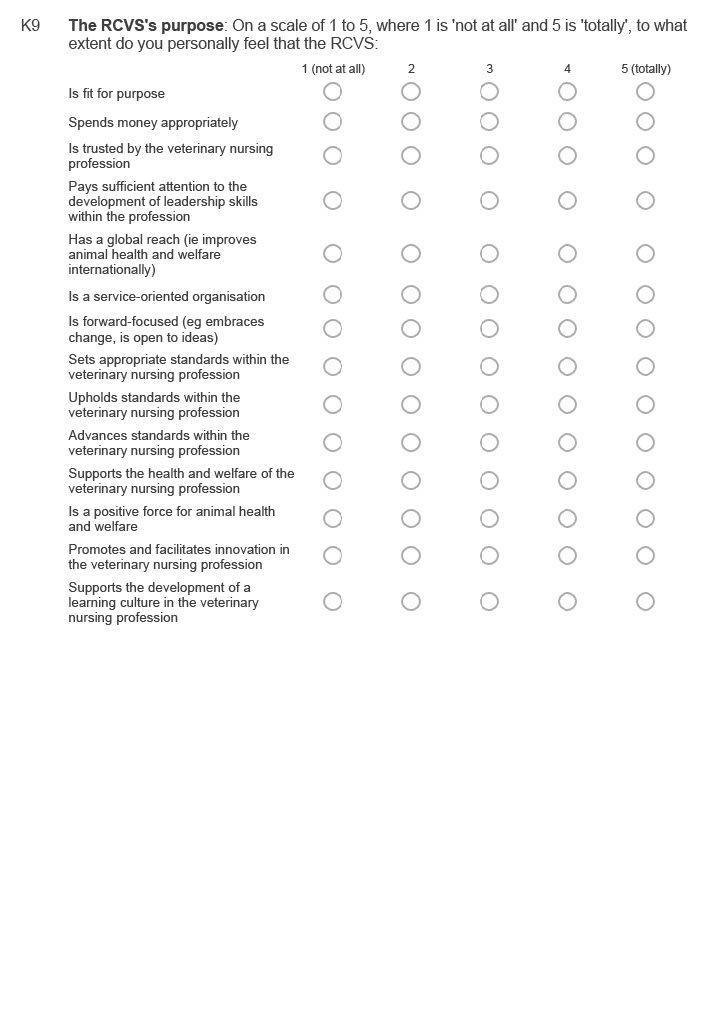
**

**
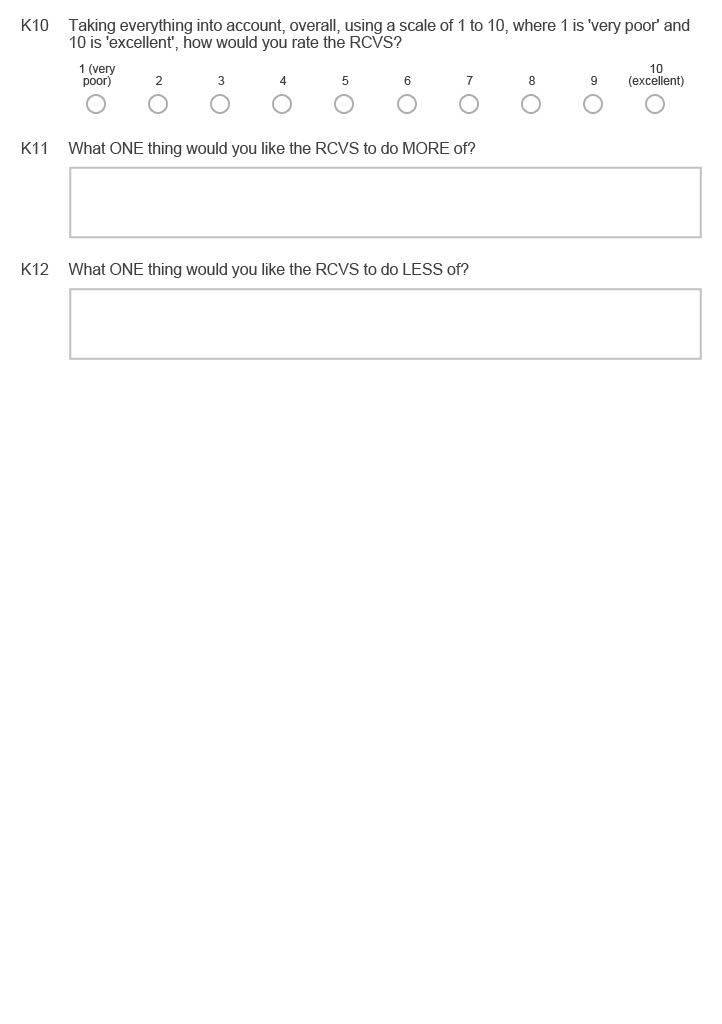
**

**
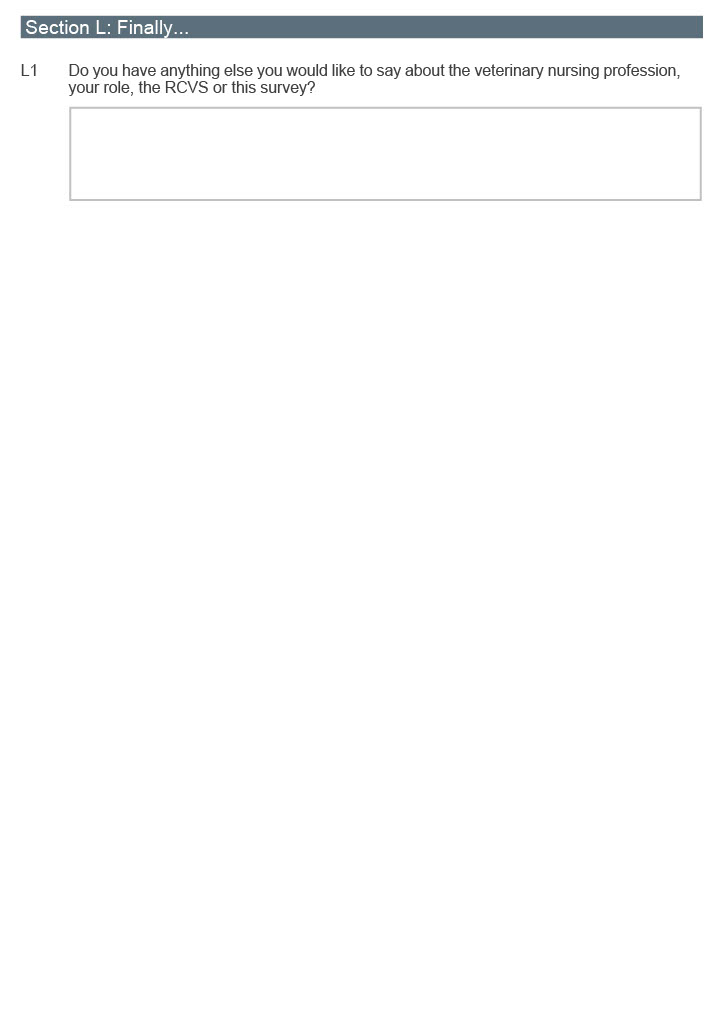
**

**
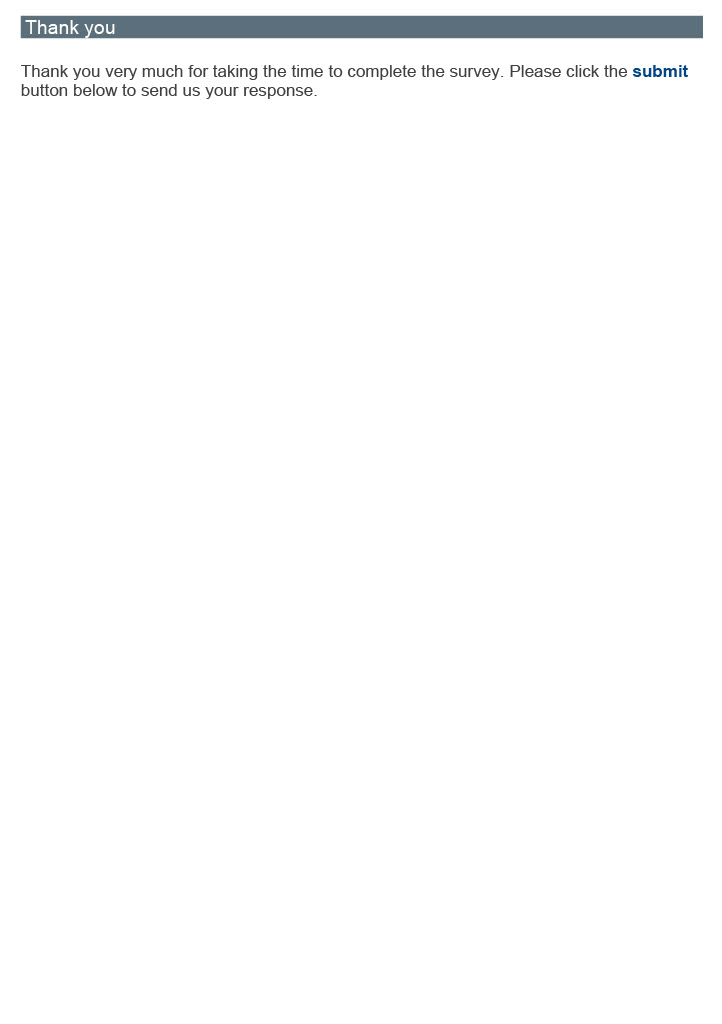
**
